# Dense sampling of ethnic groups within African countries reveals fine-scale genetic structure and extensive historical admixture

**DOI:** 10.1126/sciadv.abq2616

**Published:** 2023-03-29

**Authors:** Nancy Bird, Louise Ormond, Paschal Awah, Elizabeth F. Caldwell, Bruce Connell, Mohamed Elamin, Faisal M. Fadlelmola, Forka Leypey Matthew Fomine, Saioa López, Scott MacEachern, Yves Moñino, Sam Morris, Pieta Näsänen-Gilmore, Nana Kobina Nketsia V, Krishna Veeramah, Michael E. Weale, David Zeitlyn, Mark G. Thomas, Neil Bradman, Garrett Hellenthal

**Affiliations:** ^1^ Department of Genetics, Evolution and Environment, University College London Genetics Institute (UGI), University College London, London, UK.; ^2^ Faculty of Arts, Letters and Social Sciences, University of Yaoundé I, Yaoundé, Cameroon.; ^3^ The Library, Lancaster University, Lancaster, UK.; ^4^ Linguistics and Language Studies Program, York University, Toronto, Ontario, Canada.; ^5^ University Hospital of Derby, Derby, UK.; ^6^ Kush Centre for Genomics and Biomedical Informatics, Biotechnology Perspectives Organisation, Khartoum, Sudan.; ^7^ Department of History and African Civilisations, University of Buea, Buea, Cameroon.; ^8^ Wellcome Trust, London, UK.; ^9^ Division of Social Science, Duke Kunshan University, Kunshan, China.; ^10^ LLACAN, CNRS, Paris, France.; ^11^ Nuffield Department of Population Health, University of Oxford, Oxford, UK.; ^12^ Tampere Centre for Child, Adolescent and Maternal Health Research: Global Health Group, Faculty of Medicine and Health Technology, Tampere University, Tampere, Finland.; ^13^ Department for Health Promotion, Finnish Institute for Health and Welfare, Helsinki, Finland.; ^14^ Essikado Traditional Council, Essikado, Ghana.; ^15^ Department of Ecology and Evolution, Stony Brook University, Stony Brook, NY, USA.; ^16^ Genomics PLC, Oxford, UK.; ^17^ School of Anthropology and Museum Ethnography, University of Oxford, Oxford, UK.; ^18^ Henry Stewart Group, London, UK.

## Abstract

Previous studies have highlighted how African genomes have been shaped by a complex series of historical events. Despite this, genome-wide data have only been obtained from a small proportion of present-day ethnolinguistic groups. By analyzing new autosomal genetic variation data of 1333 individuals from over 150 ethnic groups from Cameroon, Republic of the Congo, Ghana, Nigeria, and Sudan, we demonstrate a previously underappreciated fine-scale level of genetic structure within these countries, for example, correlating with historical polities in western Cameroon. By comparing genetic variation patterns among populations, we infer that many northern Cameroonian and Sudanese groups share genetic links with multiple geographically disparate populations, likely resulting from long-distance migrations. In Ghana and Nigeria, we infer signatures of intermixing dated to over 2000 years ago, corresponding to reports of environmental transformations possibly related to climate change. We also infer recent intermixing signals in multiple African populations, including Congolese, that likely relate to the expansions of Bantu language–speaking peoples.

## Teaser

Historical events may have played an appreciable role in shaping people’s genomes within African countries.

## INTRODUCTION

Since the advent of genome-wide genotyping and sequencing, the number of studies analyzing African autosomal genomes has lagged behind those of genomes from other continents, particularly Europe (*1*, *2*). This is despite the fact that African genomes contain more genetic variation and often display a high degree of genetic structure, relative to non-Africans (*3*). In recent years, there have been multiple developments in genetic diversity research in Africa, with studies providing autosomal data from almost every country and all major linguistic phyla (*4*–*6*). These studies have shown that genetic structure often correlates with geography and linguistics at both broad (*6*) and fine scales (*7*), with some evidence that cultural factors also have an impact (*8*, *9*). In addition, studies of African ancient DNA (aDNA) from multiple time periods and within different archaeological contexts have revealed the presence of deep population structure, some of which has been overlain by more recent migrations (*10*–*14*).

Previous analyses of African genomes have shown that admixture between geographically disparate populations plays an important role in shaping patterns of genetic diversity (*15*). For example, studies have inferred the presence of West Eurasian–related ancestry in Northeast Africa [e.g., Sudan (*16*, *17*) and Ethiopia (*1*, *8*, *18*, *19*)], gene flow across the Sahara [e.g., Republic of The Gambia and Burkina Faso (*20*) and Chad (*21*)], and longitudinal migrations below the Sahara [e.g., observed in the Fulani/Foulbe (*22*) and Nilo-Saharan speakers (*1*)]. The expansion of Bantu language–speaking peoples from the Cameroon/Nigeria border region throughout much of sub-Saharan Africa beginning roughly 3500 years before present (B.P.) radically reshaped the genetic structure of the continent (*5*) and led to extensive admixture between migrants and local populations (*15*, *23*). Admixture at much more local scales has also been inferred, often correlating with geographical proximity and shared cultural practices (*8*). Advances in the precision of dating admixture events (*24*–*27*) have allowed inference about the impact that past events, such as the formation of empires (*28*), expansions (*16*), or migrations (*23*), may have had on the genetic diversity of present-day African populations.

Despite these advances, studies of African genetic diversity are often limited by sparse sampling of ethnic groups and/or geographic regions, reducing their ability to detect such fine-scale genetic structure as has been reported in other continents [e.g., within countries in Europe (*29*)]. There have been some recent advances in the study of fine-scale structure in northeastern (*8*, *16*), central (*23*), and southern (*7*, *30*) Africa, but many countries and regions have gone understudied. An understanding of the level of genetic structure within smaller regions may be essential for population stratification correction in large-scale genome-wide association studies (GWAS) (*7*, *31*). In addition, better estimation of patterns of linkage disequilibrium (LD) in a region may improve methods of imputation, fine-mapping, colocalization, and polygenic risk scores that rely on this inference (*32*). Furthermore, groups in the same region often have vastly different histories and signatures of admixture (*8*, *21*). Without dense sampling of groups, a comprehensive understanding of the genetic history of a region is impossible.

**Fig. 1.**
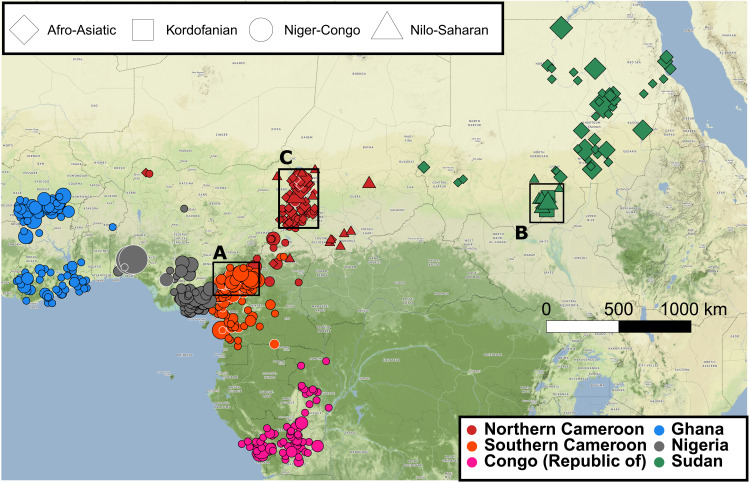
Location of samples from Cameroon, Republic of the Congo, Ghana, Nigeria, and Sudan. White symbol outline indicates the sample has been previously published (*8*, *12*, *41*, *43**)*. For newly reported samples, individuals are plotted using the mean birthplace of the individual’s maternal grandmother and paternal grandfather (*n *= 1333; see Materials and Methods). Symbol size represents the number of individuals sampled with mean birthplace at that location, and symbol shape represents the individual’s first language family. Kordofanian speakers (square) are only found in southern Sudan (box B). Cameroonians have been separated into northern Cameroon (dark red) and southern Cameroon (lighter red) based on the individual’s birthplace and genetic clustering results (Fig. 3). Squares (A, B, and C) indicate some of the regions mentioned in the main questions that we address.

Here, we analyze newly acquired genetic variation data at 510,615 single-nucleotide polymorphisms (SNPs) from 1387 people, with the majority of samples from five African countries: Cameroon, Republic of the Congo, Ghana, Nigeria, and Sudan, as well as 54 samples from three other countries (Table 1). The new data include people from 166 distinct, self-reported ethnic groups, comprising speakers of three of the four major language phyla in Africa (Afro-Asiatic, Niger-Congo, and Nilo-Saharan), as well as some putative language isolates in the South Kordofan region of Sudan (see fig. S1 for maps of mean birthplace for each ethnic group and text S1 for a brief description of ethnic groups and languages sampled in each country) (*33*, *34*). Sampled individuals span east to west across the African continent and occupy a wide variety of environments, encompassing individuals from the Nile Valley and the mountains of southern Sudan to inhabitants of the Congo rainforest (Fig. 1 and data S1). Examples of previously undersampled groups or regions in these data include dense sampling of Afro-Asiatic Chadic speakers from northern Cameroon (Fig. 1, box C), ethnic groups from the Grassfields region of Cameroon (Fig. 1, box A), and ethnic groups from the South Kordofan region of Sudan (Fig. 1, box B).

**Table 1.** Number of samples from each country in the newly reported dataset after quality control.

Country	Number of samples
Cameroon	484
Republic of the Congo	114
Ghana	211
Nigeria	291
Sudan	233
Mozambique	11
South Africa	35
Zimbabwe	8

These data enable insights into the genetic history of peoples in this region. For brevity, here, we focus on the following questions, which are each detailed briefly below (with more information in texts S1 and S2):

1) Does genetic structure vary with geography, language, and/or ethnic group within each of Cameroon, Republic of the Congo, Ghana, Nigeria, and Sudan, as has been observed within other African countries (*1*, *7*, *8*)?
2) The Grassfields of Cameroon, broadly located in the Northwest and West regions (Fig. 1, box A), has a long and complex history of multiple polities of different sizes (*35*). Does genetic structure correlate with these historical polities?
3) Some of the Kordofanian languages, spoken in the South Kordofan region of Sudan, have been placed within the Nilo-Saharan language phylum (Fig. 1, box B triangles). Linguists have debated the placement of other Kordofanian languages (Fig. 1, box B squares), with some suggesting that they should be placed within the phylum containing Niger-Congo speakers, while others arguing that they are linguistic isolates (*36*). Do genetic data distinguish between these two language categories?
4) The Arabic expansion into Africa began during the seventh century CE in Egypt but did not reach other regions until later (*37*). Do we find evidence of Arabic admixture in Sudan and Cameroon, and can we date any such admixture?
5) The Kanem-Bornu empire, beginning in roughly 700 CE, was a large trading polity spanning present-day northern Cameroon, northern Nigeria, and Chad (*38*) (see text S2 for more information). Is there evidence of admixture that is correlated with the dates and trading networks of the empire in two ethnic groups sampled in Cameroon: Kanuri and Kotoko, who were historically associated with it?
6) The Fulani are a pastoralist ethnic group who inhabit a large segment of the Sahel belt from coastal West Africa to Sudan and speak a language closely related to those spoken in Senegal. Previous studies of Fulani from other countries have inferred them to be admixed descendants of populations related to Moroccans and West Africans (*15*, *20*). Do sampled Fulani from Cameroon show similar admixture signals to those previously reported in genetic studies of Fulani from other countries?
7) Afro-Asiatic Chadic languages are spoken in Chad, northern Cameroon, and northeastern Nigeria, although their closest relative within the Afro-Asiatic phyla is debated (Fig. 1, box C diamonds) (*39*). Previous studies of Chadic speakers using a small number of loci have inferred large amounts of recent ancestry related to Nilo-Saharan speakers (*1*). Can we replicate this signature of Nilo-Saharan–like ancestry in a set of 97 sampled Chadic-speaking individuals analyzed here? Can we identify which sampled Afro-Asiatic–speaking group they are most recently related to?
8) The Bantu languages are hypothesized to have developed in the Nigeria/Cameroon border region before peoples speaking the languages expanded southward and eastward, beginning roughly 3500 years ago (*23*, *40*). There is debate over the route of the expansion throughout sub-Saharan Africa, as well as the number of expansions, with recent data from the Congo basin suggesting multiple waves. Leveraging new dense sampling from the proposed “cradle of the Bantu languages,” can we provide details regarding the source, routes, and timing of the expansions of Bantu-speaking peoples?

To address these questions, we analyzed these data together with published resources containing genetic variation data of individuals from 287 present-day worldwide populations (*4*, *6*, *8*, *11*, *12*, *41*–*45*) and from 20 high-coverage ancient African individuals (*10*, *12*, *13*), including a Later Stone Age individual from the Shum Laka rock shelter in western Cameroon (data S2 and fig. S2). For each sampled individual with newly reported genetic variation data, information about their self-reported ethnic group, birthplace, first and second language, and that of their parents, maternal grandmother, and paternal grandfather was documented, with both recorded grandparents sharing the same ethnic group and birthplace in the majority of cases (80%). This acts to mitigate the impact of recent migration or admixture on inferences about population structure and ancestry, ameliorating a major potential confounder when addressing the questions above (*29*). We note that when we mention “ancestry” related to a particular identifier (e.g., a geographic region or linguistic group), we are referring to genetic variation patterns that match those of sampled individuals with that identifier, a shorthand that we use for convenience.

## RESULTS

### Genetic structure is associated with geography, language, and ethnic group

**Fig. 2.**
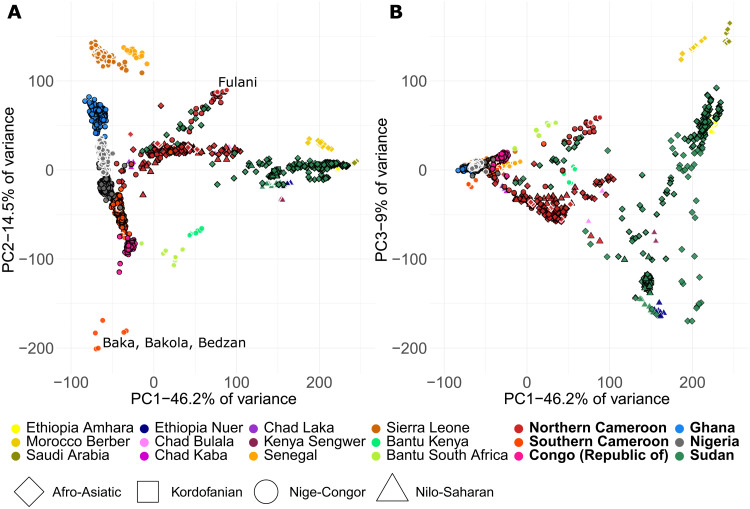
Haplotype-based PCA using patterns of recent ancestor sharing among individuals. (**A**) PC1 versus PC2. (**B**) PC1 versus PC3. Samples newly reported in this study are indicated by symbols with a black outline, previously published samples have a white outline (*4*, *6*, *8*, *12*, *41*–*44*), and language family is represented by symbol shape. Some outlying populations (Cameroonian Baka, Bakola, Bedzan, and Fulani) are labeled.

We used multiple different methods to analyze genetic structure within the dataset (see flowchart in fig. S3). To visualize the main axes of genetic diversity, we initially performed a principal components analysis (PCA) (*46*) on patterns of inferred recent ancestor sharing among individuals (*47*). Specifically, for each of the 5253 individuals, we first used the haplotype-based program ChromoPainter (*47*) to infer the genome-wide proportion of DNA for which each individual shares a most recent ancestor with sampled people from each of 260 worldwide populations (fig. S1 and text S3). We then performed a PCA on these inferred proportions across the 1333 of these individuals from Cameroon, Ghana, Nigeria, Republic of the Congo, and Sudan, incorporating data from a selection of other African groups and Saudi Arabians for comparison (Fig. 2). We additionally performed the more commonly used PCA of genotype data using smartPCA (fig. S4, A and B) but observed a stronger correlation between genetics and geography (*r* = 0.84 versus *r* = 0.59) when applying Procrustes superimposition to the haplotype-based analysis (figs. S4, C and D, and S5), as has been reported previously (*8*, *31*). We also applied the clustering algorithm ADMIXTURE (*48*) to highlight broad genetic patterns (fig. S6).

The first PC forms a cline from Saudi Arabians to West African Niger-Congo speakers, (Fig. 2). Most West African Niger-Congo speakers included in this analysis lie along the second PC in a manner mirroring geography, from Sierra Leoneans (top left) to Congolese (Fig. 2A), although the Cameroonian Fulani are a notable outlier, reflecting ADMIXTURE results at *K* = 8 (fig. S6). Rainforest hunter-gatherers from Cameroon (Baka, Bakola, and Bedzan) also cluster separately from other Cameroonians and Congolese (bottom left of Fig. 2A, pink in ADMIXTURE at *K* = 4), as do South African and Kenyan Bantu language speakers. On PC3, Nilo-Saharan– and Kordofanian-speaking Sudanese cluster tightly together near the Nilo-Saharan–speaking Ethiopian Nuer (bottom right of Fig. 2B), with most Arabic-speaking Sudanese spreading along PC3 toward Saudi Arabians (top right of Fig. 2B). Afro-Asiatic– and Nilo-Saharan–speaking northern Cameroonians are at an intermediate position between Nilo-Saharan–speaking Sudanese and West Africans.

We then calculated the genetic distance between pairs of (i) ethnic groups, (ii) linguistic groups, and (iii) individuals with birthplaces separated by different distances, to understand how genetics correlates with each of these factors. We measured genetic distance using both the fixation index (*F*_st_) (fig. S7) and a haplotype-based measure, total variation distance (TVD), where we calculated significance using a permutation test to account for differences in sample size (see Materials and Methods, fig. S8, and text S3) (*29*). When using both *F*_st_ and haplotype-based analyses, ethnic groups from the same country are often more genetically similar to each other than they are to any groups from another country, including close neighbors. For example, the six southeastern Nigerian groups are genetically distinguishable from all groups from western Cameroon, despite living in proximity. Within some regions, specifically Ghana, Northwest and West Cameroon, and southern Sudan, ethnic groups are typically significantly genetically different from each other (fig. S8).

When examining genetic distance between language phyla within Cameroon (fig. S9A), Nilo-Saharan and Afro-Asiatic speakers are typically genetically distinguishable from Niger-Congo speakers, consistent with the PCA (Fig. 2). Within Cameroonian Niger-Congo speakers, Narrow Bantu, Grassfields, and Northern Bantoid speakers are all distinguishable, as are the North-Central Atlantic–speaking Fulani. Similarly, Nilo-Saharan–, Kordofanian–, and Afro-Asiatic–speaking groups in Sudan are distinguishable (fig. S9B). In each country, there is a negative relationship between genetic similarity (calculated as 1 − TVD) and geographic distance (fig. S10). However, there is a large variation in correlation strength across countries, with *R*^2^ = 0.10 in Nigeria and *R*^2^ = 0.96 in southern Cameroon. Individuals from Cameroon and Sudan showed the greatest reduction in genetic similarity with distance, which remained even after only comparing people belonging to the same ethnic group (fig. S10B). However, in Sudan, we observed a weaker correlation between genetic similarity and distance when analyzing only Sudanese that were sampled along the Nile River (see text S1 and fig. S11). These correlations can be influenced by isolation by distance or differential admixture (explored below) or a combination of the two.

**Fig. 3.**
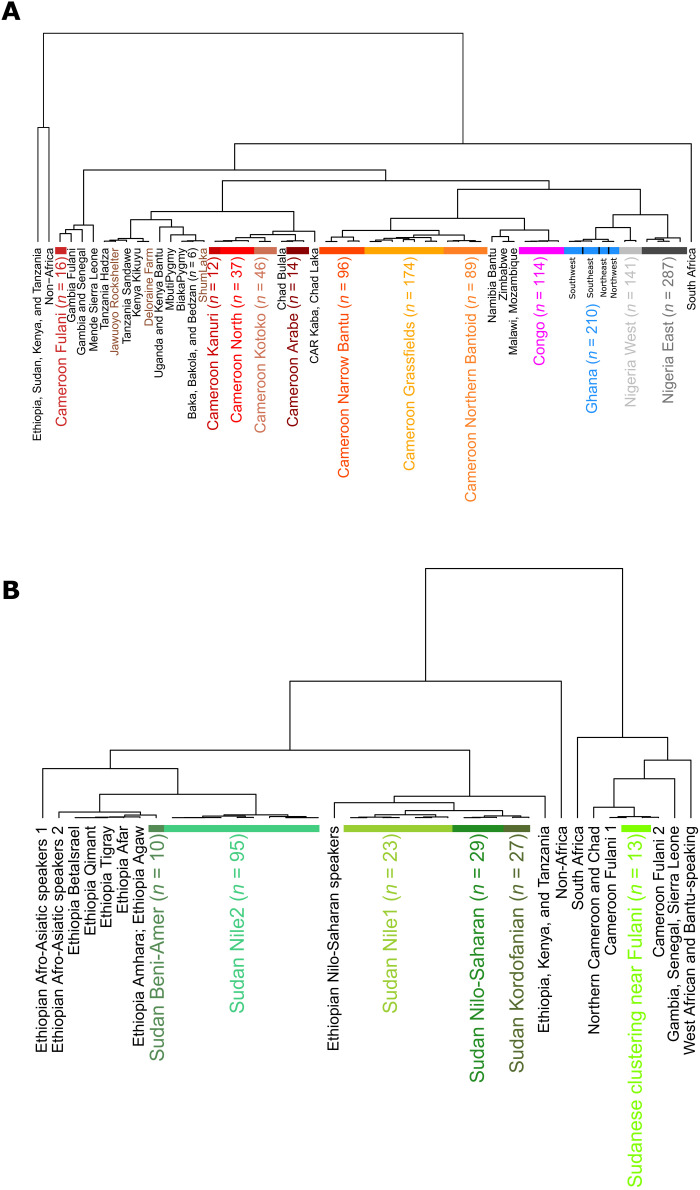
fineSTRUCTURE dendrograms showing the inferred relatedness between clusters of individuals in the dataset. Individuals from Cameroon, Republic of the Congo, Ghana, and Nigeria (**A**) and Sudan (**B**) were left to cluster freely, while individuals from other populations were merged into superindividuals who each correspond to group labels (see Materials and Methods). For clarity, some branches have been condensed (see more detailed dendrograms in figs. S12 and S13). Ancient individuals are shown with a brown label. The 18 final supergroups used for admixture analysis are highlighted with different label colors, and the sample sizes of these clusters are shown in brackets. The clusters shown in Fig. 4 are those on the branches labeled as Ghana (Fig. 4A), Cameroon Northern Bantoid and Cameroon Grassfields (Fig. 4D), Sudan Nilo-Saharan, and Sudan Kordofanian (Fig. 4C). The branches within the Ghana supergroup that correspond to different geographic regions in Fig. 4A have been labeled.

**Fig. 4.**
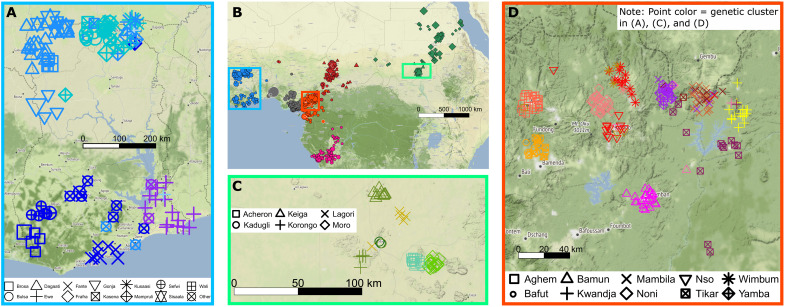
Fine-scale genetic structure is associated with geographic region and ethnic group. (**A**) Ghana, (**C**) the South Kordofan region of Sudan, and (**D**) southern and western Cameroon, where colors depict genetic cluster inferred with fineSTRUCTURE (fig. S12). Symbols indicate each individual’s ethnic group. (**B**) Adapted from Fig. 1 and highlighting the location of each of the other panels. In Ghana, genetic structure corresponds with geographic region, while, in the other two regions, clusters are also strongly associated with ethnic group. In (C) and (D), very fine-scale structure is observed, with some clusters less than 20 km apart from each other. Sample locations are slightly jittered to avoid overlap.

We next used fineSTRUCTURE (*29*) to group the individuals into clusters (see Materials and Methods) and infer a dendrogram of genetic relatedness among them (Fig. 3, figs. S12 and S13, and data S3). Matching PCA results (fig. S4C), individuals from Ghana show clear structure by geography, with an initial split (i.e., higher in the fineSTRUCTURE-inferred dendrogram) dividing northern and southern samples, respectively, followed by splits between eastern and western Ghanaians in both the north and south (Figs. 3 and 4A). In Nigeria, southwestern ethnic groups (Yoruba and Esan) cluster more closely to Ghanaians than to southeastern Nigerians on the fineSTRUCTURE dendrogram. No clear splits related to ethnic group or geography are inferred among southeastern Nigerians in this sample. Similarly, in the Republic of the Congo, there is limited genetic structure, although the Yombe form a distinct cluster. Northern Cameroonians cluster on one branch with populations from Chad and the Central African Republic, apart from the Fulani who cluster on a branch with coastal West Africans. While certain northern Cameroonian ethnic groups, such as the Arabe, Kanuri, and Kotoko form their own distinct clusters, all others ethnic fall into two large clusters. However, we caution that sample size can have an impact on fineSTRUCTURE inferences, in that the larger the sample size the more likely it is to detect any genetic structure present.

### Fine-scale genetic structure correlates with ethnic group in the Grassfields of Cameroon

Southern Cameroonians form three main genetic clusters that are broadly, but not completely, defined by language group: Northern Bantoid speakers, Grassfields speakers, and Narrow Bantu speakers (Fig. 3 and fig. S12). Exceptions include the Yamba who speak a Grassfields language but cluster with Northern Bantoid speakers, some of the Mbo who speak a Narrow Bantu language but cluster with Grassfields speakers, and some of the Bamileke who speak a Grassfields language but cluster with Narrow Bantu speakers. Notably, within Northern Bantoid and Grassfields speakers, fineSTRUCTURE distinguished ethnic groups living <20 km from each other (Fig. 4D). In contrast, Narrow Bantu speakers show almost no genetic structure associated with ethnic group, consistent with our TVD analysis (fig. S8). The high-coverage Later Stone Age individual from the Shum Laka rockshelter in Cameroon (dated 8000 years B.P.) clusters on the same branch as the Biaka from the Central African Republic and the Baka, Bakola, and Bedzan from Cameroon in the fineSTRUCTURE dendrogram (Fig. 3). This fits with previous findings that genetic variation patterns in this ancient individual are more similar to those of present-day rainforest hunter-gatherers than those of Narrow Bantu and Grassfields language speakers from the Grassfields of Cameroon (*12*).

### Nilo-Saharan and Kordofanian speakers from the south Kordofan region of Sudan are genetically distinct

Nilo-Saharan speakers and Kordofanian speakers from the South Kordofan region of Sudan form separate clusters, on a branch with other clusters containing Sudanese Arabic speakers and Nilo-Saharan speakers from Ethiopia (Fig. 3). Clusters in the South Kordofan region display a notable correspondence with self-described ethnic affiliation and language (Fig. 4C), except for the Keiga and Korongo who cluster together (fig. S13). However, we infer a relatively low correlation with geography (Procrustes Pearson correlation = 0.25; fig. S5H).

Sudanese outside of the South Kordofan region were divided into four major clusters. First, one ethnic group, the Beni-Amer, forms their own cluster. Another group of individuals from a variety of different ethnic groups cluster on the same branch as the Fulani from Cameroon. The remaining individuals are then divided into two main genetic clusters that show very little correspondence to ethnic group or geography but, instead, exhibit differing amounts of inferred admixture related to non-Africans (see below).

### Defining groups for identity-by-descent sharing, recent shared ancestry, and admixture analyses

We defined 101 “clusters” containing individuals from Cameroon, Republic of the Congo, Ghana, Nigeria, and Sudan, where individuals in each group have the same self-reported ethnicity and cluster together using fineSTRUCTURE (figs. S12 and S13 and data S4). To explore relative degrees of genetic homogeneity among groups, which may be indicative of relative degrees of isolation, we used hap-ibd (*49*) to calculate the total length of identity-by-descent (IBD) segments shared between each pair of individuals within each cluster. The mean inferred genome-wide IBD sharing varied from <3 to 241 cM among clusters, with the highest value observed in the Fulani (fig. S14). High values were also seen in some other Cameroonian and Sudanese clusters.

We used SOURCEFIND (*50*) to infer how individuals from each of the 101 clusters relate genetically to 226 reference populations (fig. S15). We inferred that the majority of genetic variation in individuals from Republic of the Congo, Ghana, Nigeria, and southern Cameroon is recently related to West African and Bantu-speaking groups, while genetic variation patterns in individuals from northern Cameroon and Sudan are best described as mixtures of those in East Africans, West Africans, and North Africans. We used these SOURCEFIND results along with higher levels of the fineSTRUCTURE dendrogram to merge the 101 clusters into 18 “supergroups” who cluster together and show similar inferred relatedness to these 227 reference populations (see Fig. 3 for supergroups and sample sizes, fig. S3 for flowchart, and data S5). Using these larger groups of individuals increases our power to detect and date admixture events.

**Fig. 5.**
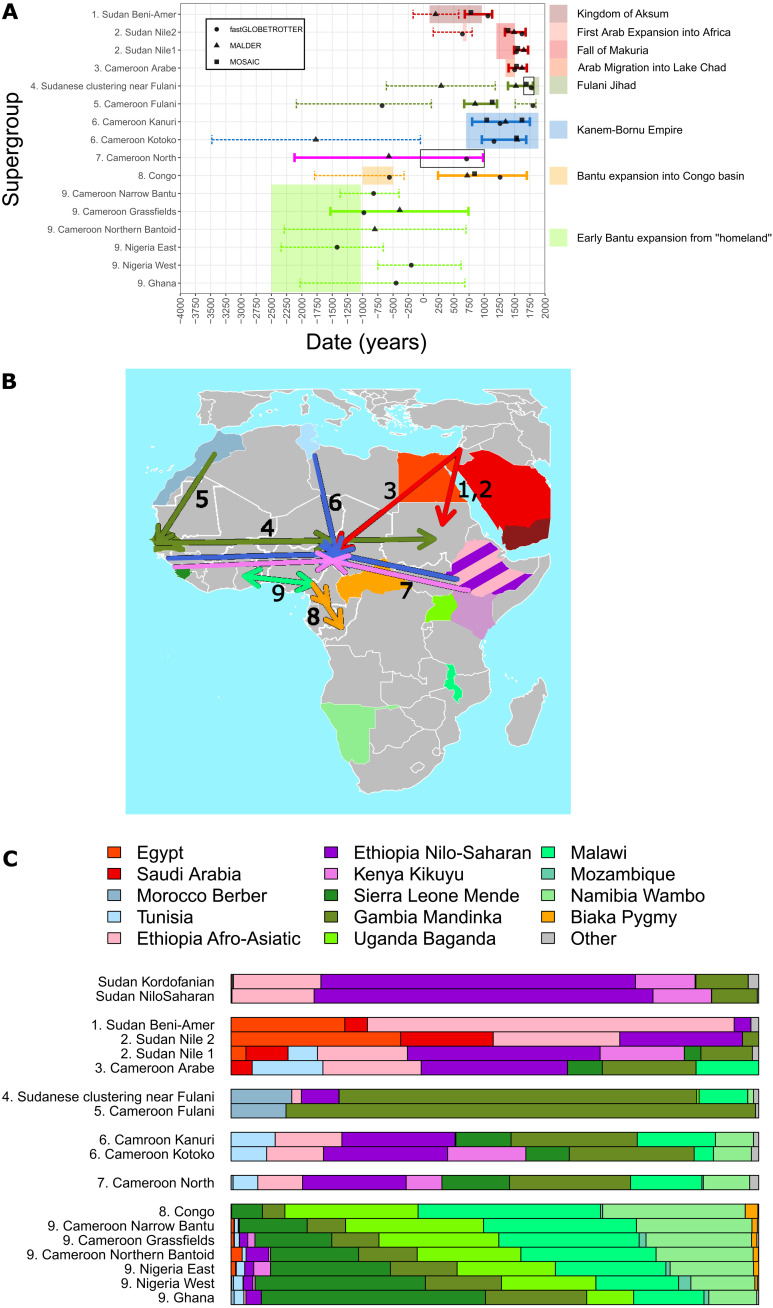
Inferred admixture event(s) and recent ancestor sharing. (**A**) Inferred admixture dates using fastGLOBETROTTER (circle), MALDER (triangle), and MOSAIC (square) and 95% confidence intervals (defined as the union of confidence intervals for all three methods) for each supergroup, except Sudan Kordofanian and Sudan Nilo-Saharan. For each confidence interval separately, see fig. S16. Events inferred by only one method with confidence intervals not within three generations of those of the other methods are shown with a dashed line. The dates of historical events occurring at the same time and in the same region as each admixture event are shown as transparent boxes, legend at right. Point and error bar color corresponds with arrows in (B). The black open rectangles span the confidence intervals inferred by fastGLOBETROTTER for the Cameroon North supergroup, and the confidence intervals inferred by MOSAIC and fastGLOBETROTTER for the Sudanese clustering near Fulani supergroup, both discussed below. (**B**) Map with arrows indicating potential waves of migration related to inferred admixture events, with color and number of arrow denoting admixture category shown in (A). (**C**) Description of each supergroup’s genetic variation patterns as mixtures of those in the reference populations given at the top [colors corresponding to those on map in (B)], inferred by SOURCEFIND. Ethiopian Afro-Asiatic–speaking clusters and Nilo-Saharan–speaking clusters have been grouped for ease of visualization (see data S6).

We then applied three separate methods: MALDER (*26*), fastGLOBETROTTER (*27*), and MOSAIC (*25*) to infer admixture separately in each of the 18 supergroups (see Materials and Methods) assuming a pulse model. For this analysis, groups from Cameroon, Republic of the Congo, Ghana, Nigeria, and Sudan were not used as potential surrogates for admixture sources, as doing so can mask signals of admixture shared among these populations. Admixture was inferred by at least one method in all supergroups and inferred with two or more methods in 12 of the supergroups. Date estimates ranged from 2650 BCE to 1800 CE (Fig. 5 and data S6), with overlapping confidence intervals for at least two of the three methods in 10 cases (fig. S16). We note that admixture events between populations who are more genetically divergent will be easier to detect. Since our supergroup sample sizes vary widely, our power to detect and date admixture events and, thus, our confidence intervals will also vary. Likewise, for some admixture events, the dataset may lack suitable reference proxy populations, which will also have an effect on power. Therefore, we treat dates with larger confidence intervals or discrepancies between methods with caution.

### Inferring admixture in northern Cameroonians

**Fig. 6.**
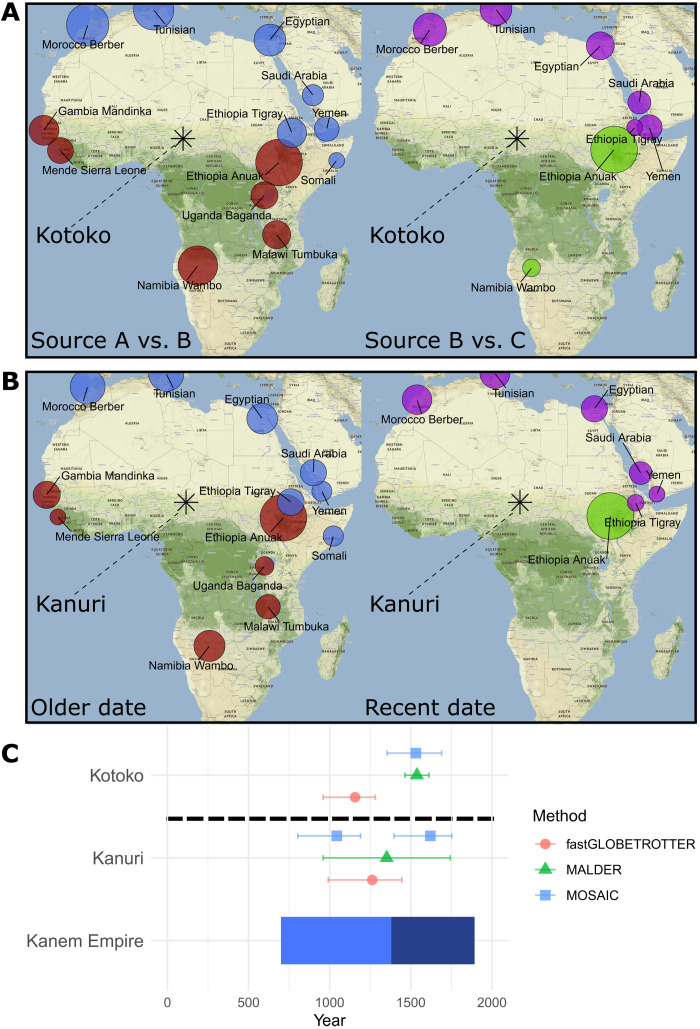
Inferred admixture in the Kotoko and Kanuri correlates with the Kanem-Bornu empire (700 to 1890 CE). MOSAIC results for the Kotoko (**A**) and Kanuri (**B**) indicate admixture between southern and western African (red), eastern African (red and green), and North African/Levantine/Arabian (blue and purple) related sources. Size of point indicates the percentage contribution of that population to the inferred genetic makeup of the admixing source. The mean birthplace of sampled Kotoko and Kanuri is shown with a star. (**C**) Inferred dates of admixture using all three methods (shape and color) are shown in the bottom panel, with 95% confidence intervals. These inferred dates of admixture correspond closely with the span of the Kanem-Bornu empire, both during the early phase when the center was located in southwestern Chad (light blue on the timeline) and the later phase when the center was located in northeastern Nigeria (dark blue on the timeline) (*38*). The Kotoko and the Kanuri ethnic groups were both associated with the empire, which was involved in trade between different regions of Africa.

In non-Kotoko Chadic-speaking Cameroonians (the “Cameroon North” supergroup), fastGLOBETROTTER inferred a multiway admixture event between sources related to (i) coastal West Africans, (ii) East Africans, (iii) Bantu speakers, and (iv) populations from Chad, dated to 710 CE (10 BCE to 840 CE). This date overlaps with that inferred by MALDER but with more precise confidence intervals. In contrast, for the Chadic-speaking Kotoko, as well as the Nilo-Saharan–speaking Kanuri, we inferred more recent (point estimates post-1000 CE) multiway admixture using all three methods. The inferred admixture events for these two groups involve a West African–like and an East African–like source, as well as a third source related to North African, Levantine, and Arabian groups, with dates overlapping the Kanem-Bornu empire (700 to 1890 CE; Fig. 6). MALDER additionally inferred an older admixture event in the Kotoko.

In the Fulani, we inferred admixture dated to 670 to 1190 CE between a source related to Morocco Berbers and a source related to populations in the Fulani’s assumed homeland of The Gambia and Senegal using MOSAIC and MALDER. FastGLOBETROTTER inferred similar sources of admixture but with multiple dates of 1800 CE (1510 to 1850 CE) and 680 BCE (2090 BCE to 130 CE). Furthermore, both fastGLOBETROTTER and MOSAIC inferred these Cameroonian Fulani to best represent one of the sources of an admixture event dated to 1650 to 1800 CE in a group of Sudanese (“Sudanese clustering near Fulani” group in Fig. 5, fig. S13, and data S7), with the other source inferred to be northern Cameroon–like.

### Inferred admixture from Arab-like sources in Cameroon and Sudan spanning 2000 years

Four supergroups from Cameroon and Sudan show evidence of admixture from Arabian/Levantine-related sources (red events in Fig. 5A). In Arabs from Cameroon and in clusters of Sudanese living along the Nile, pulses of admixture from a Saudi Arabian–like source are dated to ~1340 to 1720 CE. In some Nile-based Sudanese, fastGLOBETROTTER infers an additional older admixture event dated to 640 CE (160 to 800 CE), also from a source related to present-day Saudi Arabians and consistent with continuous gene flow from the Arabian Peninsula over a long period. In contrast, inferred Arabic-related admixture in the Beni-Amer involves an African source more closely related to Somalis and dates to 680 to 1130 CE with MOSAIC and fastGLOBETROTTER and 170 BCE to 580 CE in MALDER. No Arabic-related admixture is inferred in the two clusters from the South Kordofan region of Sudan using MOSAIC and fastGLOBETROTTER. MALDER infers old admixture in both these supergroups, although with large confidence intervals (4590 BCE to 990 CE).

### Admixture in southern Cameroonians, Ghanaians, Nigerians, and Congolese correlates with the initial expansion of Bantu-speaking peoples

FastGLOBETROTTER inferred similar admixture events in the history of Narrow Bantu speakers and Grassfields speakers from Cameroon, Nigerians, and Ghanaians, between sources related to coastal West Africans and speakers of Bantu languages. MALDER also inferred admixture between two African populations in Northern Bantoid speakers and Grassfields speakers from Cameroon with confidence intervals overlapping these events, while MOSAIC inferred no admixture in these supergroups (Fig. 5A and fig. S16). FastGLOBETROTTER’s inferred point estimate dates are more recent for the more western supergroups, Ghana and Nigeria West (450 and 200 BCE, respectively) and older for Nigeria East, Cameroon Grassfields, and Cameroon Narrow Bantu (1420, 980, and 820 BCE, respectively), although 95% confidence intervals overlap for all dates (see fig. S17 for coancestry curves).

In Congolese, fastGLOBETROTTER inferred evidence of multiple admixture pulses, at 560 BCE (1790 to 320 BCE) and 1260 CE (950 to 1710 CE), each between sources related to Bantu language–speaking peoples and rainforest hunter-gatherers (Biaka-like). Both MALDER and MOSAIC replicate the more recent event, with these approaches not capable of detecting multiple admixture events involving the same sources (*25*, *26*).

### Admixture in Bantu-speaking peoples consistent with multiple waves of expansion and a “late split” route

**Fig. 7.**
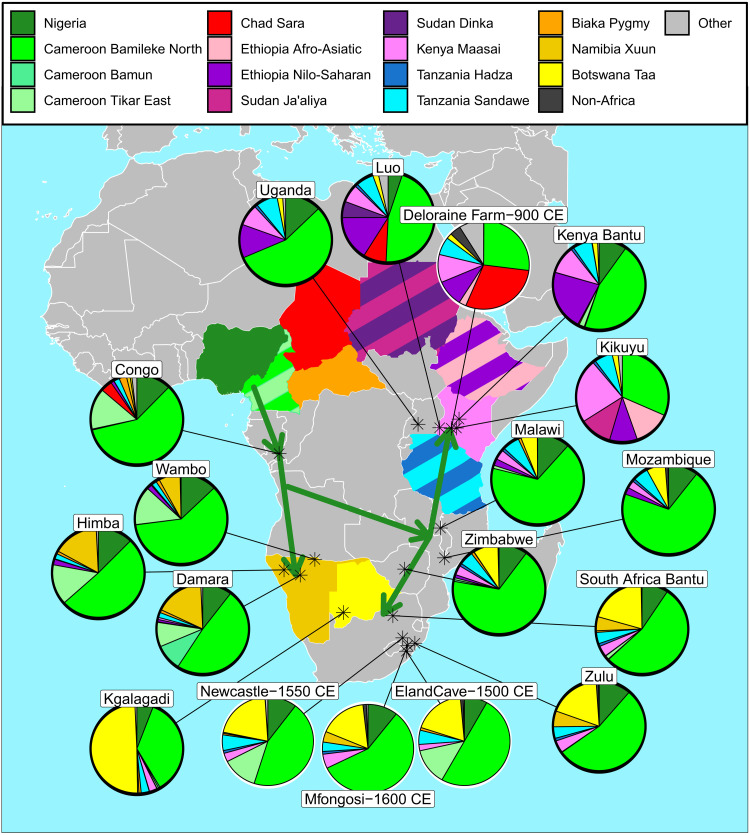
Cameroonian-related genetic variation in Africa-wide populations as a result of the expansions of Bantu-speaking peoples. For 4 ancient individuals from South Africa and Kenya (enclosed by a white border) and 14 present-day populations, pies give the SOURCEFIND-inferred proportions that best describe the genetic variation patterns in each group as a mixture of those in the surrogate populations. In all cases, the highest proportion related to Cameroonian populations was specifically matched to the Bamileke. Arrows depict the hypothesized route of the expansion of Bantu-speaking peoples based on these data (see below). Ethiopian Afro-Asiatic–speaking clusters, Nilo-Saharan–speaking clusters, and non-African clusters have been grouped for ease of visualization (see data S8).

Linguistic evidence indicates that the expansion of Bantu-speaking peoples originated in the Cameroon/Nigeria border region (*40*, *51*), suggesting that genomes from this region are likely good proxies for ancestry related to Bantu language speakers in other populations. To investigate this, we ran SOURCEFIND (*50*) on 14 present-day populations and 4 ancient individuals previously reported to have genetic variation related to present-day Bantu language speakers (Fig. 7 and Materials and Methods) (*7*, *10*, *13*, *15*). We used 270 other sampled populations with ≥4 individuals in the dataset as potential surrogates to admixing sources, including the 8 Bantu language–speaking Cameroonian groups and 262 non–Bantu language–speaking groups (data S4). These non–Bantu languagespeakers contain Southern Bantoid speakers from Cameroon and Nigeria, the language family from which Bantu languages originated (*52*). In all 18 present-day and ancient groups, SOURCEFIND inferred the non-Bantu Southern Bantoid language–speaking Bamileke (specifically the Bamileke North cluster) from western Cameroon to best reflect genetic variation patterns related to Bantu language speakers (Fig. 7).

As before, we used three methods, fastGLOBETROTTER (*27*), MALDER (*26*), and MOSAIC (*25*) to date admixture between the Cameroon-like source and local source in the 13 present-day populations (excluding the Republic of the Congo that was analyzed earlier) and 4 ancient individuals previously reported to have genetic variation related to present-day Bantu language speakers (fig. S18 and data S8). Admixture between a West African-like source and a source related to a geographically proximate population was inferred in ≥2 methods for 11 present-day groups, 10 of which had overlapping confidence intervals for inferred admixture dates in at least two methods. In many of these events, MOSAIC inferred a date more recent than that inferred by MALDER and fastGLOBETROTTER, as has previously been reported (*7*). Multiple waves of admixture were inferred by fastGLOBETROTTER in three groups, with one event inferred to be older than 0 CE in each case. Of the more recent admixture events and those where one date was inferred, date point estimates ranged from 170 BCE to 1630 CE. Consistent with SOURCEFIND inference, fastGLOBETROTTER and MOSAIC reported the Bamileke to be the best representative source for Bantu speaking–related admixing source in the majority of groups.

We next investigated the route of the expansion of Bantu-speaking peoples. Mimicking previous approaches that tested for evidence of a late split of Bantu language speakers into Southern and Eastern branches during the expansion (*5*, *15*, *23*, *30*), we reran fastGLOBETROTTER using (i) only Cameroonian and Congolese Bantu language speakers or (ii) all Bantu language speakers as potential surrogates for Bantu-speaking-related ancestry (fig. S19). Congolese groups were favored over Cameroonians as surrogates in all Bantu language–speaking target populations in analysis (i), and Bantu language–speaking groups from Malawi and Mozambique were favored as surrogates in Southern and Eastern Bantu language–speaking groups in analysis (ii) (fig. S19). These observations are consistent with previous results supporting a late split model, whereby Bantu language–speaking peoples initially migrated south through the Republic of the Congo [and possibly further south to Angola (*23*)]. This was followed by an eastward migration, potentially as far as Malawi and Mozambique, before the split into Eastern and Southern branches.

Archaeological evidence has recently provided support for a population collapse at ~600 CE in the Congo basin, followed by a secondary spread event roughly 800 years later (*53*, *54*). To assess genetic signatures of this, we used GONE (*55*) and IBDNe (*56*) to infer recent changes in effective population size (*N*_e_) in Republic of the Congo. We found evidence of a small population expansion beginning roughly 60 generations ago or earlier using both methods and evidence of a recent (20 generations ago) decrease, followed by continued expansion using IBDNe. There was no indication of an older population collapse (fig. S20). However, we assessed the power to identify such a collapse by applying GONE and IBDNe to several simulations that mimic the proposed changes in effective population size from archaeological data (see Materials and Methods, fig. S21, and text S4). Our results show that an “expansion-bottleneck-expansion” scenario would be very difficult to distinguish from a single recent expansion using our genotype array data and current methods, and therefore, our data do not appear well suited to test this hypothesis using these techniques. In addition, given admixture can cause an increased effective population size inference (*55*), it likely will be difficult to disentangle inferred admixture in the populations from the Republic of the Congo from population size changes.

## DISCUSSION

Here, we present analyses of newly reported genome-wide autosomal variation data from 1387 individuals, the majority sampled from Cameroon, Republic of the Congo, Ghana, Nigeria, and Sudan. These data can be used to explore an array of hypotheses, such as those based on available anthropological and archaeological records. Below, we leverage genetic data in relation to each of the questions listed in Introduction. While we relate some of these genetic signatures to historical events, we note that it is not possible to determine the exact cause of historical intermixing. This is especially true when the confidence intervals around an inferred date are large. Instead, we provide possible explanations based on overlapping dates, populations, and geographic regions.

### Genetics is correlated with geography, self-reported ethnicity, and language within each of Cameroon, Republic of the Congo, Ghana, Nigeria, and Sudan

Leveraging a densely sampled dataset of West and Central Africans and Sudanese, we infer a previously underappreciated degree of fine-scale genetic structure in African populations. We observe the clustering of individuals by country (Fig. 2 and fig. S8) and by geography and/or ethnicity within Cameroon, Ghana, and Sudan (Fig. 4). Genetics often correlates with major linguistic phyla (e.g., Niger-Congo versus Afro-Asiatic in Cameroon and Nilo-Saharan versus Afro-Asiatic in Sudan) and sometimes with smaller linguistics groupings, for example, among different Bantoid language speakers in Cameroon (fig. S9) (*1*). Some of these genetic differences, such as those within the Congo basin, have previously been difficult to capture (*5*). The genetic structure that we infer is often more detectable when using haplotype-based techniques (figs. S4 and S5), supporting recent work indicating how haplotypes can better describe fine-scale structure (*8*, *9*). This may have implications when properly adjusting for stratification in large-scale GWAS (*31*).

Perhaps unexpectedly, ethnic groups in southeastern Nigeria and western Cameroon cluster largely by country of origin, despite the border only existing since 1913 CE and with little consideration given to the distribution of ethnic groups during its creation (*57*, *58*). This suggests that our sampled groups were isolated from one another before the formation of this border, perhaps due to older structure between subgroups now present on either side. This structure could result from the topographical barriers that the border tends to follow (*57*). However, none of the sampled ethnic groups included individuals from both countries, and so we note that there may be cases, not well represented by the sample collection here, where groups on either side of the border are more genetically similar.

In notable contrast to these observed associations between genetics, ethnicity, and geography, genetic variation patterns among Sudanese belonging to Arabic and Nubian ethnic groups sampled along the Nile using a transect approach show almost no correspondence with ethnicity (fig. S8), and only a subtle isolation by distance relationship (fig. S11). In contrast, a previous study that sampled each Sudanese population from a single location found Arabic and Nubian groups to be genetically distinguishable (*16*). This is consistent with the Nile acting to promote intermixing among groups in Sudan, e.g., as a corridor of gene flow, as has previously been suggested using mitochondrial DNA data (*59*). Almost all Arabic, Beja, and Nubian individuals fall into two genetic clusters whose main difference is their proportion of genetic variation patterns inferred to be recently related to Arabian groups (48% versus 12%) (Fig. 5C, Nile1 versus Nile2), with less such inferred Arabian-related ancestry in Beja and Nubian individuals, on average.

### Does genetic structure correlate with historical polities in the Grassfields of Cameroon?

Figure 4D demonstrates fine-scale structure in southern and western Cameroon, including in the Grassfields. The Grassfields region of Cameroon (broadly both the Northwest and West regions) is home to ethnic groups with a range of histories and polities, from those who at the end of the 19th century had large, unified kingdoms (known as fondoms) such as the Bamun (*60*) to smaller ethnic groups consisting of only a few villages (e.g., the Aghem; text S2) (*61*, *62*). We inferred notable fine-scale structure among Grassfields individuals, with most ethnic groups constituting their own genetic cluster, even those where sampled individuals reside within 20 km of each other (Fig. 4D). We believe that this structure is a result of isolation between groups rather than differential admixture, because we infer that all of these groups are similarly genetically related to non-Grassfields populations (fig. S15). We caution that we may have limited power to detect genuine admixture differences among them if we have not included good enough proxies to (unknown) contributing ancestral sources, although we have included 154 sampled African groups as surrogates.

We found two key exceptions where ethnic group does not correspond with genetic cluster. First, the Nso’ and Wimbum cluster together despite speaking different branches of Grassfields languages and living 70 km apart. The Nso’ formed a large, unified kingdom in precolonial times and had influence over nearby ethnic groups, possibly facilitating gene flow (*63*). Another ethnic group, the Noni, live within 20 km of the Nso’ but do not cluster with them. Although the Noni were governed at times by the Nso’ Kingdom, they maintained a separate identity and attempted to establish independence in both colonial and postcolonial times, which may have limited gene flow between the two groups (*63*, *64*). The second exception is in those who self-identify as Tikar, who fall into two separate genetic clusters. In particular, all self-identified Tikar who report speaking Tikar and live in the Adamawa region cluster among other Northern Bantoid speakers. In contrast, self-identified Tikar who do not report speaking Tikar and live in the Grassfields cluster with other Grassfields ethnic groups (*35*).

Of the sampled Grassfields ethnic groups, the Bamun and the Bamileke have the lowest inferred within-group IBD sharing (fig. S14). The Kingdom of Bamun was reported to be the largest in the Grassfields and known for both fighting and trading with neighboring ethnic groups (*60*, *65*). These interactions may have acted to reduce genetic isolation/endogamy in the Bamun. In general, these results suggest that different political structures in the Grassfields region of Cameroon do not correspond with similar genetic signatures. It is also important to consider colonial history when interpreting patterns of genetic variation in the Grassfields. For example, the Bamileke label was given to several smaller fondoms by the Germans during the colonial period (*62*), and this may explain their relatively high genetic diversity compared to nearby ethnic groups that have not had such broad colonial labels imposed upon them (figs. S8 and S14).

### Do we detect structure between Kordofanian speakers and Nilo-Saharan speakers in southern Sudan?

In contrast to the lack of genetic structure observed among Sudanese sampled along the Nile, we inferred very fine-scale structure among individuals sampled in the Nuba mountains of South Kordofan, which correlates with ethnolinguistic group (Fig. 4C). This region has been described as a historic refuge due to its inaccessible nature (*66*). Ethnologue (*34*) places the South Kordofan languages into two macrophyla; Niger-Congo (Kordofanian) and Nilo-Saharan, although with controversy over classifications and several languages often categorized as isolates (*33*, *36*). We inferred groups classified as Kordofanian to be genetically distinct from groups classified as Nilo-Saharan, with the latter showing a greater genetic affinity to sampled Nilo-Saharan speakers from Ethiopia (Fig. 2 and fig. S9B). These differences remain after mitigating recent endogamy effects (text S3), indicating perhaps some ancient structure between Kordofanian and Nilo-Saharan speakers correlating with speaking languages from different macrophyla. We also infer a relatively low correlation between genetics and geography in this region (fig. S5H), although this is likely a result of relatively isolated groups (e.g., Acheron) distorting the PCs. Replicating previous reports (*16*), we inferred no evidence of Arabian-related admixture in South Kordofan after the Arabic expansion into Sudan (see below), again consistent with the mountains’ role as a refuge (*67*).

### Can we date admixture as a result of the Arabic expansion into Africa?

We inferred multiple waves of admixture in Sudanese related to Arabian-like sources. In contrast to other sampled Sudanese ethnic groups, the Beni-Amer, a coastal Beja ethnic group, exhibit a greater inferred proportion of genetic variation related to Ethiopian Afro-Asiatic groups (Fig. 5C) and an older wave of admixture from a source related to Saudi Arabians and Yemenis dated to the first millennium CE. The Kingdom of Aksum extended across northern Ethiopia, coastal Sudan, and Yemen during this period and was known to trade with the Arabian Peninsula, providing a potential explanation for the inferred gene flow, although other interactions unrelated to the empire could also explain this signal (text S2) (*68*). This admixture event has been reported previously, along with additional non-African admixture dated to an earlier period in the Beni-Amer (*16*), which we may not infer here because of masking by more recent signals (*69*).

We inferred admixture in two other Sudanese clusters that primarily contain Nile inhabitants, dated to 1340 to 1730 CE between sources related to (i) present-day Arabians and (ii) East African Nilo-Saharan speakers. The inferred admixture date and sources, consistent with previous findings (*16*, *21*), may reflect the collapse of the Kingdom of Makuria over this period, which allowed Arabic groups to expand down the Nile into Sudan (*70*). In one of these clusters, we replicate previous reports (*16*) of an older pulse of admixture between similar sources, dated to 640 CE (160 to 800 CE), indicating a possible wave of migration into Sudan that predates or coincides with the seventh century Arabic expansion (*67*). Last, we inferred Arab-like admixture in Arabs from the Far North region of Cameroon dated to the 16th century, overlapping with reported migrations of Arabic groups into the Lake Chad area from the mid-14th century onward (*37*, *70*).

### What was the impact of the Kanem-Bornu empire on the genetics of the northern Cameroonian populations associated with it?

In both the Chadic-speaking Kotoko and the Nilo-Saharan–speaking Kanuri, we inferred admixture events, dated to 960 to 1690 CE in the former and 820 to 1760 CE in the latter, involving three distinct sources related to present-day East Africans, West/Bantu language–speaking southern Africans, and North Africans/Levantines/Arabians, respectively (Fig. 6). The inferred dates overlap the Kanem-Bornu empire, which was present in northern Cameroon at the time. The empire was based in Kanem, east of Lake Chad, from 700 CE. In the late 1300s, the center of the empire shifted to Borno in northeastern Nigeria and continued there until the late 1800s. It is during this later period that local Chadic-speaking populations, culturally and linguistically related the Kotoko, were assimilated into the Nilo-Saharan–speaking empire and the Kanuri emerged as an ethnic group (*38*). This may explain why two approaches infer admixture dates in the Kotoko during the later stage of the empire (Fig. 6). In the Kanuri, MOSAIC infers multiple admixture events within the date span of the empire, one during the early stage or the empire and one during the later stage, perhaps reflecting more continuous mixture over a longer period. The empire is known for its trade links between North, West, and East Africa (*71*), which plausibly facilitated the intermixing of peoples from these regions. However, we note that our confidence intervals span a long time period and gene flow into northern Cameroon at this time may be associated with long-distance interactions unrelated to the empire.

### Can we date admixture in the history of the Fulani in northern Cameroon?

We inferred similar admixture sources in Fulani sampled from the Far North and Adamawa regions of Cameroon as have been reported in studies of Fulani from other countries (*15*, *20*, *22*), with one source related to Morocco Berbers contributing ~12% and the remaining DNA contributed by a source related to Gambians and Senegalese (Fig. 5C). Admixture between sources related to Morocco Berbers and Gambians was dated to 670 to 1190 CE using MALDER and MOSAIC in these Cameroonian Fulani, which is more recent than some previous estimates of admixture dated to ~200 CE in Fulani sampled from Gambia, Burkina Faso, Niger, and Chad (*15*, *20*, *22*). FastGLOBETROTTER inferred multiple dates of admixture, with the older event between similar sources as above dated to 700 BCE, although with very large confidence intervals. It is possible that the very recent mixing that fastGLOBETROTTER infers pushed back its date inference for the older event, as has been demonstrated before (*24*). Trans-Saharan trade and migration routes have linked North and West Africa for thousands of years and may have facilitated the inferred gene flow (*15*, *72*). We also infer a larger amount of IBD sharing within the Fulani cluster (fig. S14), potentially as a result of the ethnic group’s historical endogamous practices (*20*).

We inferred admixture between a Fulani-like source and a northern Cameroonian-like source dated to 1650 to 1800 CE in several Sudanese individuals (Sudanese clustering near Fulani in Figs. 3 and 5 and data S7). Since the non-Fulani source is more closely related to Cameroonians than Sudanese, it indicates that the admixture likely took place further west than Sudan. The inferred confidence interval overlaps with a historically attested period of increased interaction between Fulani, Hausa, and other Chadic-speaking populations in northern Nigeria and Cameroon, which culminated in the Fulani jihad of Usman dan Fodio, and the establishment and expansion of the multiethnic Sokoto Caliphate (*73*). Some descendants of this mixture may have subsequently migrated to Sudan, although there are several other possible explanations, including admixture within Sudan between groups who had migrated east at an earlier point.

### Which Afro-Asiatic–speaking population is most closely related genetically to Afro-Asiatic Chadic speakers in northern Cameroon?

In a supergroup containing 11 of 14 Afro-Asiatic Chadic-speaking ethnic groups from northern Cameroon (Cameroon North supergroup; Fig. 3), we inferred admixture dated to 710 CE (10 BCE to 840 CE) between multiple sources represented by present-day coastal West Africans, Bantu-speaking groups, and Nilo-Saharan speakers from Ethiopia and Chad (Fig. 5). Since the admixture involves multiple ancestral sources, the direction of the migration event is difficult to deduce. While too recent to be related to the initial migration of Chadic speakers into Cameroon around 6000 to 2000 BCE (*21*, *39*), similar dates and sources of admixture have been reported for the Niger-Congo–speaking Berom of northern Nigeria (*5*). Together, these results suggest a little characterized mixture event in northern Cameroon and northern Nigeria during the first millennium that involved sources genetically related to East Africans and West Africans. The period corresponds with archaeological evidence for a marked increase in the presence of exotic grave goods and, thus, trade with external sources in the region (*74*). Consistent with previous reports, in Chadic speakers, we find evidence of large amounts of genetic variation recently related to Nilo-Saharan speakers from Cameroon and Chad, making the closest Afro-Asiatic–speaking relatives of the former language family difficult to discern. Sampled Nilo-Saharan and Chadic speakers within northern Cameroon appear genetically indistinguishable using our approaches (fig. S9A).

### What was the impact of the expansion of Bantu-speaking peoples on modern-day African populations?

Given the dense sampling of individuals from the “cradle of the Bantu languages” in and around the Nigeria/Cameroon border region (*40*, *51*), we explored which sampled Cameroonian and Nigerian groups were most representative of ancestry related to Bantu language–speaking groups across Africa. There is controversy over the early splits in the Bantu language phylum, such that proto-Bantu is likely to be paraphyletic within the wider Southern Bantoid phylum and, thus, the distinction between Bantu and non-Bantu Southern Bantoid is debated in some cases (*75*). Consistent with this, approaches using SOURCEFIND, fastGLOBETROTTER, and MOSAIC inferred that Bantu language–speaking components in all populations are most closely related to the non-Bantu Southern Bantoid–speaking Bamileke, even compared to Bantu language–speaking populations from Cameroon (Fig. 7). However, this may be a result of the low within-group IBD-sharing in the Bamileke (fig. S14) consistent with less recent endogamy, which potentially makes the population a better ancestry surrogate. We emphasize that this result does not imply that the Bamileke were the source of the expansion, as it is likely that the genetic structure of the region was not the same ~4000 years ago at the beginning of the expansion of the Bantu languages as it is now.

In Bantu language–speaking groups where a single date of admixture is inferred, including in three ancient (530 to 310 years B.P.) individuals from South Africa, events involved a Bantu language–speaking-like source, and a local source. Date point estimates ranged from 170 BCE to 1630 CE (fig. S18 and data S7), in line with those inferred in previous studies (*4*, *5*, *7*, *15*, *23*, *30*). Our inferred dates for when Bantu language speakers admixed with local populations are often more recent than when archaeological and linguistic evidence suggests that they first arrived in a region (*76*). This could be explained by long isolation of Bantu language speakers after their initial migration and/or multiple “spread over spread” migrations along similar routes and involving similar sources obscuring the original admixture event (*53*, *54*, *77*). Consistent with the latter, in new data from Bantu language speakers in the Republic of the Congo, we find evidence of multiple admixture events, dated to 560 BCE and 1260 CE, that both involve sources related to present-day Bantu language speakers and rainforest hunter-gatherers (Fig. 5, A and B). The older date is consistent with the initial stage of the expansion of Bantu language–speaking peoples into the Congo rainforest at ~800 BCE (*78*) and matches previous admixture inference in groups from the Democratic Republic of the Congo (*5*). Admixture similar to the inferred recent event has also been reported previously in groups from Gabon and Angola and could represent a secondary “spread” event (*23*, *53*). Consistent with the “spread over spread” theory, we also find evidence of multiple admixture dates between a Bantu language–speaking source and a local source in the South African Zulu, Kenyan Bantu language speakers, and Ugandan Bantu language speakers, with the older date overlapping the period Bantu language speakers first migrated into these regions (fig. S18). These three clusters have the largest sample sizes among Bantu-speaking groups, increasing our power to detect these older events.

In Ghanaians, Nigerians, and the three supergroups from southern Cameroon, fastGLOBETROTTER and, in some cases, MALDER inferred admixture events between West African-like and Bantu speaking–like sources, with large confidence intervals, but point estimates indicating a date earlier than 200 BCE (figs. S16 and S17). Although these events were not replicated using all methods, similar admixture signals with overlapping confidence intervals have previously been reported for Ghanaians and Nigerian Yoruba (*28*, *79*). These results may reflect mixing between neighboring groups in West Africa around the time of the early stages of the expansion of Bantu language–speaking peoples. There is mounting evidence that this early migration stage was a response to climate-induced savannah expansion in central Cameroon (4000 to 3500 years B.P.) and later in the core of the Central African forest block at 2500 years B.P. (*78*). Although paleoclimate data from further west are limited for this time, an abrupt drying spell is evidenced by the sudden drop in the level of Lake Bosumtwi, Ghana, at ~3200 years B.P. (*80*, *81*). Thus, climate change may have similarly instigated a period of increased migration and mixing between groups at the periphery of the rainforest extending to Ghana. Future studies, with more West African populations, including those from Togo, Benin, and further west could help to examine this signal further.

Overall, our results highlight the extra insight that can be gained from interpreting genetics within the context of archaeological, linguistic, and historical data. Examples of this include understanding the potential historical influences on genetic structure in the Grassfields of Cameroon and investigating the timings of the Arabic expansion into Africa. Of course, each group has its own unique history; we hope that the novel data resource published here will enable future hypothesis-driven analyses of the >150 ethnolinguistic groups.

We have provided evidence for both isolation effects and extensive mixture between groups. This genetic heterogeneity reinforces how the current sparse sampling of groups from these regions misses large swathes of the obtainable genetic variation and ancestral information, even among geographically proximate populations. Future GWAS that include people with African-related ancestry must consider how these populations may have very different frequencies of pharmacologically relevant alleles (*4*, *5*, *7*, *82*), necessitating dense sampling that considers geography, linguistics, and ethnicity.

## MATERIALS AND METHODS

### Samples

DNA samples from 1510 individuals from Cameroon, Republic of the Congo, Ghana, Mozambique, Nigeria, South Africa, Sudan, and Zimbabwe, 1387 for whom new autosomal genetic variation data are reported here following quality control, were collected from 1997 to 2006 on several field trips, the majority organized by Neil Bradman. All study participants whose genetic variation data are newly reported in this study gave informed consent to use their data to investigate the genetic histories of human populations and to describe patterns of genetic variation within and among human populations. Local permissions were obtained for sample collections from Cameroon (approval from the Ministry of Higher Education and Scientific Research, permits 0188/MINREST/B00/D00/D10/ D12 and 317/MINREST/B00/D00/D10 and University of Yaoundé I), Republic of The Congo (Ministère De La Santé Et De La Population), and Sudan (approval from the Secretary General of the Sudan Medical Council). For the other samples local permissions were obtained through consultation with heads of villages and communities, since no official national or local procedures existed at the time of collection. Typing of the sample collections was approved by the U.K. ethics committee London Bentham REC (formally the Joint UCL/UCLH Committees on the Ethics of Human Research: Committee A and Alpha, REC reference number 99/0196, Chief Investigator MGT). The analyses reported in this manuscript were approved by UCL REC (project IDs: 5188/001 and 5188/002).

DNA was collected in the form of buccal swab samples. All donors were anonymous and over the age of 18. For each sampled individual excluding individuals from Mozambique and Zimbabwe, we recorded self-reported information about the individual’s, their parents, their paternal grandfather’s birthplace, and their maternal grandmother’s birthplace, ethnic group, and first and second language (data S1 and fig. S1). Two of the individuals sampled in South Africa had a birthplace in Senegal. All newly reported DNA samples were genotyped using the Affymetrix Human Origins SNP array, which targets 627,421 SNPs. We merged these data with previously published Human Origins or genome-wide datasets, totaling 4267 individuals, including both Africans and non-Africans (Table 2, fig. S2, and data S2) (*4*, *6*, *8*, *11*, *12*, *41*–*45*). We also merged 20 high-coverage (>1× average coverage) ancient individuals from Africa (data S2) (*10*, *12*, *13*). The newly reported samples could also be integrated with other recently released data from West and East Africa (*16*, *23*); we exclude these datasets here due to reduced SNP overlap among arrays (<52,000 SNPs) likely reducing power when using haplotype-based approaches.

**Table 2.** Datasets merged in with the newly reported samples (fig. S2 for map and data S2).

Reference	Number of samples
Byrska-Bishop *et al.* (*43*)	240
Fan *et al.* (*6*)	47
Gurdasani *et al.* (*4*)	356
Lazaridis *et al.* (*41*)	1537
Lipson *et al.* (*12*)	58
López *et al.* (*45*)	69
López *et al.* (*8*)	1143
Malaria Genomic Epidemiology Network (*44*)	359
Prendergast *et al.* (*13*)	15
Schlebusch *et al.* (*10*)	4
Skoglund *et al.* (*11*)	34
Zheng-Bradley *et al.* (*42*)	4

### Data processing

We downloaded the ancient samples from the European Nucleotide Archive website (www.ebi.ac.uk/ena) and used PicardTools to check for correct format and metadata. We used ATLAS with the “pmd” flag to estimate postmortem damage. We recalibrated each BAM file by running ATLAS with “recal,” using the ultra-conserved non-coding elements (UCNE) from UCNEbase (https://ccg.epfl.ch/UCNEbase/) (Table 3). Maximum likelihood genotype calls and phred-scaled genotype likelihood scores were generated for each position using ATLAS with “call” (*83*). To ensure that strands were consistent with 1000 Genomes across present-day and ancient datasets, we used Conform-GT (https://faculty.washington.edu/browning/conform-gt.html). We merged the data, re-estimated genotypes, and imputed missingness using Beagle 4.1 (*84*) with “modelscale = 2.” We used vcf2gprobs, gprobsmetrics, and filterlines (https://faculty.washington.edu/browning/beagle_utilities/utilities.html) to remove SNPs with an imputation accuracy of less than 0.98. Last, we phased all samples using shapeit4 (*85*) with “--pbwt-depth 16” and their provided genetic maps.

**Table 3.** Software used in the analysis, with a brief description and nondefault parameters used.

Software	Description	Parameters	Reference
ATLAS	Genotype calling for aDNA samples	pmd, recal	Link *et al.* (*83*)
Plink 1.9	Inferring relatedness, to remove closely related individuals, plus other quality control (e.g., pruning SNPs)		Chang *et al.* (*86*)
Beagle	Merging data, re-estimating genotypes, and imputing missing sites		Browning and Browning (*84*).
Shapeit4	Phasing		Delaneau *et al.* (*85*).
smartPCA	Calculating PCs	lsqproject, newshrink	Patterson *et al.* (*46*).
ADMIXTURE	Clustering each individual into 1 or more of *K* “ancestry components,” illustrating patterns of genetic variation in the dataset		Alexander and Lange (*48*)
ChromoPainter	Along the genome, inferring which haploids in set of “donor” individuals share ancestors most recently with a target individual	Values specified below	Lawson *et al.* (*47*).
fineSTRUCTURE	Clustering individuals into genetically similar groups and estimating a dendrogram of relatedness between clusters	Clustering: -X ▬Y -y 3000000 -z 10000	Lawson *et al.* (*47*).
		Tree-building: -x 100000 -m T -t 1000000 -T 1 -k 2	
Hap-IBD	Inferring position and length of IBD segments >2 cM between pairs of individuals	merge-ibd-gaps gap = 0.6 discord = 1	Zhou *et al.* (*49*).
SOURCEFIND	Inferring genetic variation patterns of a target group’s individuals as a mixture of those of other sampled populations, reflecting recent ancestry sharing	self.copy.ind: 0	Chacón-Duque *et al.* (*50*)
		num.surrogates: 8	
		exp.num.surrogates: 4	
MALDER	Inferring and dating admixture events in a population		Loh *et al.* (*26*)
fastGLOBETROTTER	Inferring and dating admixture events in a population		Wangkumhang *et al.* (*27*)
MOSAIC	Inferring and dating admixture events in a population		Salter-Townshend and Myers (*25*)
IBDNe	Inferring historical effective population size	filtersamples = true	Browning and Browning (*56*)
GONE	Inferring historical effective population size	PHASE = 2	Santiago *et al.* (*55*)
msprime	Simulating ancestral histories with given changes in effective population size	See text S4	Baumdicker *et al.* (*90*)

We identified putatively related individuals using PLINK v1.9 (*86*) by first pruning for LD using “--indep-pairwise 50 10 0.1” and then using “--genome” to infer pairwise PI_HAT values. Following López *et al.* (*8*), we identified individuals with outlying PI_HAT values relative to other members of the same group label instead of using the same fixed PI_HAT threshold value for all populations. This prevented us from removing too many individuals from populations with relatively low diversity. As in López *et al.* (*8*), we found all pairings of individuals from populations (*i,k*) that had PI_HAT > 0.15 and PI_HAT > min(*X_i_* + 3*max{0.02,*S_i_}*, *Y_i_* + 3*max{0.02,*D_i_*}, *X_k_* + 3*max{0.02,*S_k_*}, *Y_k_* + 3*max{0.02,*D_k_*}), where {*X_i_*, *Y_i_*, *S_i_*, *D_i_*} are the {mean, median, standard deviation, median-absolute-deviation}, respectively, of pairwise PI_HAT values among individuals from population *i*. For populations with ≤2 sampled individuals, the standard deviation and median-absolute-deviations are undefined or 0; therefore, in these cases, we added to the list any pairings with PI_HAT > 0.15 that contained ≥1 person from that population. Using a stepwise greedy approach, we then selected individuals from this list that were in the most pairs to be excluded from further analysis, continuing until at least one individual had been removed from every pair. This removed 515 of our individuals, and all remaining PI_HAT values were below 0.24. We then applied a PI_HAT threshold of 0.18 to any pairs of African individuals, leading to three more individuals being removed. Fifty-three of the individuals in our newly published samples were duplicates, sometimes with inconsistent ethnolinguistic and/or birthplace information. In these cases, we randomly chose one individual from the pair (removing 26 individuals) but excluded the individual from any later analyses involving ethnic group or birthplace information. Overall, quality control steps resulted in the removal of 544 individuals (421 of which were from the previously published dataset). After quality controls, we were left with a dataset of 5253 individuals, 1387 of which are newly published here, typed at 510,615 autosomal SNPs.

For individuals from Mozambique and Zimbabwe, no information about birthplace was available. Of all other newly published individuals, most reported having grandparents from the same ethnic group. Exceptions are 23 individuals who reported grandparents of different ethnic groups and 58 individuals with missing or unknown information for at least one grandparent, although all but one of these 58 individuals had matching reported ethnic group information for all other relatives recorded. In addition, samples were selected such that, in 1159 of the 1445 samples with background information (including previously published samples), maternal grandmother’s and paternal grandfather’s birthplace matched. These two steps should help reduce effects of recent movement or ethnic group-switching, analogous to Leslie *et al.* (*29*). The 23 samples with grandparents of different ethnic groups were removed from any analyses comparing genetics with self-reported ethnic group. If an individual’s maternal grandmother and paternal grandfather had a different birthplace, the individuals’ geographic location was calculated as the mean of the grandparent’s birthplaces, using the Haversine function from the R package geosphere. If grandparent birthplace information was missing, then parental information was used instead. When there was no available information about relative’s birthplace or the individual’s grandparents lived further than 150 km apart, the individual’s own birthplace was used, and these samples were excluded from later analyses involving birthplace (114 individuals excluded, see below). Individuals typed for this paper were classified into 166 ethnic groups based on self-reported information. Data S1 contains newly reported sampled individuals whose genetic data passed quality controls, and Fig. 1 shows the geographic locations of each sample from Cameroon, Republic of Congo, Ghana, Nigeria, and Sudan, grouped by location.

Languages were classified using Glottolog (*33*) for all countries except Sudan, where Ethnologue (*34*) was used. This is because we inferred genetic differentiation between languages from the South Kordofan region in Sudan classified as Nilo-Saharan and Kordofanian in Ethnologue (see below). This level of classification is not included in Glottolog, as it is more disputed. For our genetic comparisons of people from different language classifications (fig. S9), the level of language classification was chosen for each country such that the largest amount of diversity was captured while maintaining a reasonable sample size (≥5 individuals) and ensuring most language classifications contained multiple ethnic groups. This was impossible in some cases in Sudan and Nigeria, where there were several linguistic groups that only contained one ethnic group.

### *F*_st_, ADMIXTURE, and smartPCA

We first filtered sites to remove those in LD using PLINK v1.9 (*86*) and the tag “--indep-pairwise 50 10 0.1” as above. We then calculated Weir and Cockerham weighted *F*_st_ between populations or countries using PLINK v2 and the --fst tag. We used ADMIXTURE v1.3.0 (*48*) to cluster newly genotyped individuals, including a range of reference populations. We used *K* with values from two to nine and default parameters. The lowest cv error was at *K* = 7. Last, we calculated PCAs using smartPCA (*46*) and the options lsqproject, newshrink, and no outlier removal iterations.

### Plotting maps

We used the ggmap package with the get_map function in R to plot the maps in Figs. 1, 4, and 6, with the source set as “google” and the maptype as “terrain.” All other maps were plotted using map_data from the ggplot package.

### Procrustes analysis

Procrustes analyses were carried out using the vegan package in R, with the options truemean = TRUE and scale = TRUE (*87*). Correlation and sum of squares was calculated with the protest function and the options scores = “sites” and permutations = 999.

### Applying chromosome painting to all groups

We used ChromoPainter (*47*) to investigate patterns of haplotype sharing in our dataset. We formed (“painted”) the two phased haploids of each (target) individual in the dataset as a mosaic of DNA segments matched to other sampled individuals. Following previous studies (*8*, *9*), we calculated the switch parameter, Ne (“-n”) and the mutation parameter, θ (“-M”) using 10 iterations (“-i 10”) of the ChromoPainter Expectation-Maximization (*E*-*M*) algorithm, painting only chromosomes 1, 4, 15, and 22 of every 10 of the 5253 individuals for computational efficiency. We ran two separate ChromoPainter analysis, “Internal” and “External,” described in text S3. Unless otherwise noted, results reported here use the Internal analysis, which painted each target individual against all other sampled individuals (using the -a parameter).

We then ran PCAs on the “chunkcounts.out” file output by ChromoPainter under the External analysis (Fig. 2) and the Internal analysis (figs. S4 and S5), using different subsets of samples. Following Lawson *et al.* (*47*), the matrix was first normalized by setting the diagonal (which is zero as an individual cannot be painted by themselves) to the average of each row and then subtracting the row means from the matrix. PCs were then calculated using the prcomp function in R.

### Inferring genetic similarity between individuals using the painting profile

The painting profiles from ChromoPainter can be used to measure genetic distance between any two recipient individuals. In particular, the “total variation distance” (TV*_ij_*) between individual 𝑖 and individual 𝑗 is calculated as the sum of the absolute difference in chunk lengths that 𝑖 and 𝑗 copy from each donor or group of donors (*9*, *29*). Specifically, if $f_{k}^{i}$ is the total proportion of genome-wide DNA that individual 𝑖 is inferred to match to individuals from group 𝑘

$${\mathrm{TVD}}_{ij}=0.5\sum_{k=1}^{K}|f_{k}^{i}-f_{k}^{j}|$$ (1)

Here, the groups, 𝑘 ∈ [1,…,], are 354 genetically homogenous raw fineSTRUCTURE clusters (see below; data S3). The value is standardized on a scale of 0 to 1 and presented throughout as a measure of similarity (1 − TVD*_ij_*). The similarity within a group (here, we use self-reported ethnic group or language classification) is the mean similarity across all pairings of individuals from the same group. Correspondingly, the similarity between two groups is the mean similarity across all pairings of individuals that come from different groups (figs. S8 and S9).

To assign significance to these genetic distances between groups, we used the following permutation tests used in López *et al.* (*8*). Group A can be defined as being genetically distinguishable from group B if the mean intragroup genetic similarity (𝐺) in A is significantly (*P* < 0.001) higher than the mean intergroup similarity between A and B; that is, when an individual is, on average, more similar to a member of their own group than to a member of the other group. Larger groups are more likely to have sampled a greater amount of genetic diversity. This sample size effect can occur irrespective of the actual diversity contained within the group; a large sample from a homogenous group could appear more diverse than a small sample from a heterogeneous group. To mitigate this effect, when comparing two groups, A and B, the following procedure was repeated for 100,000 permutations. The group with the larger sample size is first downsampled such that the two groups have the same 𝑛 = min (*n*_a_, *n*_b_), where *n*_a_ is the number of sampled individuals from group A. A permuted test group C is created with $\frac{n}{2}$ individuals randomly sampled without replacement from each of A and B. If 𝑛 is odd, $\frac{\left(n-1\right)}{2}$ individuals are sampled, and an extra individual is added randomly from either A or B such that C has *n* individuals. Having an equal number of individuals from groups A and B in the test group prevents the overrepresentation of either A or B increasing the mean similarity of the test group C. We made 100,000 separate C groups and found the proportion of times that the mean similarity among individuals in group C was greater than that of individuals in group A and (separately) individuals in group B. We use this proportion as a measure of significance, testing whether individuals from group A (respectively B) are no more genetically similar than a combined population of individuals from A and B. If A is significant, B will not necessarily be significant because of differences in intragroup similarity. For example, group A could be very homogenous, and thus, introducing individuals from group B always decreases the average similarity. However, if group B has high diversity, then some individuals from within group B are less similar to each other than they are to individuals in group A. Hence, an asymmetrical score can provide extra information about the relative diversity of each group. Permutation test results are shown in fig. S8, where black dots indicate a significance of 0.01, and gray dots indicate one of 0.001 (no multiple-testing correction was done here.)

For comparisons between language groups (fig. S9), an additional correction step is needed to remove ethnic group effects. High similarity between ethnic groups can inflate the similarity within language groups and thus drive any differences seen. The permutation test is run as before, except individuals from the same ethnic group are not considered in the similarity score for a language or for similarity scores during permutations. We note that this step will cause discrepancies in the number of pairwise comparisons in groups A, B, and C. However, group C will retain the same number of pairwise comparisons between language groups (as no ethnic groups speak multiple languages). Since, sometimes, a permutation randomly picks all individuals from the same ethnic group, permutations are repeated such that 100,000 group As and Bs, each containing at least two ethnic groups, are created for each language group comparison. If a language group consists of just one ethnic group, then permutation tests cannot be run.

### Testing the association between geographic and genetic distance

We used the distm function with the haversine formula from the R package geosphere to calculate the distance (in kilometers) between each pair of individuals in the dataset from Cameroon, Republic of the Congo, Ghana, Nigeria, and Sudan. We excluded previously published individuals without background information about grandparent birthplace (the Nigerian Yoruba and the Sudanese Dinka). Cameroon was split into two populations (dark red and light red), corresponding to the north and south, using both clustering (see below) and ethnic group information. These two groups have large differences in genetic variation patterns that would obscure any isolation-by-distance relationships seen within regions of Cameroon. A total of 114 individuals who had missing birthplace information or whose grandparents lived further than 150 km apart were excluded. Thirty Cameroonians with mean birthplace outside of Cameroon, or that clustered with northern Cameroonians but lived in the south (or vice versa), were also excluded. One Nigerian individual, who lived more than 400 km from all other pairs of Nigerians, was also excluded.

Within each country/region, we inferred the relationship between genetic similarity (1 − TVD) and geographic distance between pairs of individuals under three separate analyses: (i) where the two individuals in each pair were from different ethnic groups, (ii) where the two individuals were from the same ethnic group, and (iii) where both individuals were from different ethnic groups and lived within 20 km of the Nile river in Sudan. For each analysis, we grouped distances into 25-km bins and calculated, within each bin, the mean genetic similarity across all pairs of individuals separated by that distance and meeting criterion (i), (ii), or (iii). We modeled the correlation between genetic distance and geographic distance using a linear model and the R package ggpmisc to calculate the *R*^2^ of each line (figs. S10 and S11). For all analyses, bins where all comparisons involved the same individual were removed. For analysis (i), all distance bins with more than 100 pairwise comparisons were included. For analyses (ii) and (iii), bins with more than 20 pairwise comparisons were included.

### Defining genetically homogenous clusters at different levels

We used fineSTRUCTURE (*47*) to cluster the painting patterns produced by the ChromoPainter Internal analysis into genetically homogenous “populations.” Each population (cluster) contains individuals with indistinguishable genetic variation patterns under the model, i.e., they are inferred to be equally related to all members of their own cluster and to share similar relationships with all other clusters. The software also allows fixing certain groups as “superindividuals.” These are clusters that cannot be split up by the algorithm and are considered by the model as one individual, with a painting profile that is the average of those for all individuals in the superindividual. Using these fixed superindividuals can increase the power to detect fine-scale differences between the “free” (i.e. nonsuper) individuals.

To increase both the speed and power to detect structure, we ran four fineSTRUCTURE analyses with different sets of superindividuals. Individuals from each of the following countries/regions were left free to cluster, and the remaining superindividuals were defined using ethnic group label (see data S3):

1) Cameroon, Republic of the Congo, Ghana, and Nigeria
2) Sudan
3) Africa (excluding the above countries)
4) Non-Africa

For each analysis, fineSTRUCTURE was run with the normalization parameter “c” estimated as 0.244191687 for 3 million iterations. Clusters were sampled every 10,000 iterations after discarding the first 2 million iterations as “burn-in.” For the dendrogram-building (or tree-building) step, the algorithm was run for 1 million additional hill-climbing steps, after which fineSTRUCTURE merged clusters two at a time under a greedy approach that minimizes the loss in posterior probability. After each fineSTRUCTURE run, we used cluster assignments from all samples to reassign individuals to the cluster with the highest probability using the method described in Leslie *et al.* (*29*), resulting in 354 final raw clusters (figs. S12 and S13 and data S3).

We used both the above dendrograms and an individual’s self-reported ethnic group to define groups where all individuals (i) report the same ethnic group label and (ii) cluster on the same branch of the dendrogram. There are a few exceptions where one ethnic group was split into two based on differences in position on the fineSTRUCTURE dendrogram (Bamileke, Mbo, Tikar, Ja’aliya, Halfawieen, and Beni-Amer). For interpretability, individuals who clustered with a different ethnic group to what they self-identified as were removed from subsequent analyses described below, and ethnic groups that clustered together were separated into subclusters containing one ethnic group only. As a result, 367 of 5253 individuals were removed from subsequent analyses, including 190 individuals of the 1580 from Cameroon, Republic of the Congo, Ghana, Nigeria, and Sudan. Overall, this process resulted in 348 total clusters, with 101 of these from Cameroon, Republic of the Congo, Ghana, Nigeria, and Sudan (data S4).

### Calculating IBD sharing between individuals

For each of the 101 clusters defined above with >3 individuals (data S4), we inferred IBD segments using hap-ibd with default parameters (*49*). We then used the “merge-ibd-gaps” program to remove breaks and gaps in IBD segments, using parameters gap = 0.6 and discord = 1. We decomposed IBD segments into bins of different lengths (2 to 6 and >6 cM) (fig. S14).

### Modeling clusters’ inferred haplotype sharing as a mixture of that from other groups

To infer the amount of recent ancestry a target population shares with other reference groups, we used SOURCEFIND to model the painting profile of a target cluster as inferred by ChromoPainter as a mixture of those from a set of reference clusters (*50*). We applied SOURCEFIND to each of the 101 clusters from Cameroon, Republic of the Congo, Ghana, Nigeria, and Sudan described above, using the 226 of 348 surrogate clusters external to these five countries that had ≥4 individuals (data S4). This is because the lack of shared genetic drift in small clusters can favor them in the mixture model. Here, we used the ChromoPainter External painting (see text S3), in which the individuals from Cameroon, Republic of the Congo, Ghana, Nigeria, and Sudan were excluded as donors when painting each target individual. SOURCEFIND was run three independent times, each for 2 million iterations and sampling every 5000 iterations after discarding the first 50,000 iterations as “burn-in.” A maximum of eight reference clusters were allowed to describe the mixture composition of the target population at each iteration, with an a priori expectation of four reference clusters. For each cluster, we report the mean across the top 50 samples for the run (of three) with the highest average posterior probability. For results reported in data S6, mean mixture components that summed to less than 1% in all clusters were grouped together as “other" (gray).

We used the results from this SOURCEFIND analysis to create 18 supergroups by merging groups where inferred recent ancestry sharing with reference groups appears similar (fig. S15). These larger groups (see Fig. 3 and data S5) give us more power to infer and date any admixture events present (see below). We then reran SOURCEFIND on these supergroups in the same way as above for Fig. 5C. These supergroups were then used for all admixture analyses below.

### Identifying and dating admixture events

We inferred admixture events in the history of our 18 supergroups using each of fastGLOBETROTTER (*27*), MOSAIC (*25*), and MALDER (*26*). In all cases, fastGLOBETROTTER was run for five mixing iterations, using 240 clusters as potential surrogates to the admixing sources (data S4). We performed 100 bootstrap resamples to generate 95% confidence intervals around the inferred dates. We used null.ind = 1, which aims to eliminate any signals associated with LD decay that are not attributable to genuine admixture. We only included results with a maxR2fit.1date of greater than 0.5, which indicates a good fit of the theoretical admixture model to observed LD decay patterns. To be conservative when concluding two dates of admixture, we also increased the default maxScore.2events cutoff that is used to determine whether admixture events at multiple times occurred, to 0.4 (default = 0.35). We initially used a curve.range of 25 for all tests, which models LD decay at segments separated by ≤25 cM to infer admixture. However, for target populations with an inferred dated older than 40 generations ago under this analysis, we reran (and report results from) fastGLOBETROTTER using a curve.range of 15, since admixture LD decays more rapidly for older events. Assuming that a fastGLOBETROTTER inferred date of 𝑔 generations, the year (𝑌) of admixture was calculated as 𝑌 = 𝐷 − 28 × (𝑔 + 1), where 𝐷 is the average birth year among sampled individuals (*D* = 1968) and generation time is set to 28 years (*88*).

When running MOSAIC, for computational efficiency, we used a set of 14 clusters as admixture surrogates {Ethiopia_Anuak, Malawi_Tumbuka, Gambia_Mandinka3, Tunisian, Yemen, Ethiopia_Tigray, Mende_Sierra_Leone, Morocco_Berber, Uganda_Baganda, Namibia_Wambo, BiakaPygmy, Egyptian, Somali, Saudi1}, picked because they were inferred as representative surrogates to ancestral sources in each supergroup using SOURCEFIND. For all supergroups, we ran MOSAIC with both two and three admixing sources and picked the result with the greatest expected genome wide *r*^2^. We used the bootstrap_individuals_coanc_curves function in the MOSAIC R package to calculate confidence intervals for the inferred dates. We excluded results with an expected genome-wide *r*^2^ of less than 0.5, and where the Rst between sources, a measure of how well inferred sources are differentiated using these reference groups, was less than 0.01.

We ran MALDER on the 17 supergroups using a list of 22 groups as reference populations for computational efficiency, {Turkish1, Ethiopia_Nuer, Botswana_Taa_North, Mozambique, Zimbabwe, Ethiopia_Gurage, Kenya_Bantu, Ethiopia_Anuak, Malawi_Tumbuka, Gambia_Mandinka3, Yemen, Ethiopia_Tigray, Mende_Sierra_Leone, Morocco_Berber, Uganda_Baganda, Namibia_Wambo, BiakaPygmy, Egyptian, Somali, Saudi1, MbutiPygmy, Gambia_Fula1}. Results with the largest two-reference curve amplitude were picked for each cluster, though we note these chosen reference pair combinations may not be a significantly better fit to the data than other reference pair combinations. We used a *P* value cutoff of 0.01. To calculate the 95% confidence interval, we multiplied the standard error by 1.96. We concluded two dates if there were two events inferred where neither the date confidence interval nor the two-reference curve amplitude confidence interval overlapped. We do not report cases where MALDER infers a date older than 200 generations, which would have little remaining signal of admixture LD decay. This occurred in one case, where MALDER inferred admixture 203 generations old, involving a source related to non-Africans, in the Nigeria West cluster.

To investigate admixture in the group of Sudanese who cluster with the Cameroon Fulani (Sudanese clustering near Fulani in fig. S13), we ran fastGLOBETROTTER using the Internal painting described above, where target individuals were painted against all other sampled individuals, including those from Cameroon, Ghana, Nigeria, Republic of the Congo, and Sudan. The 100 clusters containing people from these five countries were also included as surrogates to the admixing sources, excluding the one cluster containing the target individuals. We also ran MALDER and MOSAIC, not only using the same reference populations described above but also including the 17 other supergroups as references. This was so we could detect admixture at (plausibly) more local scales between the different populations present in northern Cameroon (results in data S7). For MALDER, the results were the same between the two analyses, but for fastGLOBETROTTER and MOSAIC, we inferred a different, more recent admixture event. We report these results that used Cameroonian surrogate populations for the Sudanese clustering near Fulani in Fig. 5A; for all other clusters in Fig. 5A, we report results from the analyses described above that exclude Cameroonian surrogates.

### Genetic variation patterns in Bantu-speaking populations

We also used SOURCEFIND to model genetic variation patterns in 14 groups (shown in Fig. 7) and four ancient individuals (Eland Cave, Mfongosi, and Newcastle from South Africa and Deloraine Farm from Kenya), whose individuals either speak a Bantu language or were previously reported to share Bantu-related ancestry (e.g., Damara and Luo) (*10*, *13*, *15*, *50*). We first ran ChromoPainter on the whole dataset, but excluding matching to any of the above 18 groups/individuals (Ne = 182.82, Mu = 0.000576034). The purpose of this analysis was to examine whether these Bantu-related target populations matched to similar nonlocal reference groups, e.g., in Cameroon. We ran SOURCEFIND using this painting as input, and 270 surrogate clusters that had ≥4 individuals, including 262 non-Bantu speaking groups and 8 Bantu-speaking groups from Cameroon. We also excluded non-Bantu–speaking southern African populations with previously reported Bantu-speaking related ancestry [leaving only Ju_Hoan, Taa, Naro, and Xuun from southern Africa (*89*)], to avoid overestimating the proportion of “local” (i.e. non-Bantu–related) ancestry in southern African Bantu-speaking peoples (data S4). As before, we ran three independent runs of two million iterations each, sampling every 5000 iterations after discarding the first 50,000 iterations as “burn-in.”

We then used fastGLOBETROTTER, MOSAIC, and MALDER to infer and date admixture events in the 13 groups (excluding Congo, which was already analyzed in analyses described above) and four ancient individuals. Using the ChromoPainter results of this section, we ran fastGLOBETROTTER on each of the 17 groups, using 304 reference groups as surrogates to the admixing sources (data S4). We used a curve.range of 15 and the parameters used in our previous analysis described above (results in data S7). For the four ancient individuals, we used the chromosome jackknife resampling option rather than bootstrapping to infer confidence intervals, since bootstrapping cannot be done on a single individual. To mimic previous analyses (*5*, *15*, *23*, *30*) exploring the route of the initial expansion of Bantu speakers, we also ran fastGLOBETROTTER on Kenya Kikuyu and Zimbabweans using the 304 reference groups mentioned above, plus (i) 11 clusters from Congo or (ii) all 14 groups whose individuals either speak a Bantu language or were previously reported to share Bantu-related ancestry and the 4 ancient individuals (fig. S18).

When running MOSAIC and MALDER, we included a set of local admixture surrogates that were inferred as important ancestry surrogates in SOURCEFIND, 11 in MOSAIC {BiakaPygmy, MbutiPygmy, Botswana_Taa_East, Tanzania_Sandawe, Namibia_Xuun, Gambia_Mandinka3, Ethiopia_Tigray, Ethiopia_Anuak, Kenya_Sengwer, Kenya_Maasai, Yemen} and 12 in MALDER {MbutiPygmy, BiakaPygmy, Botswana_Taa_North, Namibia_Xuun, Gambia_Mandinka3, Mende_Sierra_Leone, Ethiopia_Amhara, Ethiopia_Gumuz, Ethiopia_Gurage, Ethiopia_Nuer, Kenya_Sengwer, Kenya_Maasai}. For both analyses, we also included a set of 11 Bantu- and Grassfields- speaking groups from southern Cameroon, to see which was the best representative of Bantu speaking–like ancestry {Cameroon_Mambila, Cameroon_Noni, Cameroon_Bamun, Cameroon_Mbo1, Cameroon_TikarEast, Cameroon_Bafut, Cameroon_Bassa, Cameroon_BamilekeNorth, Cameroon_ BamilekeSouth, Cameroon_Bonguili, Cameroon_Bulu}. We ran MOSAIC using both two and three admixture sources and picked the result with the greatest *r*^2^. As before, we excluded events with an expected genome-wide *r*^2^ of less than 0.5 and where the Rst between sources was less than 0.01. MALDER could not be run on the ancient individuals. For the 13 present-day groups, for MALDER, we picked the highest two-reference curve amplitude for each cluster and used the same filtering steps as above.

### Inferring historical changes in N_e_

We used GONE (*55*) and IBDNe (*56*) to infer historical effective population size changes in Republic of the Congo (fig. S20). Among pairs of Congolese with a Plink v1.9 (*86*) PI_HAT > 0.05, we excluded one individual per pair to prevent cryptic relatedness within populations potentially decreasing recent population size estimates. The plink b37 genome build was used to generate genetic distances for the .map files. GONE was run using the standard parameters and PHASE = 2 (unknown phase), including 40 replicates. IBDNe was run using the IBD segments inferred above, the parameters “filtersamples = true” and a minimum length of 2 cM. We disregarded results from more than 100 generations ago, as these have been suggested to be unreliable (*55*).

### Simulating the spread over spread model of the Bantu expansion

We used msprime (*90*) to simulate 30 independent chromosomes, each 100 mega-base pairs (Mbp) in length to roughly mimic the size of the human genome. Code used for simulations can be found in text S4. Four different scenarios were simulated to match the scenario proposed by Seidensticker *et al*. (*53*) and Saulieu *et al.* (*54*) of two different waves of expansion into the Congo basin. The first expansion is simulated to begin roughly 100 generations ago (800 BCE), followed by a bottleneck beginning 50 generations ago (560 CE). The second expansion then begins 25 generations ago (1260 CE). The strength of bottleneck and first and second expansion sizes was varied (fig. S21).

After running the simulation in msprime, the output vcf was downsampled such that the number of SNPs in each 0.05 minor allele frequency bin matched that in our data of individuals from Congo to ensure that the simulated data mimicked array data. Effective population size changes were then inferred from this simulated dataset using GONE and IBDNe (as above).

## Supplementary Materials

### This PDF file includes:

Text S1 to S4 Figs. S1 to S21 Legend for fig. S6 Legends for data S1 to S8 References

## Notes

View/request a protocol for this paper from *Bio-protocol*.
