# Supplementary material for: Dense sampling of ethnic groups within African countries reveals fine-scale genetic structure and extensive historical admixture
Source: Sci Adv. 2023 Mar 29;9(13):eabq2616. doi: 10.1126/sciadv.abq2616 (PMC10058250; doi:10.1126/sciadv.abq2616)
Supplement: Supplementary file 1 — Text S1 to S4 Figs. S1 to S21 Legend for fig. S6 Legends for data S1 to S8 References [file sciadv.abq2616_sm.pdf]

Science Advances
AAAS

Supplementary Materials for

Dense sampling of ethnic groups within African countries reveals fine-scale genetic structure and extensive historical admixture

Nancy Bird *et al.*

Corresponding author: Nancy Bird, nancy.bird.18@ucl.ac.uk

Sci. Adv. **9**, eabq2616 (2023)
DOI: 10.1126/sciadv.abq2616

The PDF file includes:

Supplementary Text S1 to S4
Figs. S1 to S21
Legend for fig. S6
Legends for data S1 to S8
References

Other Supplementary Material for this manuscript includes the following:

Fig. S6
Data S1 to S8

Supplementary Text 1

Brief description of newly reported samples from each country

Ghana

The project includes newly reported data from 211 individuals sampled in Ghana and representing 21 ethnic groups, 16 of which have a sample size of more than three individuals. Ethnic groups represented by data from more than 15 sampled individuals include: Dagaati, Frafra, Kusaasi, Bulsa and Wali. All included ethnic groups speak a Niger-Congo language. They are divided into two main linguistic branches: North Volta-Congo speakers and Kwa Volta-Congo speakers from the north and south of the country respectively (*33*). Within this, all North Volta-Congo speakers speak a Gur language, with the majority of these being Northern Central Gur languages. The exceptions are Sisaala and Kasem which are both Southern Central Gur. Of the Kwa Volta-Congo speakers, the majority of individuals speak a Nyo language, while one ethnic group, the Ewe speak a Gbe language.

Nigeria

Newly reported data was generated from 311 Nigerian individuals, from 6 different ethnic groups. These include: Anang, Efik, Ekoi, Ibibio, Igbo, and Oron. All ethnic groups have data from more than 15 individuals, with the largest group, Ibibio, having data from 161 individuals. All groups are from the South South and Southeast regions of Nigeria, with the majority sampled in the Cross River State. All ethnic groups speak a Niger-Congo language. Within this, most ethnic groups speak a Delta Cross language, while the Ekoi speak a Bantoid language and the Igbo speak an Igboid language.

Republic of the Congo

Newly reported genetic data was generated from 122 individuals from 17 ethnic groups sampled in the Republic of the Congo. Of these, 11 groups have a sample size of more than three individuals, but only one, the Yombe, has more than 15 individuals. All ethnic groups speak a Central-Western Bantu language. Of these, all are West-Coastal Bantu speakers except the Mboshi, who speak North Zaire River Bantu. Within the West-Coastal Bantu speakers, ethnic groups are divided into Njebe-Mbete-Teke language speakers (Koukouya, Lali, Mbere and Teke) and Kongo-Yaka-Suku-Hungan speakers (all other groups). The majority of samples have birthplaces in the southern half of the country.

Cameroon

Genetic data from Cameroon were obtained from individuals sampled as part of a North-South transect. The data contains newly reported genotype data from 484 Cameroonians from 77 different ethnic groups. 30 of these groups have data from more than three individuals, with nine represented by more than 15 individuals: Kotoko, Mambila, Noni, Bamun, Tikar, Yamba, Nso, Wimbum and Kwandja. Sampled groups include speakers of three main African language families; Afro-Asiatic, Nilo-Saharan, and Niger-Congo. The former two all inhabit the north of the country. There are two main branches of Nilo-Saharan speakers. Firstly, the Kanuri speak a Saharan language, and secondly the Sara and Ngambai are Central Sudanic speakers. Of the Afro-Asiatic speakers, one ethnic group, the Arabe, speak Arabic. All other Afro-Asiatic

speakers have languages that are members of the Chadic branch. Most notably, these include Guidar, Kotoko, Mandara, Massa and Mousgoum.

The Niger-Congo speakers primarily inhabit the southern half of Cameroon. An exception is Fulani, who speak a North-Central Atlantic language (primarily found in Senegal). Another exception are Ubangian speakers, such as Doowaayo, Moundang, and Toupouri, who also live in the northern half of Cameroon. The remaining Niger-Congo speakers are then divided into two main classifications; Northern Bantoid and Southern Bantoid. Northern Bantoid speakers live primarily in the Adamawa region near the border with Nigeria, and include the ethnic groups Kwandja, Mambila and Tikar. Southern Bantoid speakers are again divided into two main groups: Wide Grassfields and Narrow Bantu. Wide Grassfields speakers primarily inhabit the Northwest and West regions of Cameroon and include the Bamileke, Bamun, Nso', Wimbum and Yamba. Lastly, the Narrow Bantu speakers include Bassa, Ewondo, Mbo and Ngoumba. The Narrow Bantu speakers in our dataset inhabit the Southwest, South and Littoral regions. Narrow Bantu is the language family spoken by roughly 30% of the African population as a result of the expansion of Bantu-speaking peoples. It originated in the Nigeria/Cameroon border region (*76*). However, there is much debate over the classification of Wide Grassfields and Narrow Bantu languages (*52*).

Sudan

The project includes data from 233 individuals sampled in Sudan representing 31 ethnic groups. 17 of these groups have data from more than 3 individuals, with 4 having data from 15 or more individuals: Ja'aliya, Halfawieen, Beni-Amer and Rubatab. Sudanese individuals were primarily sampled as part of a Nile transect, where samples were collected at different cities and towns along the river (n=144, samples from Abu Hamad, Atbara, Dongola, Ad Douiem, Kosti, Madani, Shendi and Wadi Halfa). Other samples (mostly from Beni-Amer) were collected at the coast in Port Sudan. Lastly, samples were collected from individuals in the South Kordofan region, near the border with South Sudan. The majority of the sampled Sudanese individuals speak Arabic. Within Arabic-speakers, there are two separate ethnicities related to the pre-Arabic language spoken: Beja, and Nubian. Beja ethnic groups include Beni-Amer, Hadendowa and Halenga. Nubian ethnic groups include Danagla and Halfawieen. Ethnic groups represented in this study from the south Kordofan region speak either Nilo-Saharan or Kordofanian languages. Within Nilo-Saharan speakers, the Lagori speak a Dajuic language and the Kadugli, Keiga and Korongo speak a Kadugli-Krongo language. Within Kordofanian languages, the Acheron speak a Talodi language and the Moro speak a Heibanic language. We have grouped the Kordofanian languages into one phyla for this paper, although there is much debate about whether they constitute a single branch and some evidence indicates it may be a group of language isolates (*36*).

Note: Languages were classified using Glottolog for all countries except Sudan, where Ethnologue was used. This is because we inferred genetic differentiation between languages from the south Kordofan region classified as Nilo-Saharan and Kordofanian in Ethnologue. This level of classification is not included in Glottolog, as it is more disputed.

Supplementary Text 2

Brief description of the historical polities mentioned in the text

Grassfields of Cameroon

The Grassfields are situated in broadly the North-West and West regions of Cameroon. The region is characterised by mountainous terrain and Sudanian savannah vegetation. It is home to many different ethnolinguistic groups living in close proximity (*35*). Historically, these groups have adopted a number of different political systems, and have spheres of influence of varying sizes. Some groups, such as the Bamun, ‘Nso, and Bafut had large polities in pre-colonial times, and were involved in both trade and conflict with other nearby groups (*62*).

Makuria and the fall of Dongola

Makuria was a Christian Nubian Kingdom established in the 4th century CE. It was located along the Nile, and at its height extended from the area around modern Khartoum to north of Luxor in what is now Egypt. Its capital was the city of Dongola (*67*). Arabs expanded into Egypt in the 7th century but formed a peace treaty with Makuria, preventing further expansion along the Nile (*91*). However, the kingdom began to decline from the 13th century onwards. The history of the Christian Makurian state from the 14th century onward is complex, with repeated Mamluk and Bedouin invasions and civil strife, and it had disappeared by the early 16th century.

Kingdom of Aksum

Also known as the Axumite Empire, this kingdom was regionally dominant from 100CE to about 800CE. It originated in northern Ethiopia, although as it expanded it incorporated parts of modern Eritrea and eastern Sudan. At the height of the empire in the 500CEs it also incorporated part of the southern Arabian peninsula. It was involved in trade networks linking the Roman Empire to the Middle East and India, and had a strong navy. The empire was linguistically and ethnically complex, although Ge’ez, an Afro-Asiatic Semitic language, was the official language of imperial rule and the Church (*68*). The period up to the peak of the empire was characterized by population growth (*92*).

Kanem-Bornu Empire

The Kanem-Bornu Empire existed in two phases. From the eighth century CE to approximately 1380CE, the political center of gravity of the state existed northeast of Lake Chad and the state was dominated by Nilo-Saharan-speaking Kanembu people. After 1380CE, the empire gradually moved its centre to Bornu in Nigeria, southwest of Lake Chad, traditionally held to be a result of attacks from Bulala people. This movement led to interactions with, and assimilation of, local Chadic-speaking people under the collective name of Sao, who lived in northern Cameroon and Nigeria. The merging of Nilo-Saharan-speaking people from the empire, and local Sao and related groups, gave rise to the Kanuri ethnic group. The empire covered parts of Niger, Cameroon, Central Africa Republic, Chad and Nigeria at different times and was an important trading centre. It was a natural connecting point for trans-Saharan and Sudanic trade routes (*38, 71*).

Fulani Jihad and Sokoto Caliphate

The Fulani Jihad occurred between 1804-1808 in Nigeria and continued throughout much of the C19th, eventually reaching present-day Cameroon. It was initially a conflict between armies led

by the Fulani scholar Usman Dan Fodiyo and the Hausa kingdoms of northern Nigeria, and led to the creation of the multi-ethnic Sokoto Caliphate in 1804. Sokoto dominated much of west-central Africa in the 19th century, stretching from the Niger River in the west to the Adamawa Plateau in the southeast and engaging in warfare and slave-raiding with neighboring populations. The Sokoto Caliphate ended when the British and Germans conquered its territory between 1891 and 1903 (*73*).

Supplementary Text 3

ChromoPainter analyses

ChromoPainter uses haplotype sharing patterns to represent (or 'paint') each of a focal individual's two haploid genomes as a mosaic of 'chunks' of DNA copied from a set of other so-called 'donor' haploid genomes in the population. A chunk is a set of contiguous SNPs copied from the same donor haploid, with the donor of each chunk changing as one moves along the focal individual's chromosome to reflect historical recombination events (*93*). At each genomic location, the donor copied is inferred to be the one that shares a most recent ancestor (i.e., more recent than any other donor haploid) with the focal individual. Chromosome painting can detect fine-scale genetic structure missed by other commonly used methods based on independent SNPs and is less susceptible to biases as a result of SNP array ascertainment (*8, 29, 47*). ChromoPainter requires two scaling parameters, the switch parameter, Ne ('-n'), which controls the rate of switching from donor to donor within a focal haploid, and the mutation parameter, θ ('-M'), which controls how often a focal haploid is allowed to copy from a donor that carries a different allele from that of the focal haploid.

Analogous to van Dorp et al. (*9*), for this study we performed two separate chromosome paintings, each comparing individuals to a different set of donors:

1. **Internal:** all individuals in the dataset are painted against (i.e., compared to) every other individual (Ne= 172.735, Mu= 0.000619838).
2. **External:** individuals are painted without allowing donors from Cameroon, Republic of the Congo, Ghana, Nigeria and Sudan (3673 donor individuals, 281 populations represented) (Ne= 213.426, Mu= 0.000676708).

Under the internal painting, isolated groups are likely to copy long segments from other individuals within the same ethnic group or geographic region, which may make their painting appear very different to individuals from other nearby ethnic groups. The external painting acts to mitigate this effect by preventing individuals copying from their own or nearby groups (*9*). Consistent with this, when calculating a measure of genetic similarity based on the chromosome paintings (1-TVD, Methods), the mean value for individuals from Cameroon, Republic of the Congo, Ghana, Nigeria, and Sudan is 0.57 for the internal painting and 0.77 for the external painting, indicating higher genetic similarity in the latter. Thus, the external painting can reveal older relationships among populations not affected by recent isolation. Furthermore, by excluding neighbouring populations as donors, the external painting can also highlight ancestry derived from sources plausibly originating outside of these cases (e.g. due to admixture).

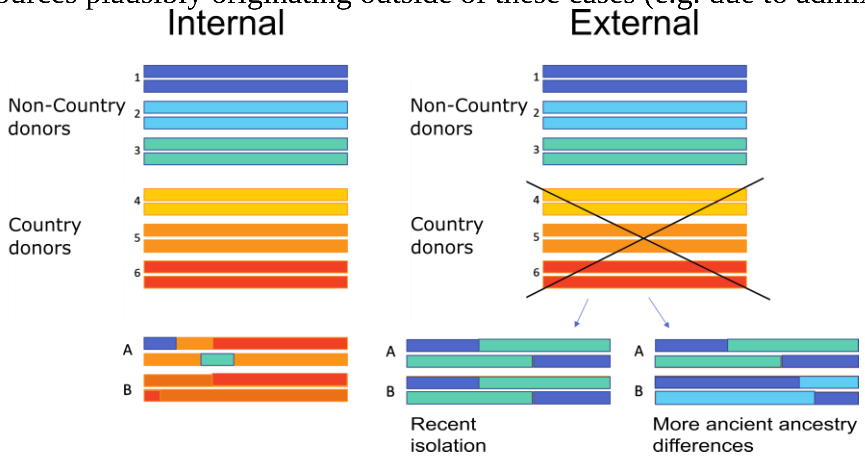

In the ‘internal’ analysis (Figure above left), ChromoPainter uses individuals 1-6 as donors with which to paint individuals A and B. Each haploid (row) of A and B is constructed as a mosaic of the donors’ haploid genomes. In the ‘external’ analysis (above right), the recipients can only be painted with donors from outside of the recipient’s country or region. Individual A and B will on average look more similar to each other in the external analysis as the effects of recent isolation are mitigated. Alternatively, if A and B have different sources of ancestry related to the non-Country donors (e.g. due to separate admixture events), they may still look different under the external analysis.

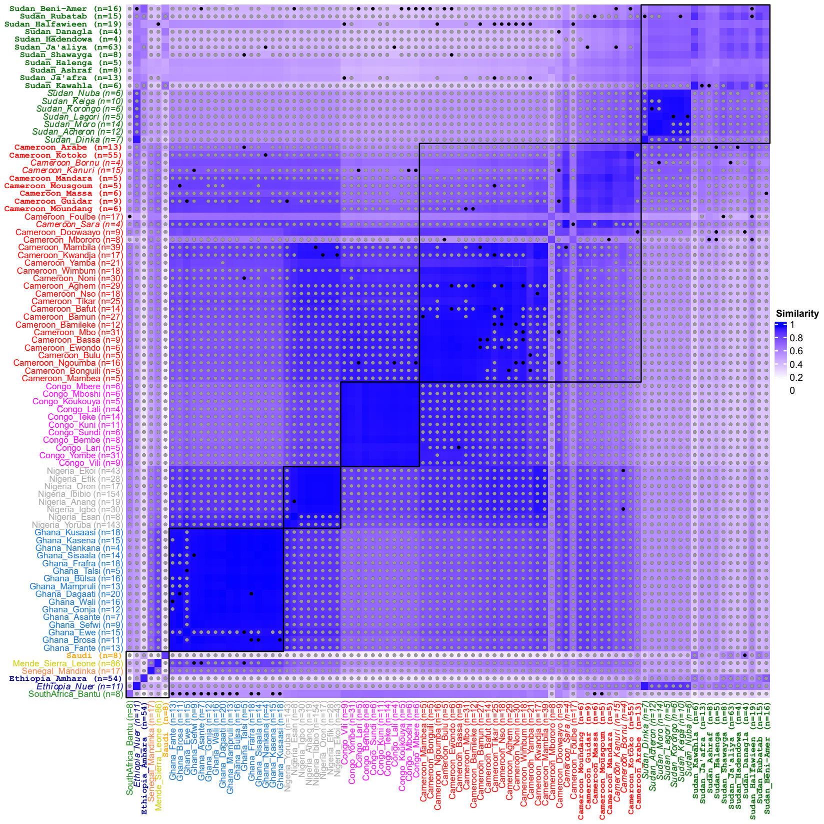

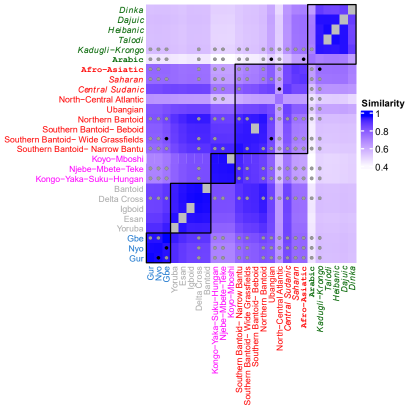

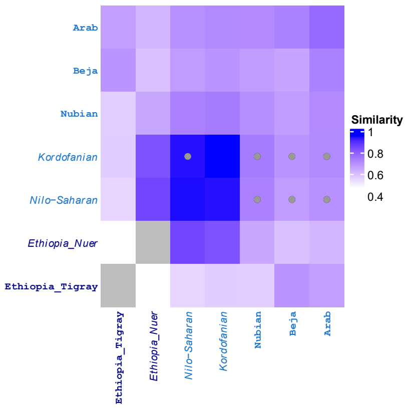

Above are heatmaps analogous to the ones shown in Fig. S8-9 except using the ‘external’ painting described above. The mean similarity between ethnic groups is higher in these heatmaps, and patterns of population structure differ. For example, in Fig S8 many of the ethnic groups in Ghana are genetically differentiable, but the majority of this structure disappears in the above heatmap, suggesting it is driven by endogamy/isolation rather than differences in ancestry. This is also seen in southern Cameroon, where genetic similarity between ethnic groups increases in the external analysis, indicating similar patterns of ancestry in this region. However, in some cases genetic structure is still detected when using the external painting, for example the structure between Arabic and Kordofanian-speaking Sudanese and between ethnic groups from different countries. In these cases, it suggests the genetic differences are driven by more external differences in ancestry rather than simple isolation/endogamy.

Another interesting result from the external analyses is in the heatmap of genetic differences between Sudanese language families. When comparing Kordofanian and Nilo-Saharan speakers, they have a high genetic similarity but are still differentiable, with the Nilo-Saharan speakers having a greater similarity to the Nilo-Saharan-speaking Ethiopian Nuer. This suggests that these two Sudanese groups, in addition to some degree of recent isolation, may have some older differences related to speaking languages from different phyla.

The external painting was used as the input for the SOURCEFIND results in Fig. 5 and Fig. S15 and the fastGLOBETROTTER analyses (Methods). This is because it allows detection of admixture events between sources more external to the focal group, which are likely to be older, rather than focusing on recent mixture between more proximate sources (*9*).

Supplementary Text 4

Code for msprime simulations in Fig. S21

(A)

```
mu=1.25e-8 # mutation rate per bp
rho=1e-8 # recombination rate per bp
nbp = 1e8
demography = msprime.Demography()
demography.add_population(name="A", initial_size=1000000, growth_rate=0.2763102)
demography.add_population_parameters_change(time=25, population="A", growth_rate=0,
initial_size=1000)
demography.add_population_parameters_change(time=45, population="A", initial_size=1000,
growth_rate=-0.9868222)
demography.add_population_parameters_change(time=52, population="A",
initial_size=1000000, growth_rate=0)
demography.add_population_parameters_change(time=68, population="A",
initial_size=1000000, growth_rate=0.163001)
demography.add_population_parameters_change(time=92, population="A", initial_size=5000,
growth_rate=0)
demography.sort_events()
ts = msprime.sim_ancestry({"A": 100}, demography=demography, recombination_rate=1e-8,
sequence_length=nbp, random_seed=seed1)
ts_mutated=msprime.sim_mutations(ts, rate=mu, random_seed=seed2)
```

(B)

```
mu=1.25e-8 # mutation rate per bp
rho=1e-8 # recombination rate per bp
nbp = 1e8
demography = msprime.Demography()
demography.add_population(name="A", initial_size=1000000, growth_rate=0.1564809)
demography.add_population_parameters_change(time=25, population="A", growth_rate=0,
initial_size=20000)
demography.add_population_parameters_change(time=45, population="A", initial_size=20000,
growth_rate=-0.5588604)
demography.add_population_parameters_change(time=52, population="A",
initial_size=1000000, growth_rate=0)
demography.add_population_parameters_change(time=68, population="A",
initial_size=1000000, growth_rate=0.163001)
demography.add_population_parameters_change(time=92, population="A", initial_size=5000,
growth_rate=0)
demography.sort_events()
ts = msprime.sim_ancestry({"A": 100}, demography=demography, recombination_rate=1e-8,
sequence_length=nbp, random_seed=seed1)
ts_mutated=msprime.sim_mutations(ts, rate=mu, random_seed=seed2)
```

(C)

```
mu=1.25e-8 # mutation rate per bp
rho=1e-8 # recombination rate per bp
nbp = 1e8
demography = msprime.Demography()
demography.add_population(name="A", initial_size=100000000, growth_rate=0.3406877)
demography.add_population_parameters_change(time=25, population="A", growth_rate=0,
initial_size=20000)
demography.add_population_parameters_change(time=45, population="A", initial_size=20000,
growth_rate=-0.5588604)
demography.add_population_parameters_change(time=52, population="A",
initial_size=1000000, growth_rate=0)
demography.add_population_parameters_change(time=68, population="A",
initial_size=1000000, growth_rate=0.163001)
demography.add_population_parameters_change(time=92, population="A", initial_size=5000,
growth_rate=0)
demography.sort_events()
ts = msprime.sim_ancestry({"A": 100}, demography=demography, recombination_rate=1e-8,
sequence_length=nbp, random_seed=seed1)
ts_mutated=msprime.sim_mutations(ts, rate=mu, random_seed=seed2)
```

(D)

```
mu=1.25e-8 # mutation rate per bp
rho=1e-8 # recombination rate per bp
nbp = 1e8
demography = msprime.Demography()
demography.add_population(name="A", initial_size=1000000, growth_rate=0.1564809)
demography.add_population_parameters_change(time=25, population="A", growth_rate=0,
initial_size=20000)
demography.add_population_parameters_change(time=45, population="A", initial_size=20000,
growth_rate=-0.5588604)
demography.add_population_parameters_change(time=52, population="A",
initial_size=1000000, growth_rate=0)
demography.add_population_parameters_change(time=68, population="A",
initial_size=1000000, growth_rate=0.1248222)
demography.add_population_parameters_change(time=92, population="A", initial_size=50000,
growth_rate=0)
demography.sort_events()
ts = msprime.sim_ancestry({"A": 100}, demography=demography, recombination_rate=1e-8,
sequence_length=nbp, random_seed=seed1)
ts_mutated=msprime.sim_mutations(ts, rate=mu, random_seed=seed2)
```

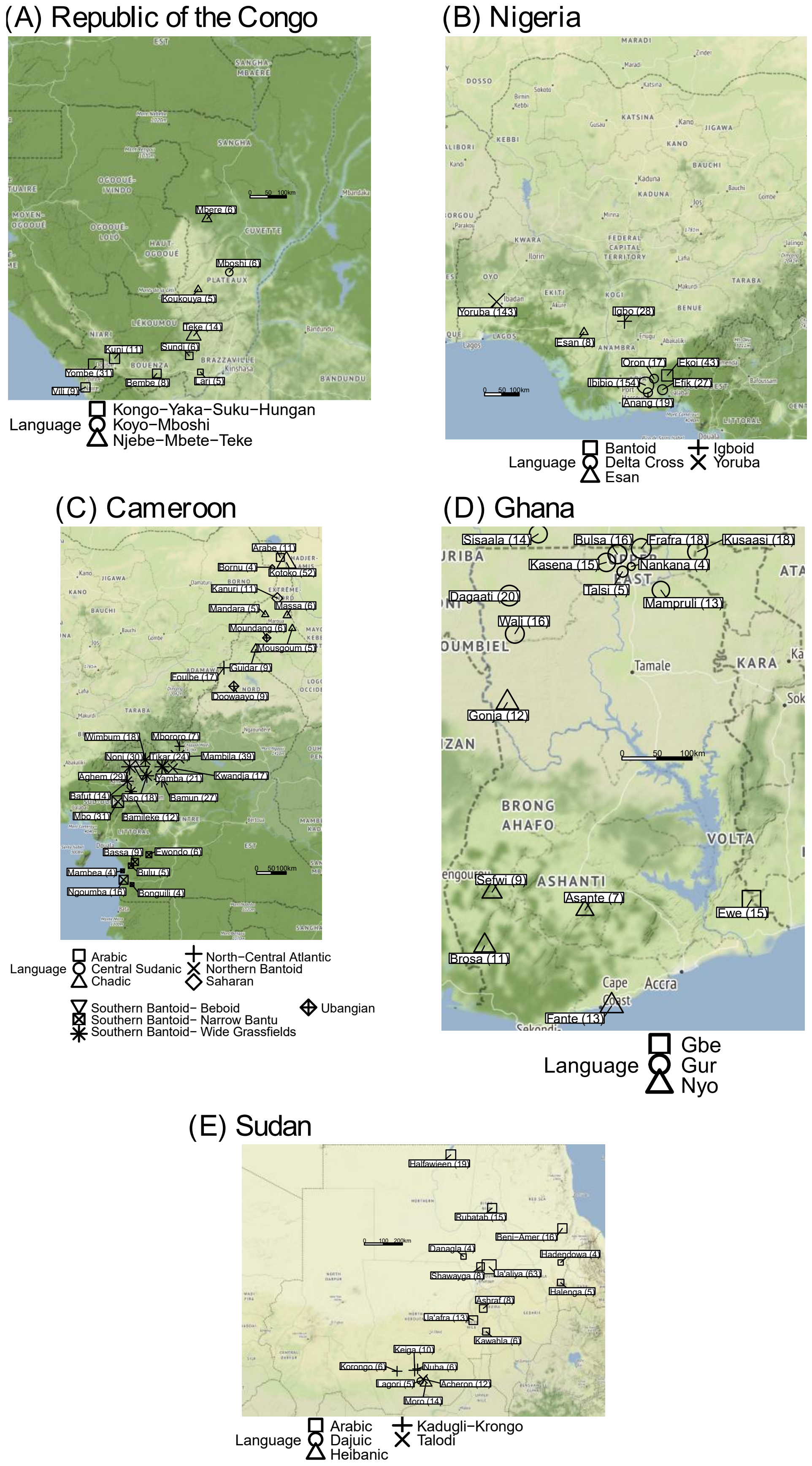

(A) Republic of the Congo
Mbere (6)
Mboshi (6)
Koukouya (5)
Teke (14)
Kuni (11)
Sundi (6)
Yombe (31)
Bembe (8)
Lari (5)
Vili (9)
Language
Kongo−Yaka−Suku−Hungan
Koyo−Mboshi
Njebe−Mbete−Teke
(B) Nigeria
Yoruba (143)
Igbo (28)
Esan (8)
Oron (17)
Ekoi (43)
Ibibio (154)
Efik (27)
Anang (19)
Language
Bantoid
Delta Cross
Esan
Igboid
Yoruba
(C) Cameroon
Arabe (11)
Bornu (4)
Kotoko (52)
Kanuri (11)
Mandara (5)
Massa (6)
Moundang (6)
Mousgoum (5)
Guidar (9)
Foulbe (17)
Doowaayo (9)
Wimbum (18)
Mbororo (7)
Noni (30)
Tikar (24)
Mambila (39)
Aghem (29)
Yamba (21)
Kwandja (17)
Bafut (14)
Nso (18)
Bamun (27)
Mbo (31)
Bamileke (12)
Bassa (9)
Ewondo (6)
Mambea (4)
Bulu (5)
Ngoumba (16)
Bonguili (4)
Language
Arabic
Central Sudanic
Chadic
North−Central Atlantic
Northern Bantoid
Saharan
Southern Bantoid− Beboid
Southern Bantoid− Narrow Bantu
Southern Bantoid− Wide Grassfields
Ubangian
(D) Ghana
Sisaala (14)
Bulsa (16)
Frafra (18)
Kusaasi (18)
Kasena (15)
Nankana (4)
Talsi (5)
Dagaati (20)
Mampruli (13)
Wali (16)
Gonja (12)
Sefwi (9)
Asante (7)
Ewe (15)
Brosa (11)
Fante (13)
Language
Gbe
Gur
Nyo
(E) Sudan
Halfawieen (19)
Rubatab (15)
Beni−Amer (16)
Danagla (4)
Hadendowa (4)
Shawayga (8)
Ja'aliya (63)
Halenga (5)
Ashraf (8)
Ja'afra (13)
Kawahla (6)
Keiga (10)
Korongo (6)
Nuba (6)
Lagori (5)
Acheron (12)
Moro (14)
Language
Arabic
Dajuic
Heibanic
Kadugli−Krongo
Talodi

Fig. S1.

Map of samples from (A) Republic of the Congo, (B) Nigeria, (C) Cameroon, (D) Ghana and (E) Sudan. Each point indicates an ethnic group, placed according to the mean birthplaces of its individuals' maternal grandmothers and paternal grandfathers. Only ethnic groups that contain more than three individuals are shown. Point shape shows language family.

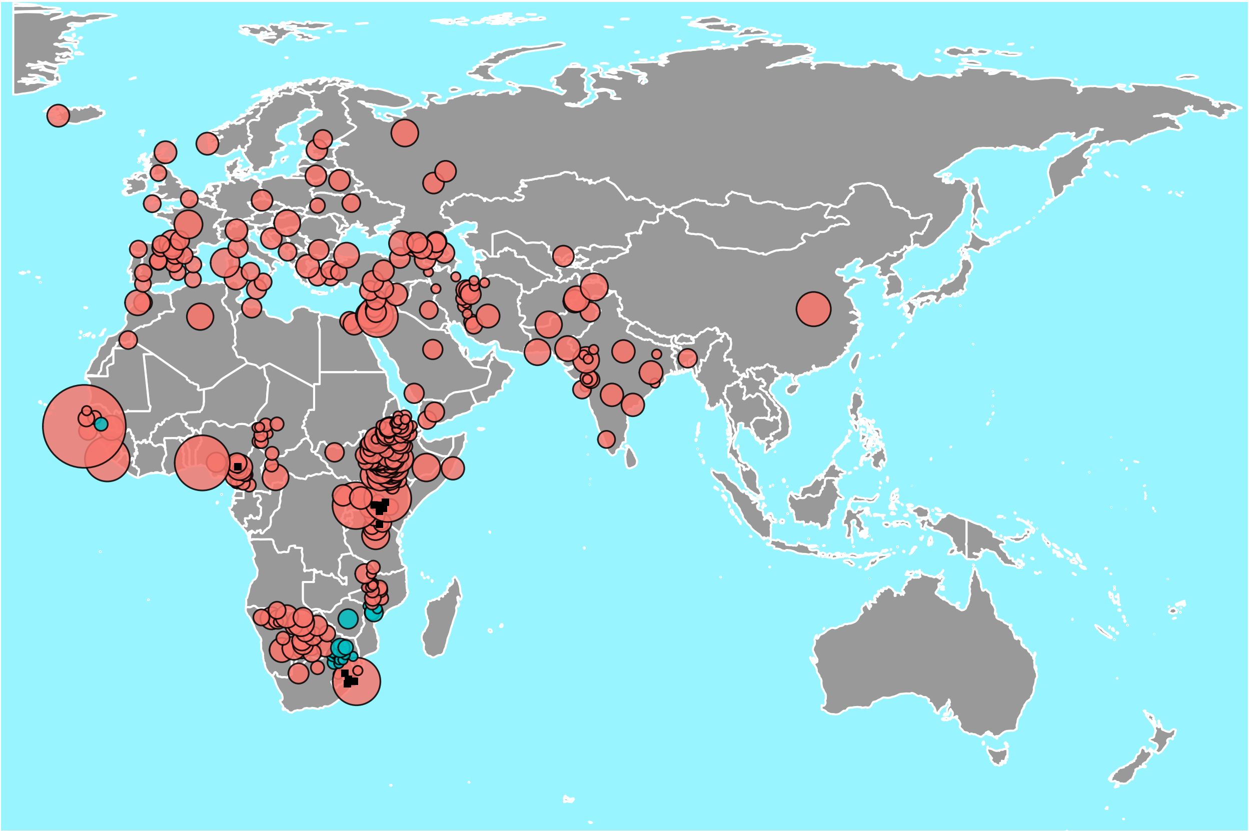

Fig. S2.

Map of previously published populations merged with the dataset (pink, n=3866, Data S2) and newly reported data genotyped for this study sampled from Mozambique, South Africa and Zimbabwe (blue, n=54). This excludes newly reported data from Cameroon, Republic of the Congo, Ghana, Nigeria and Sudan (n=1333, Fig. 1, Data S1). Two of the individuals sampled in South Africa had a birthplace in Senegal. Ancient individuals are shown with a black square. Size of point corresponds with population size (range is 1-100, mean=12). Modern-day samples are from Byrska-Bishop et al. (*43*), Fan et al. (*6*), Gurdasani et al. (*4*), Lazaridis et al. (*41*), Lipson et al. (*12*), López et al. (*45*), López et al. (*8*), MalariaGEN (*44*), Skoglund et al. (*11*) and Zheng-Bradley et al. (*42*). The 20 ancient African individuals from Cameroon, Kenya, Tanzania and South Africa were published in Lipson et al. (*12*), Prendergast et al. (*13*) and Schlebusch et al. (*10*).

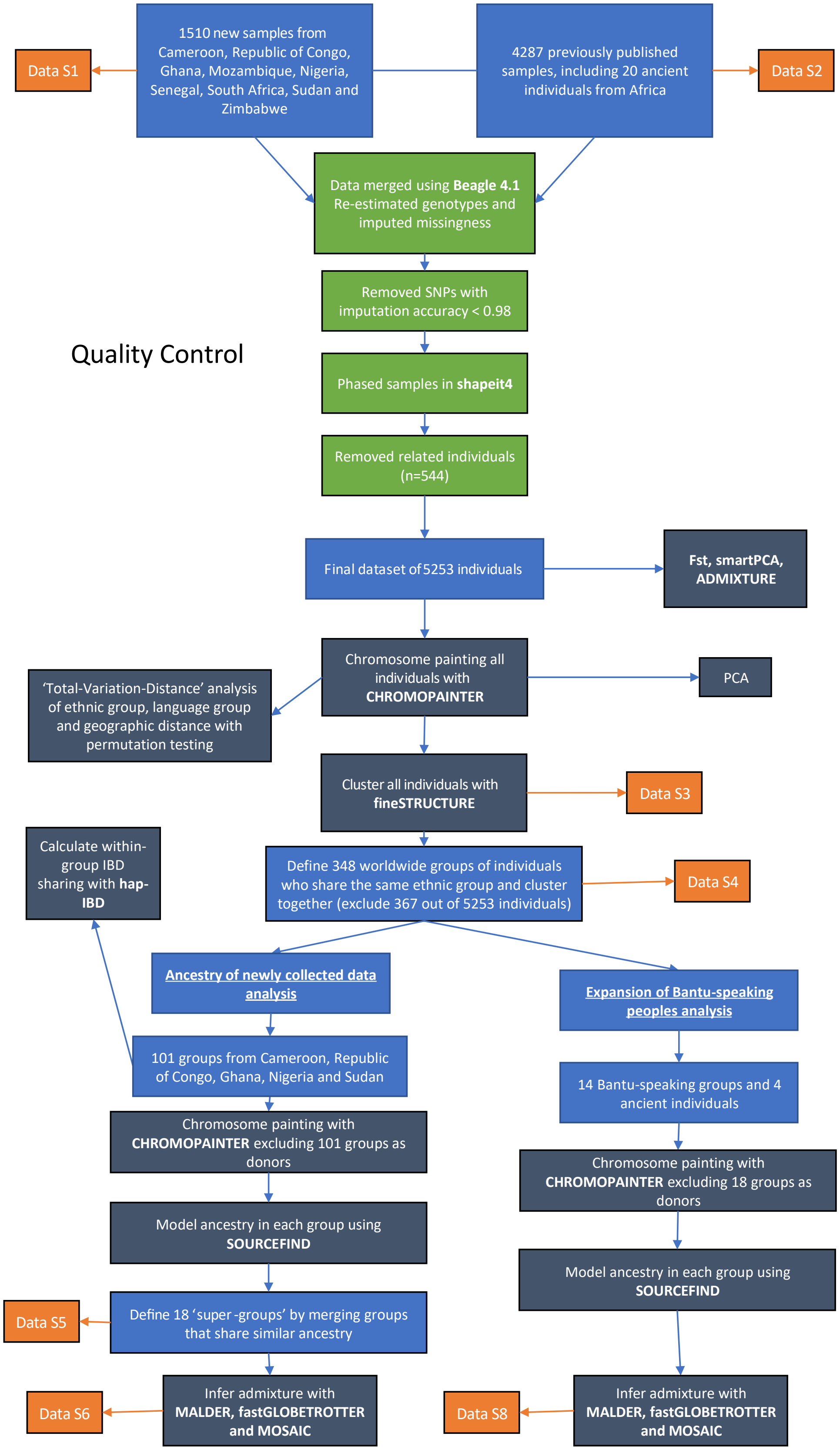
Data S1
1510 new samples from Cameroon, Republic of Congo, Ghana, Mozambique, Nigeria, Senegal, South Africa, Sudan and Zimbabwe
4287 previously published samples, including 20 ancient individuals from Africa
Data S2
Data merged using Beagle 4.1 Re-estimated genotypes and imputed missingness
Removed SNPs with imputation accuracy < 0.98
Quality Control
Phased samples in shapeit4
Removed related individuals (n=544)
Final dataset of 5253 individuals
Fst, smartPCA, ADMIXTURE
Chromosome painting all individuals with CHROMOPAINTER
PCA
'Total-Variation-Distance' analysis of ethnic group, language group and geographic distance with permutation testing
Cluster all individuals with fineSTRUCTURE
Data S3
Calculate within-group IBD sharing with hap-IBD
Define 348 worldwide groups of individuals who share the same ethnic group and cluster together (exclude 367 out of 5253 individuals)
Data S4
Ancestry of newly collected data analysis
Expansion of Bantu-speaking peoples analysis
101 groups from Cameroon, Republic of Congo, Ghana, Nigeria and Sudan
14 Bantu-speaking groups and 4 ancient individuals
Chromosome painting with CHROMOPAINTER excluding 101 groups as donors
Chromosome painting with CHROMOPAINTER excluding 18 groups as donors
Model ancestry in each group using SOURCEFIND
Model ancestry in each group using SOURCEFIND
Data S5
Define 18 'super-groups' by merging groups that share similar ancestry
Data S6
Infer admixture with MALDER, fastGLOBETROTTER and MOSAIC
Data S8
Infer admixture with MALDER, fastGLOBETROTTER and MOSAIC

Fig. S3.

Flowchart of the analysis process. Blue boxes indicate dataset/analysis descriptions, green boxes show quality control steps, grey boxes show analysis steps, and orange boxes indicate where data is reported.

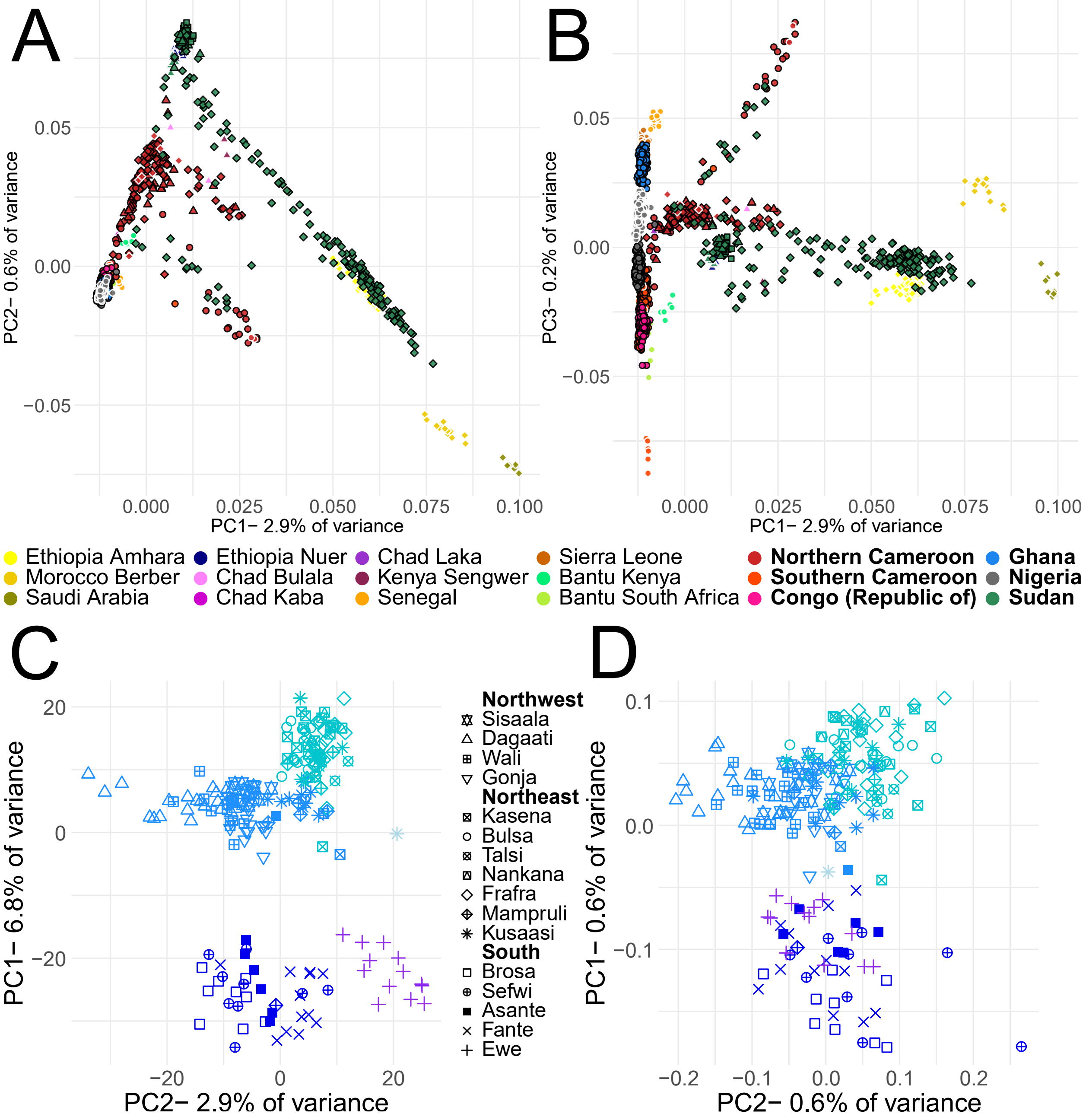

Fig. S4.

Comparing principal component analyses calculated either using smartPCA or from the ChromoPainter output (Methods). (A) and (B) principal component analysis (PCA) using the same samples as Fig. 2, but calculated using each SNP independently with smartPCA, rather than with haplotypes. (C) and (D) PCAs of all samples from Ghana, where shape corresponds to ethnic group and colour corresponds to fineSTRUCTURE cluster (see Fig. 3-4). In (C) PCs were generated using haplotypes, while (D) uses smartPCA. By using haplotypes, as in (C), there is increased power to differentiate between eastern and western clusters in Ghana.

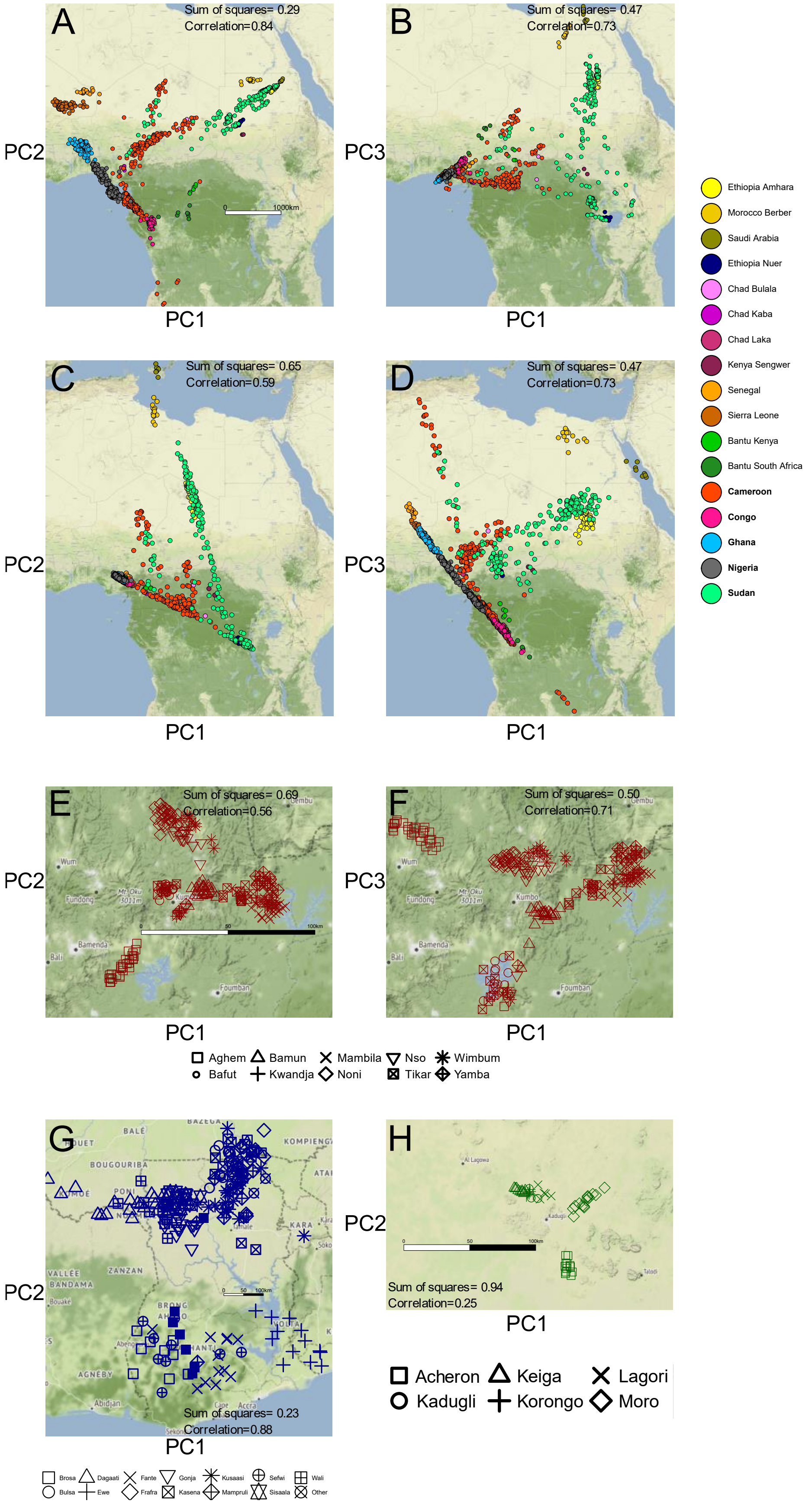
A
Sum of squares= 0.29
Correlation=0.84
PC2
PC1
B
Sum of squares= 0.47
Correlation=0.73
PC3
PC1
C
Sum of squares= 0.65
Correlation=0.59
PC2
PC1
D
Sum of squares= 0.47
Correlation=0.73
PC3
PC1
Ethiopia Amhara
Morocco Berber
Saudi Arabia
Ethiopia Nuer
Chad Bulala
Chad Kaba
Chad Laka
Kenya Sengwer
Senegal
Sierra Leone
Bantu Kenya
Bantu South Africa
Cameroon
Congo
Ghana
Nigeria
Sudan
E
Sum of squares= 0.69
Correlation=0.56
PC2
PC1
F
Sum of squares= 0.50
Correlation=0.71
PC3
PC1
Aghem
Bamun
Mambila
Nso
Wimbum
Bafut
Kwandja
Noni
Tikar
Yamba
G
Sum of squares= 0.23
Correlation=0.88
PC2
PC1
Brosa
Dagaati
Fante
Gonja
Kusaasi
Sefwi
Wali
Bulsa
Ewe
Frafra
Kasena
Mampruli
Sisaala
Other
H
Sum of squares= 0.94
Correlation=0.25
PC2
PC1
Acheron
Keiga
Lagori
Kadugli
Korongo
Moro

Fig. S5.
Procrustes analyses inferring the correlation between principal components and geography. (A) and (B) Procrustes analyses of the haplotype-based PCA in Fig. 2, (C) and (D) Procrustes analyses of the smartPCA PCA in Fig. S4, all showing the rotated PCs over a map, and the sum of squares and the Pearson correlation between PCs and the mean coordinates of each individual. For PC2 and PC3, both the haplotype-based PCA and smartPCA do equally well. However, for PC1 and PC2, the haplotype-based PCA has a much stronger correlation with geography.
(E), (F), (G) and (H) show Procrustes analyses for the regions shown in Fig. 4, using a haplotype-based PCA. For the Grassfields, correlation with PC3 is better than PC2. Ghana shows a very high correlation between PCs and geography, while the South Kordofan region of Sudan has a low correlation.

See separate pdf.

Fig. S6.
ADMIXTURE analysis from K=2 to K=9 of the newly reported samples from Cameroon, Republic of the Congo, Ghana, Nigeria and Sudan. Reference populations on the left include: Norwegian, Orcadian, Palestinian, Morocco Berber, Ethiopia Amhara, Ethiopia Nuer, Senegal Mandinka, Kenya Bantu, South Africa Bantu and Mbuti Pygmy. The lowest cross validation error was at K=7.

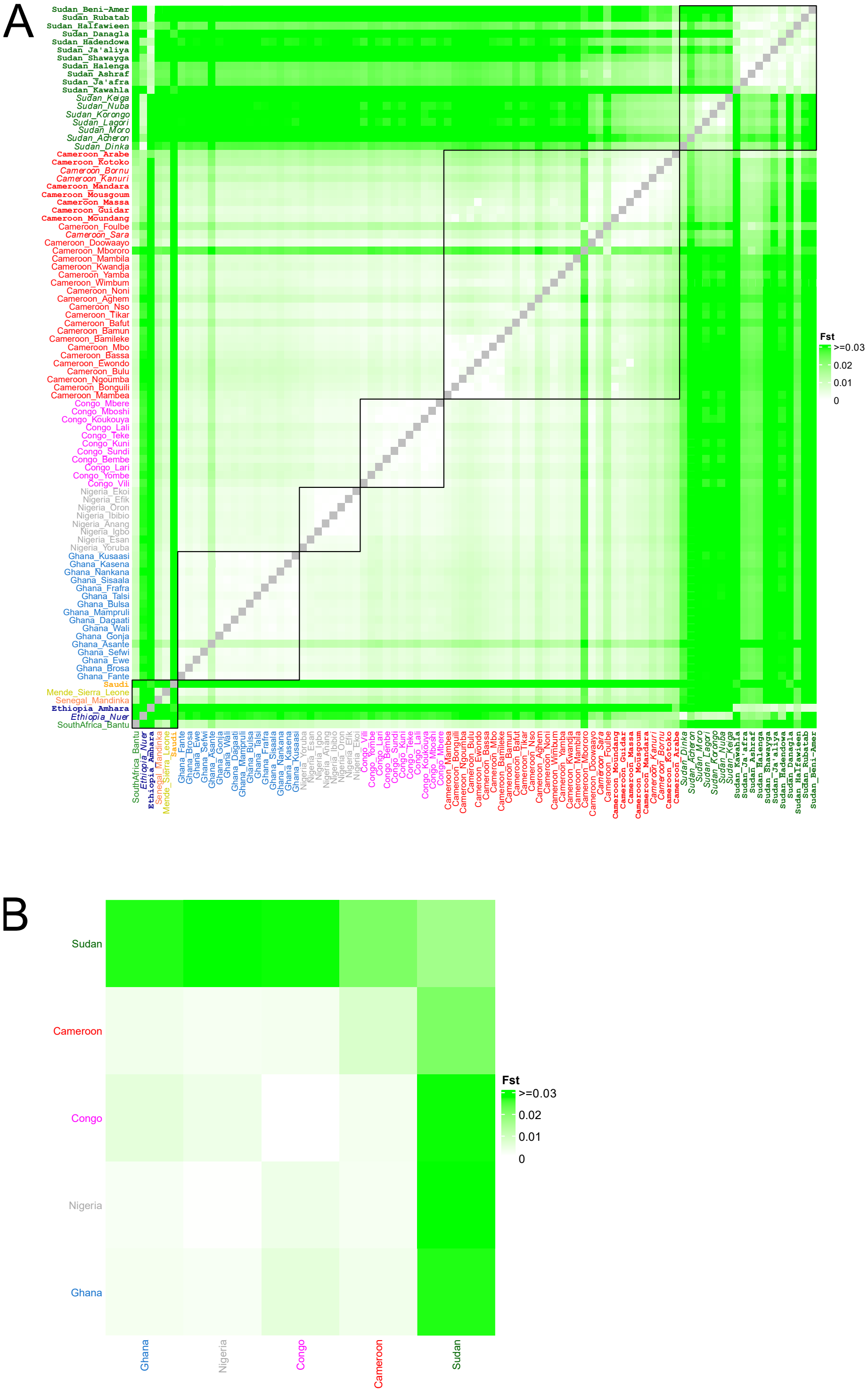

Fig. S7.
Heatmaps showing the average genetic similarity (F_{st}) between (A) every pair of ethnic groups sampled from Cameroon, Republic of the Congo, Ghana, Nigeria and Sudan, as well as populations included in the PCA in Fig. 2 and (B) each country. Ethnic groups in bold speak Afro-Asiatic languages, and those in italics speak Nilo-Saharan or Kordofanian languages. All other groups speak a Niger-Congo language.

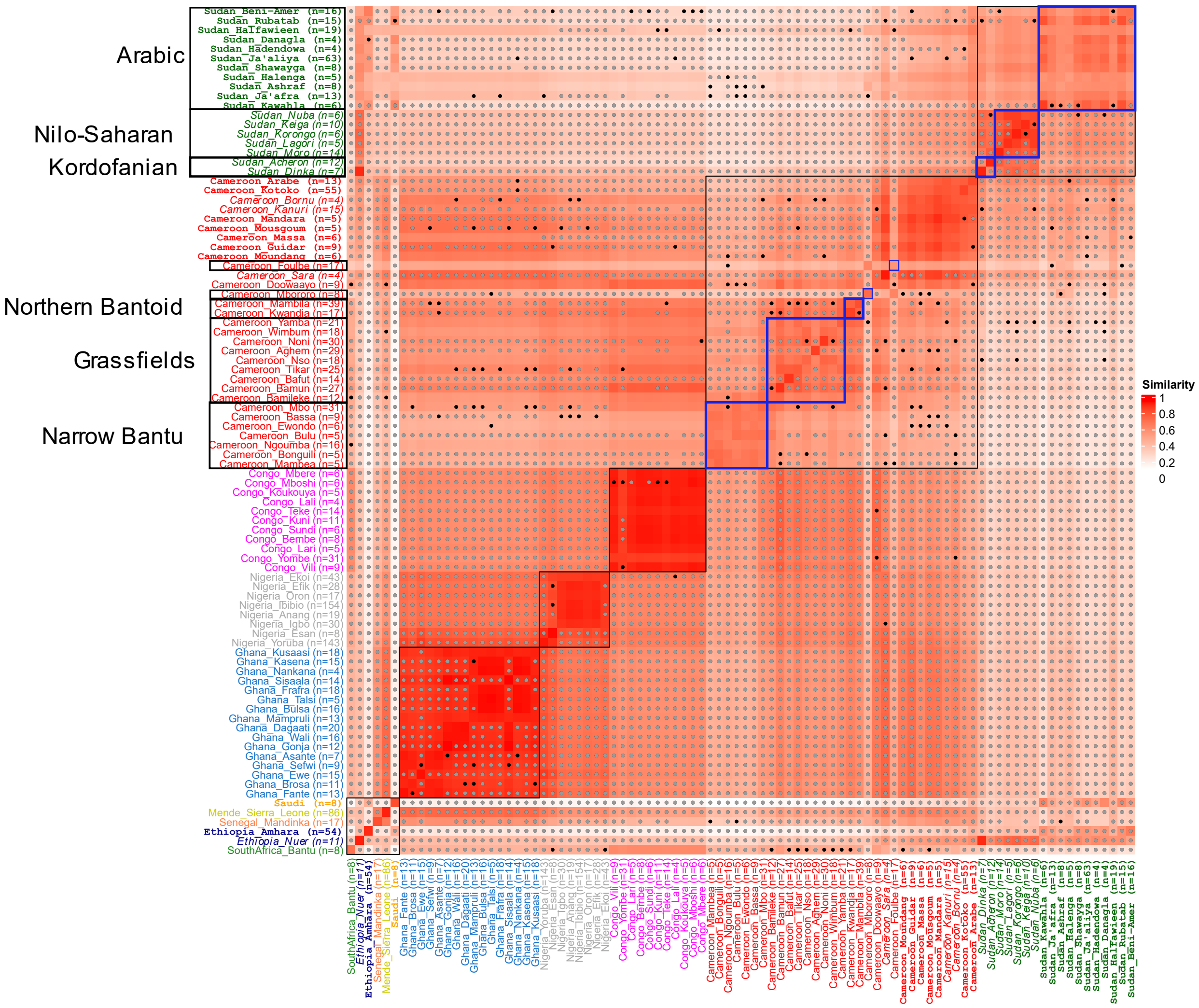

Fig. S8.

Heatmap showing the average genetic similarity (calculated as 1- TVD, Methods) between pairs of individuals from every pairing of ethnic groups sampled from Cameroon, Republic of the Congo, Ghana, Nigeria and Sudan, as well as populations included in the PCA in Fig. 2. Ethnic groups in bold speak Afro-Asiatic languages, and those in italics speak Nilo-Saharan or Kordofanian languages. All other groups speak a Niger-Congo language. Dots denote pairs of groups for which we can reject the null hypothesis that individuals from group A (rows) are more genetically similar to each other than individuals from A are to those from B (columns) at a per-test threshold of p<0.01 (black) or p<0.001 (grey) (Methods). These tests are not symmetric; group A can be distinguishable from group B while group B is not distinguishable from group A if (e.g.) group B has high genetic diversity. We conclude that the two groups can be distinguished if there is significance in either direction.

Annotations have been added to demonstrate certain results, with language groups shown at the sides and blue boxes placed around ethnic groups that speak that language. Firstly, there is low genetic similarity both within and between Arabic-speaking Sudanese groups, with only some ethnic groups being differentiable. This is in contrast with Nilo-Saharan and Kordofanian-

speaking Sudanese who have a higher genetic similarity within language group and many differentiable ethnic groups.

Next, both Fulani ethnic groups (self-described as Foulbe and Mbororo) are distinguishable from almost all other ethnic groups, while there is very little structure in the rest of northern Cameroon.

In southern Cameroon, the three main language groups (northern Bantoid, Grassfields and Narrow Bantu) are highlighted. Many ethnic groups are differentiable within northern Bantoid and Grassfields speakers, while Narrow Bantu speaker have only one pair of ethnic groups that are differentiable and a relatively low genetic similarity within and between ethnic groups.

Lastly, Ghana show relatively high genetic similarity among groups and low genetic similarity with groups outside Ghana. In Nigeria, structure is mostly present between western (Yoruba and Esan) and eastern ethnic groups.

Note: Some individuals self-described as Tikar speak a Grassfields language and some speak a Northern Bantoid language.

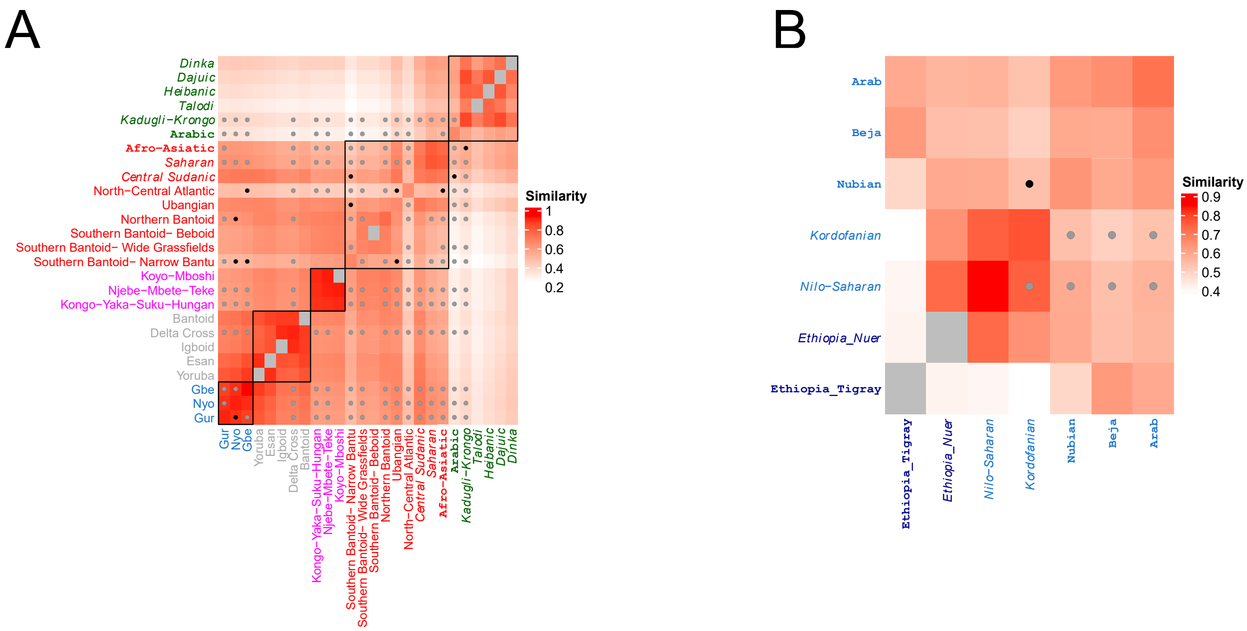

Fig. S9.

Heatmaps showing the average genetic similarity (calculated as 1- TVD) between pairs of individuals from different language classifications. The language classifications in (A) are given in Data S1, and described in more detail in Text S1. (B) shows language classifications within Sudan, grouping the languages of individuals from south Kordofan into Kordofanian and Nilo-Saharan. Sudanese Arabic speakers are classified into three ethnicities (Arab, Beja, Nubian) based on their pre-Arabic language (see Hollfelder et al. (*16*)). Ethiopian Nilo-Saharan and Afro-Asiatic speaking ethnic groups are included for reference. Languages in bold are Afro-Asiatic, and those in italics are Nilo-Saharan or Kordofanian. All other groups speak a Niger-Congo language. Dots denote pairs of groups for which we can reject the null hypothesis that individuals from group A (rows) are more genetically similar to each other than individuals from A are to those from B (columns) at a per-test threshold of $p<0.01$ (black) or $p<0.001$ (grey) (Methods). These tests are not symmetric; group A can be distinguishable from group B while group B is not distinguishable from group A if (e.g.) group B has high genetic diversity. We conclude that the two groups can be distinguished if there is significance in either direction. In the case where a language group comprised a single ethnic group, TVD could not be calculated as only comparisons between different ethnic groups were included (grey squares, see Methods). In these cases, permutation tests could not be carried out.

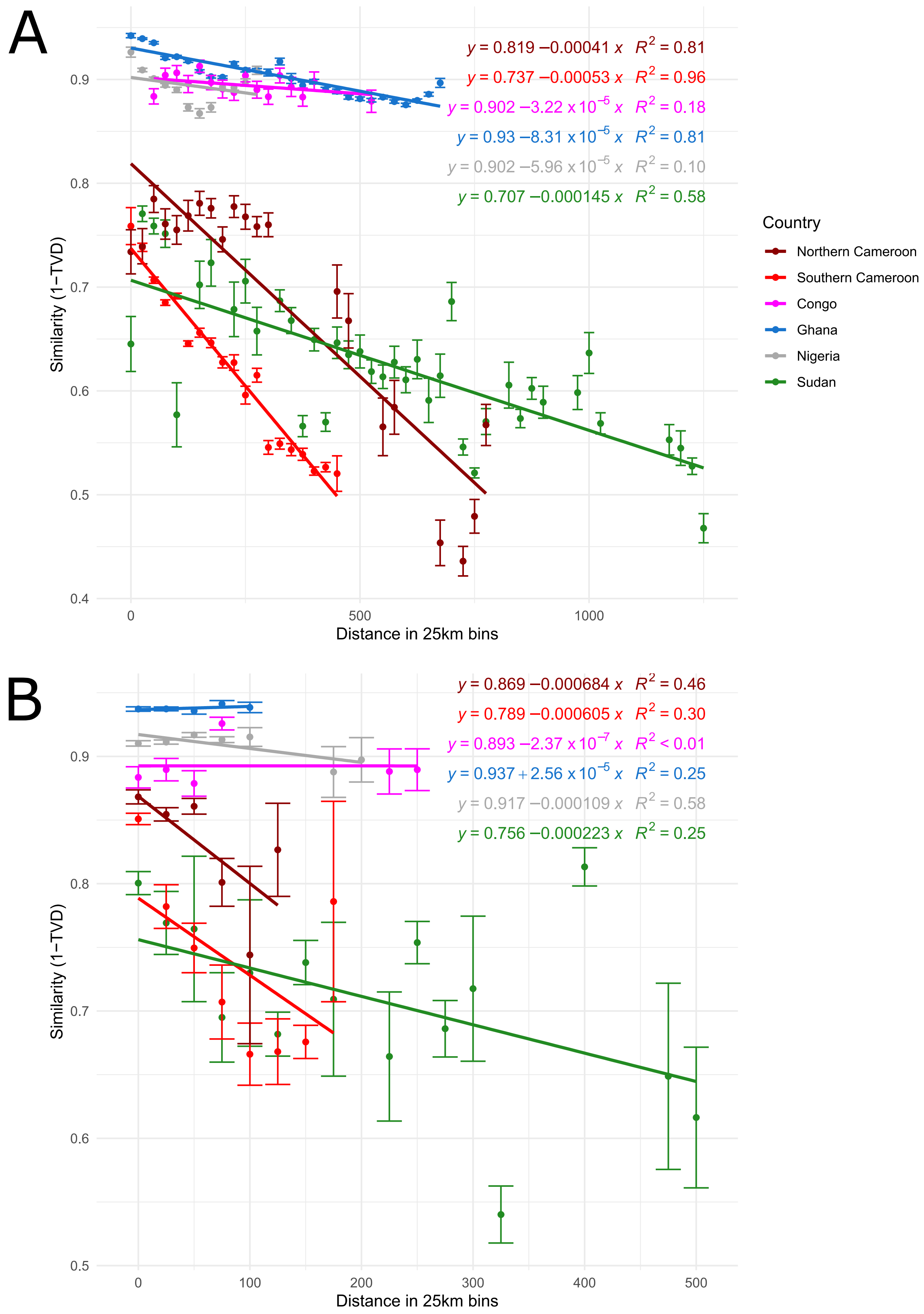
A
y = 0.819 −0.00041 x R^2 = 0.81
y = 0.737 −0.00053 x R^2 = 0.96
y = 0.902 −3.22 x 10^−5 x R^2 = 0.18
y = 0.93 −8.31 x 10^−5 x R^2 = 0.81
y = 0.902 −5.96 x 10^−5 x R^2 = 0.10
y = 0.707 −0.000145 x R^2 = 0.58
Similarity (1−TVD)
Distance in 25km bins
Country
Northern Cameroon
Southern Cameroon
Congo
Ghana
Nigeria
Sudan
B
y = 0.869 −0.000684 x R^2 = 0.46
y = 0.789 −0.000605 x R^2 = 0.30
y = 0.893 −2.37 x 10^−7 x R^2 < 0.01
y = 0.937 + 2.56 x 10^−5 x R^2 = 0.25
y = 0.917 −0.000109 x R^2 = 0.58
y = 0.756 −0.000223 x R^2 = 0.25
Similarity (1−TVD)
Distance in 25km bins

Fig. S10.

Plots of genetic similarity (1-TVD) among individuals pairs against distance (km) in 25km bins, split by country. Cameroonian individuals are split into north and south based on fineSTRUCTURE results and geography (see Fig. 1 and Fig. 3). (A) considers only individual pairings from different ethnic groups, while (B) considers only pairs of individuals from the same ethnic group. The analysis in (A) removes ethnic groups effects, as individuals of the same ethnic group are likely to have high similarity and live at short distances from one another. Hence this gives us a more conservative estimate of an isolation by distance effect. The analysis in (B) reveals that isolation by distance can still be seen in some cases even amongst individuals of the same ethnic groups living at different distances from one another. Individuals whose maternal grandmother and paternal grandfather lived more than 150km apart were removed for this analysis. For (A) only bins with more than 100 comparisons were included; for (B) this threshold was 20. Both plots have been filtered to remove bins where all comparisons involve one particular individual.

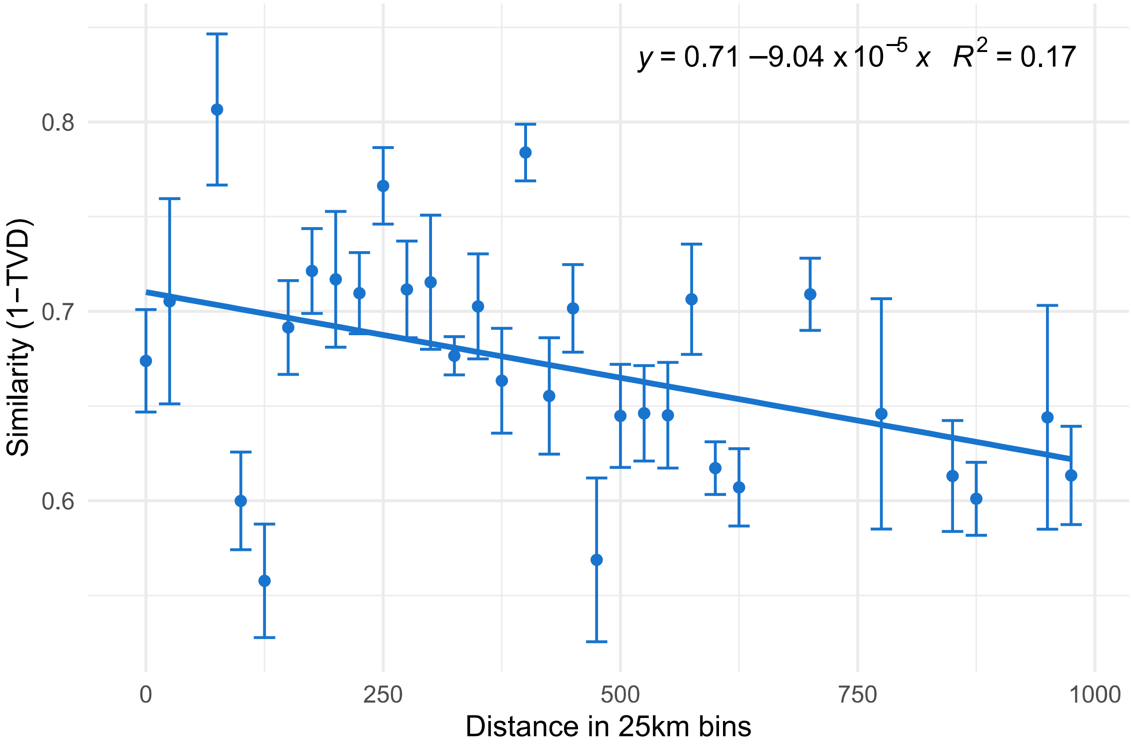

Fig. S11.

Plots of genetic similarity (1-TVD "internal" analysis) against distance (km) in 25km bins between each pair of individuals from different ethnic groups who live on the Nile in Sudan (i.e. excluding south Kordofan and coastal individuals). There is a weak negative correlation between geographic distance and genetic similarity. Individuals whose maternal grandmother and paternal grandfather lived more than 150km apart were removed for this analysis. Only bins with more than 20 comparisons were included and bins where all comparisons involve one particular individual were removed.

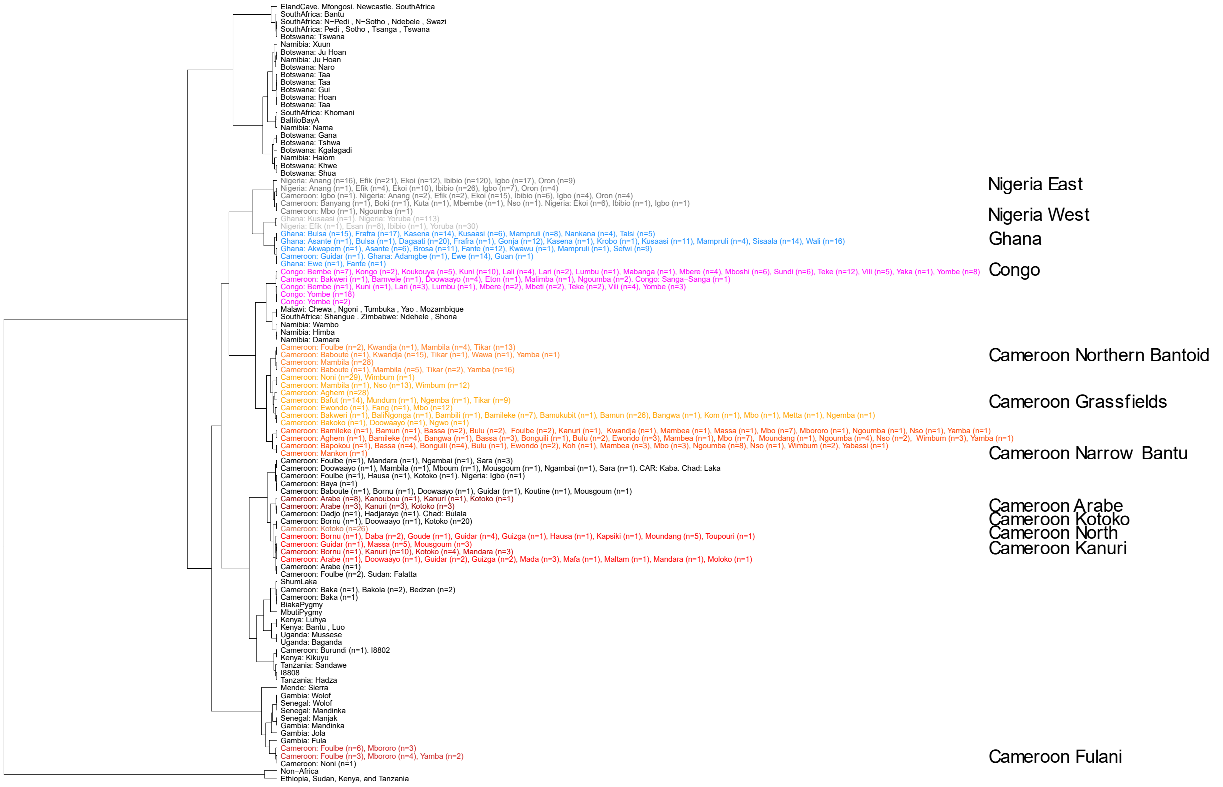

Fig. S12.

fineSTRUCTURE dendrogram of the whole dataset with populations from Cameroon (orange to red), Republic of the Congo (pink), Ghana (blue), or Nigeria (grey) clustered, and the rest of the dataset grouped into 'superindividuals' (Methods). The ethnic group composition of each cluster is displayed (and can be found in Data S3). This tree was used, along with information on self-reported ethnic group, to cluster individuals from Cameroon, Republic of the Congo, Ghana, and Nigeria (Methods). The super-groups used for admixture analyses are shown in different colours, corresponding to those in Fig. 3 (Data S5).

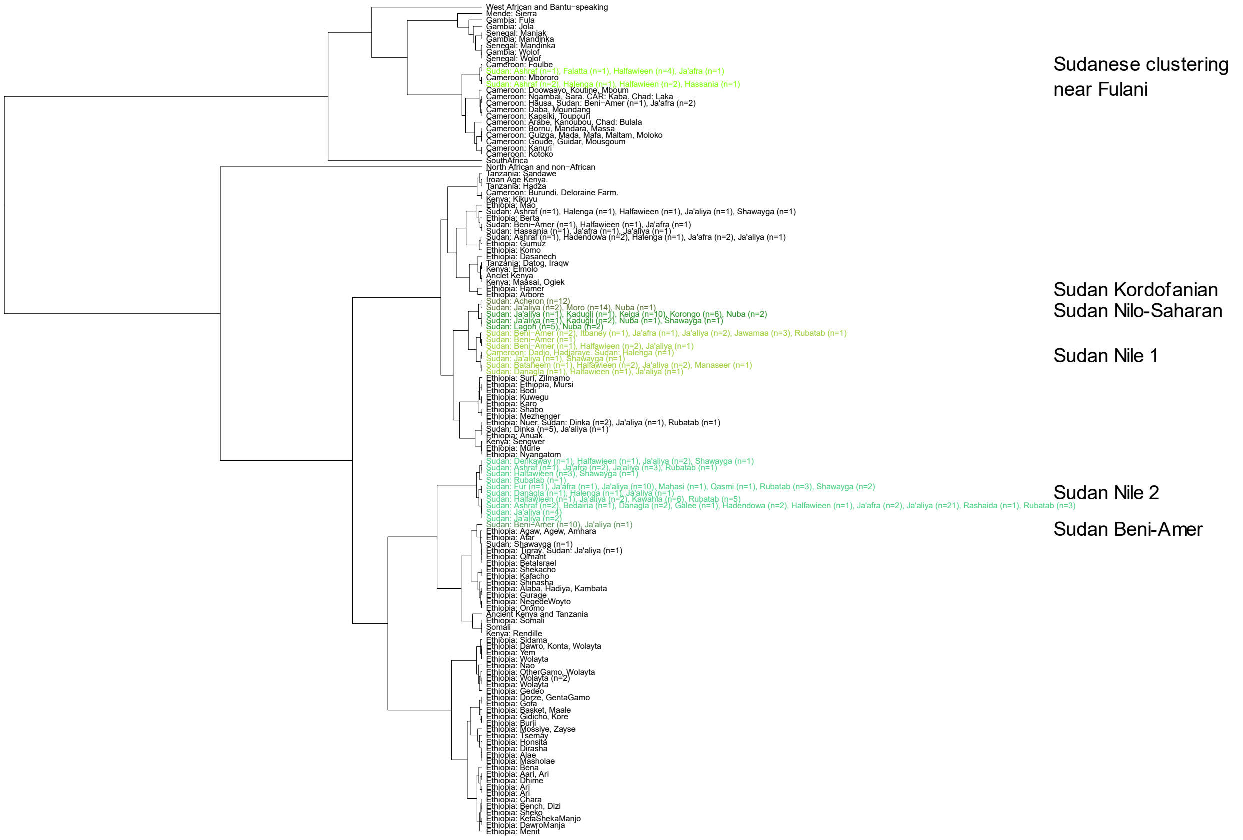

Fig. S13.
fineSTRUCTURE dendrogram of Sudanese individuals, with the rest of the dataset clustered into 'superindividuals' (Methods). The ethnic group composition of each cluster is displayed (and can be found in Data S3). This tree was used, along with information on self-reported ethnic group, to cluster individuals from Sudan (Methods). The five Sudanese 'super-groups' are highlighted in different shades of green starting from the bottom; Beni-Amer, Nile2, Nile1, Nilo-Saharan and Kordofanian. The final group at the top shows the Sudanese individuals who cluster with the Fulani from Cameroon. Differential admixture between individuals, as demonstrated in Fig. S15, is likely driving these clustering results.

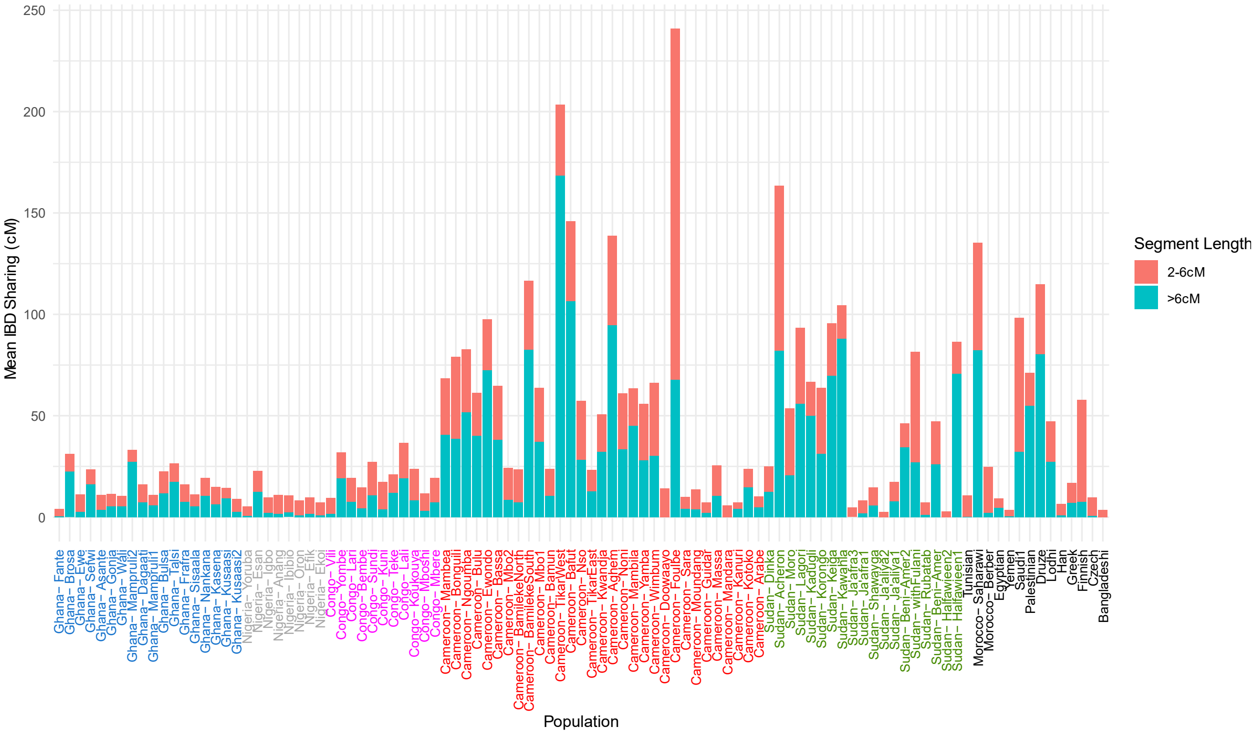

Fig. S14.

Mean IBD sharing between each pair of individuals within each cluster from Ghana, Nigeria, Republic of the Congo, Cameroon and Sudan, as well as a selection of North African and non-African clusters (black label). All clusters contain more than 3 individuals. Total IBD length has been divided into short (2-6cM) and long (>6cM) tracts.

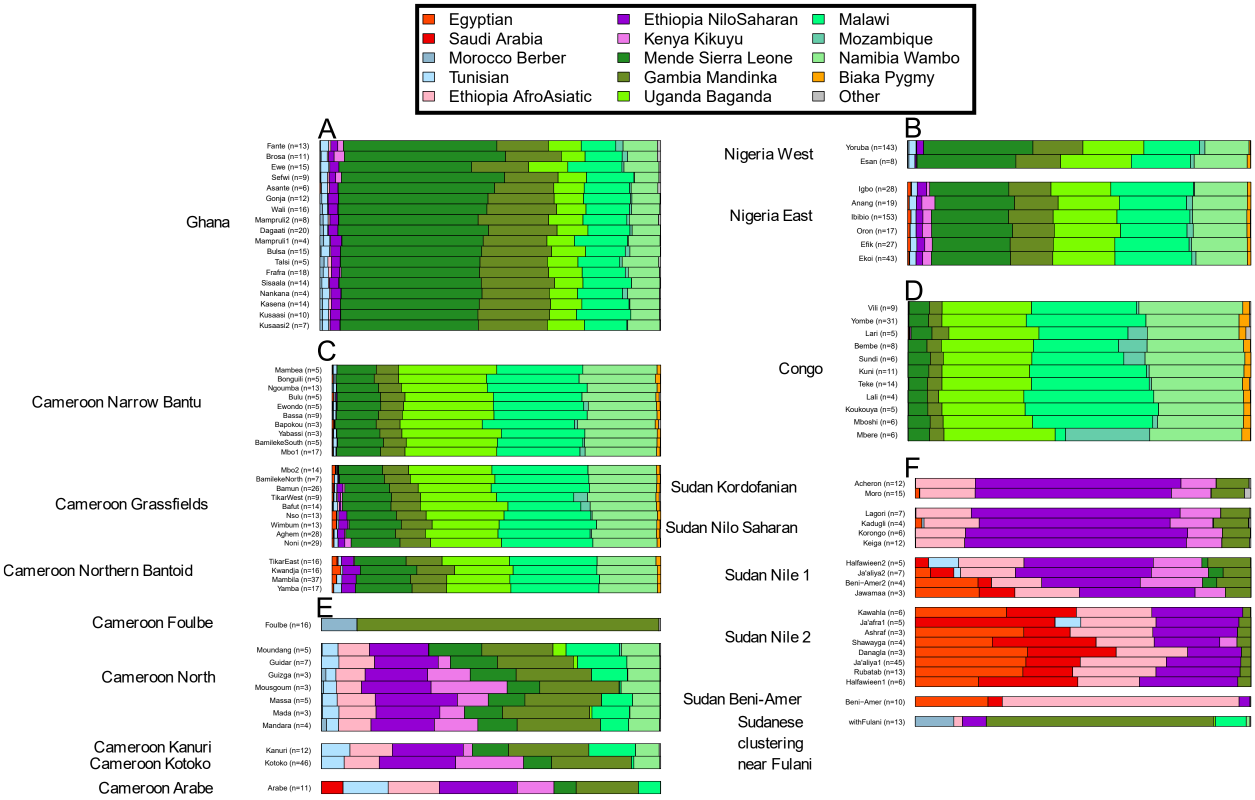

Fig. S15.

Ancestry modeled in SOURCEFIND for each cluster from Ghana (A), Nigeria (B), Cameroon (C) and (E), Republic of the Congo (D) and Sudan (F). Clusters are grouped into ‘super-groups’ as shown on the fineSTRUCTURE dendrograms in Fig. S12 and 13. Clusters within each super-group exhibit similar patterns of inferred recent ancestry sharing with reference populations. Reference populations are shown in the key and follow red=Arabic, blue=north African, pink=east African, green=west African and Bantu-speaking peoples, orange=rainforest hunter-gatherer. Note that the reference populations are themselves admixed, and so results should be interpreted with caution.

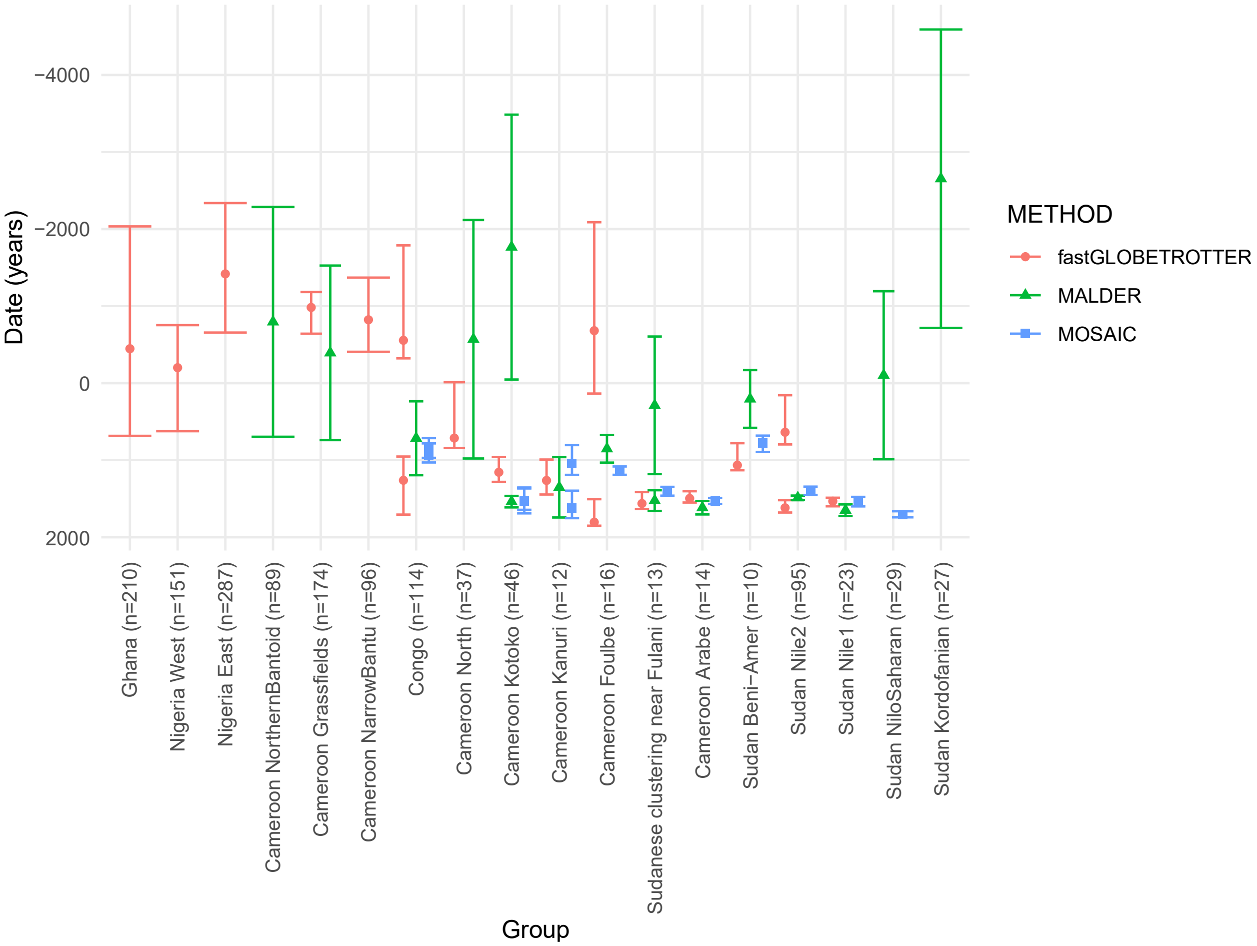

Fig. S16.
Admixture dates and their 95% confidence intervals inferred using fastGLOBETROTTER (red circle), MALDER (green triangle) and MOSAIC (blue square) for the 18 "super-groups" from Cameroon, Republic of the Congo, Ghana, Nigeria and Sudan. See Data S6 for inferred admixture sources. Inferred dates overlap for at least two methods in 10 out of 18 cases. We note that our sample sizes vary widely, which will impact our power to detect and date admixture.

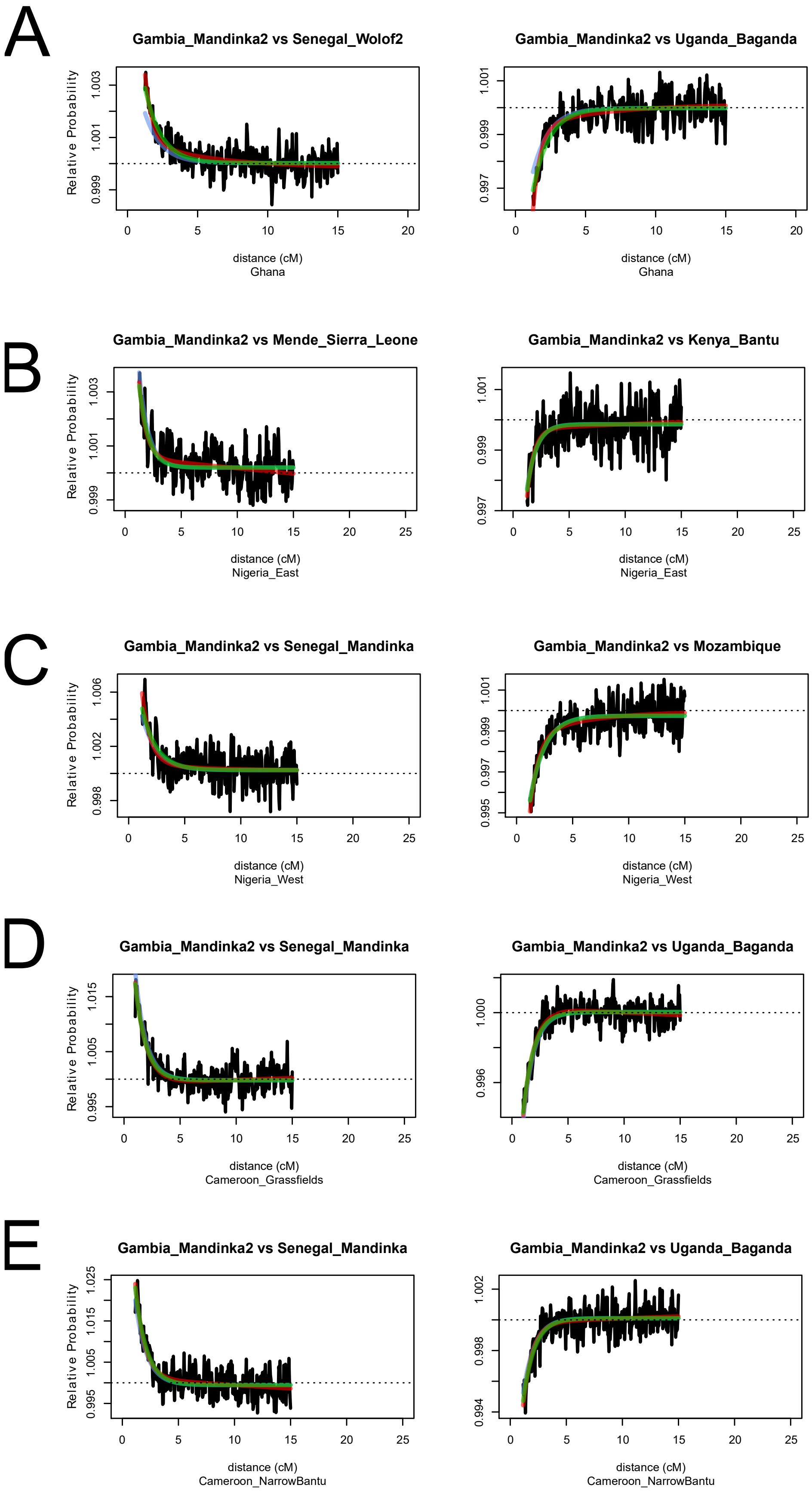
A
Gambia_Mandinka2 vs Senegal_Wolof2
Relative Probability
distance (cM)
Ghana
Gambia_Mandinka2 vs Uganda_Baganda
distance (cM)
Ghana
B
Gambia_Mandinka2 vs Mende_Sierra_Leone
Relative Probability
distance (cM)
Nigeria_East
Gambia_Mandinka2 vs Kenya_Bantu
distance (cM)
Nigeria_East
C
Gambia_Mandinka2 vs Senegal_Mandinka
Relative Probability
distance (cM)
Nigeria_West
Gambia_Mandinka2 vs Mozambique
distance (cM)
Nigeria_West
D
Gambia_Mandinka2 vs Senegal_Mandinka
Relative Probability
distance (cM)
Cameroon_Grassfields
Gambia_Mandinka2 vs Uganda_Baganda
distance (cM)
Cameroon_Grassfields
E
Gambia_Mandinka2 vs Senegal_Mandinka
Relative Probability
distance (cM)
Cameroon_NarrowBantu
Gambia_Mandinka2 vs Uganda_Baganda
distance (cM)
Cameroon_NarrowBantu

Fig. S17.
Example fastGLOBETROTTER coancestry curves for the admixture event inferred in (A) Ghana, (B) Nigeria East, (C) Nigeria West, (D) Cameroon Grassfields and (E) Cameroon Narrow Bantu. The black line depicts the (scaled) inferred probability that two DNA segments separated by distance X in a target population individual were inherited from sources related to (respectively) the two reference populations given in the plot's title. Curves decreasing with distance indicate the two reference populations represent the same admixing source, while increasing curves indicate the two reference populations represent different admixing sources. The green and red lines show the model fit assuming one or two pulses of admixture, respectively.

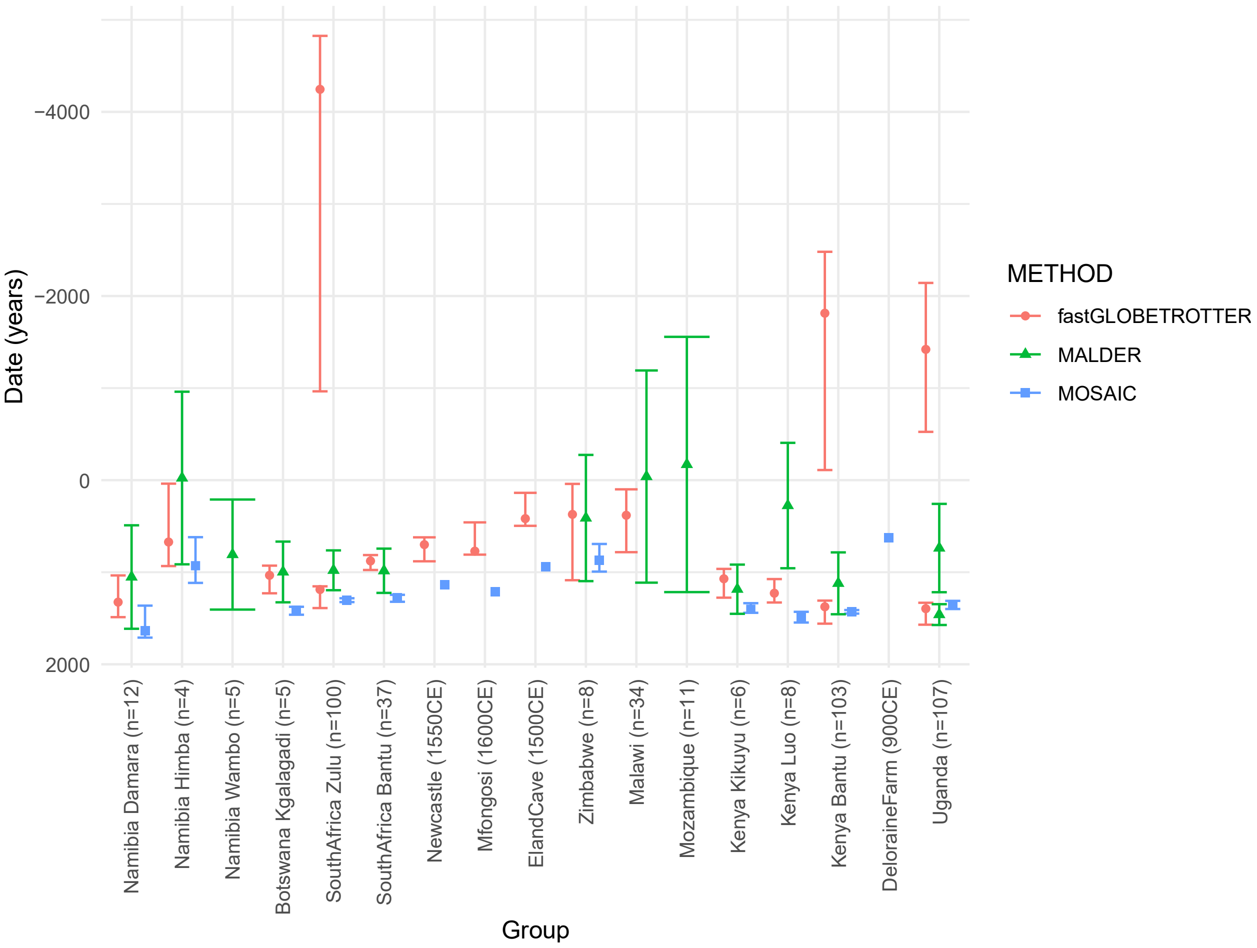

Fig. S18.

Inferred admixture dates and their 95% confidence intervals inferred using fastGLOBETROTTER (red circle), MALDER (green triangle) and MOSAIC (blue square) for the 13 groups and 4 ancient individuals with Bantu speaking-related ancestry. Dates for the ancient individuals have been adjusted using their carbon dates (Deloraine Farm 900CE, Newcastle 1550CE, Mfongosi 1600CE, Eland Cave 1500CE). MALDER cannot infer admixture dates using a single sample, and MOSAIC cannot infer date confidence intervals in such cases, so these results are not shown for the ancient individuals. See Data S8 for inferred admixture sources. Inferred dates overlap for at least two methods in 10 out of 13 cases. We note that sample sizes, shown in the axis labels, vary widely, which means power to infer admixture also varies widely. In groups with very large sample sizes (Zulu, Uganda, Kenya Bantu), multiple dates of admixture are inferred, potentially because of the increased power. Hence this means that in other groups, we may simply lack the power to detect additional admixture events.

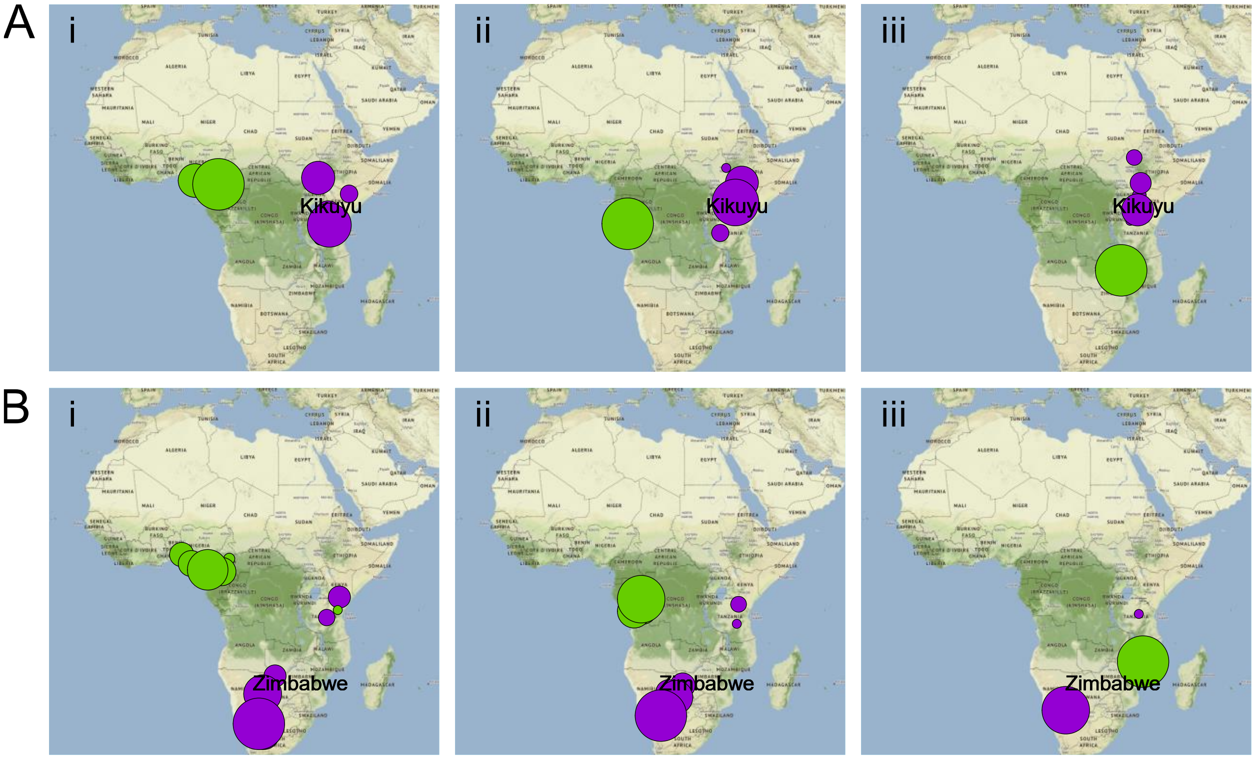

Fig. S19.

fastGLOBETROTTER results for (A) the Kikuyu and (B) Zimbabweans, when using non Bantu-speaking groups as surrogates plus (i) nothing else, (ii) Congolese Bantu speakers, and (iii) all Bantu-speakers in the dataset. This is analogous to analyses in (*5*, *15*, *23*, *30*)). The inferred Bantu-speaking-like admixing source is represented with green, and the other inferred admixing source is represented with purple. Size of point indicates the percentage contribution of that surrogate population to describe the genetic make-up each admixing source. These results provide evidence for the late split model, as Bantu-speaking-related ancestry (green) is more closely related to Congolese than Cameroonians, and more closely related to Malawians and Mozambicans than Congolese, consistent with the migration being first southwards and eastwards before splitting into two branches.

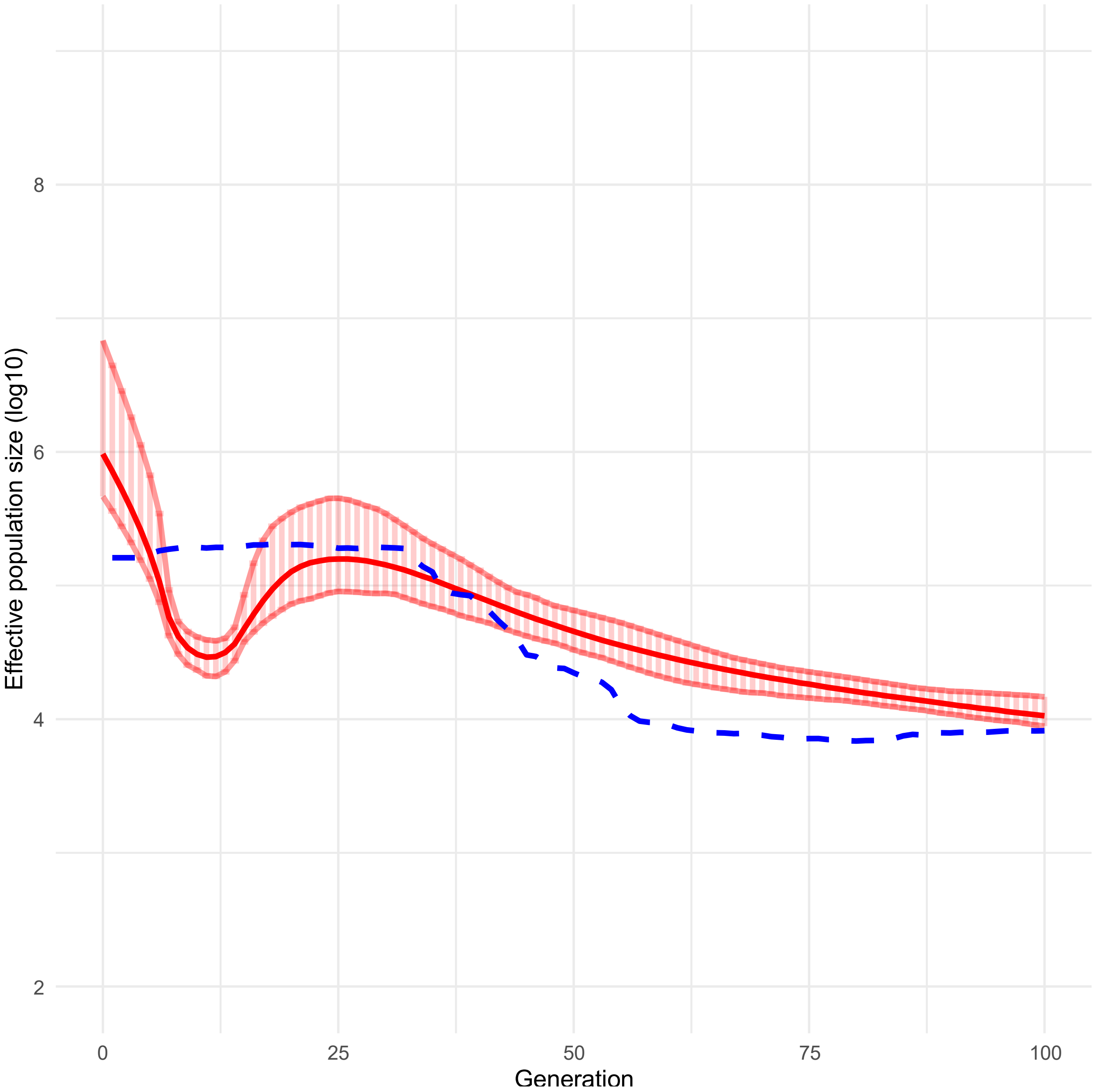

Fig. S20.
Effective population size (N_e) changes in the history of individuals from the Republic of the Congo, inferred using GONE (blue dotted) and IBDNe (red). IBDNe 95% confidence intervals are shown as transparent red lines. Results are shown for up to 100 generations ago, as inference any earlier than this can be unreliable.

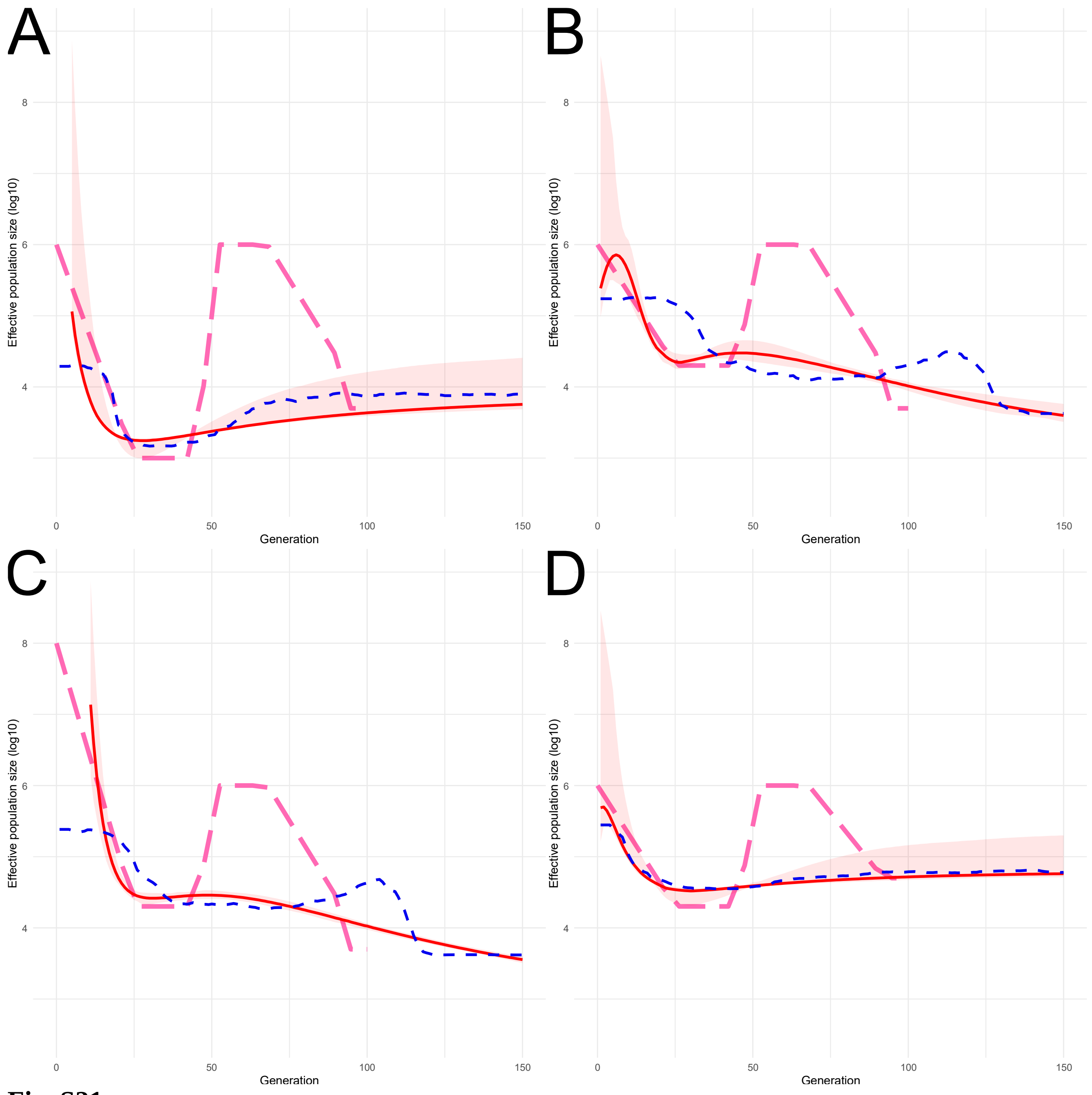

Fig. S21.

Simulations in msprime (Methods, text S4) of a population expansion, followed by a bottleneck, followed by an expansion, to mimic the proposed two wave expansion into the Congo basin. The four simulations (panels) vary by the strength of bottleneck and expansions, with the pink dashed line indicating the effective population size trajectory simulated. The red and blue dashed lines depict inference using IBDNe (considering inferred IBD segments > 2cM) and GONE, respectively, with shading showing 95% confidence intervals for IBDNe. Only N_e values and 95% confidence intervals between 10^2.5 and 10^9 are shown. In all scenarios (A)-(D), neither IBDNe nor GONE are able to capture the true population dynamics, with IBDNe often inferring relatively constant population size changes until recently.

Data S1. (separate file)
Samples published in this study, including self-described ethnic group, two levels of language classification (as defined by glottolog and ethnologue), number of individuals from that population, and the mean latitude and longitude for all individuals in that population (see Fig. S1 for map).

Data S2. (separate file)
Previously published data included in this study (shown in Fig. S2), including both present day and ancient samples.

Data S3. (separate file)
Raw fineSTRUCTURE results, showing the population composition of each cluster and which analysis they were inferred in.

Data S4. (separate file)
Clusters defined using both fineSTRUCTURE results and each individuals' self-described ethnic group (Methods), with the final eight columns showing which analyses each cluster acted as a source in.

Data S5. (separate file)
Composition of the 18 "super-groups" formed by merging clusters up the fineSTRUCTURE dendrogram, used for admixture and ancestry analysis (Fig. 3 and 5).

Data S6. (separate file)
fastGLOBETROTTER, MOSAIC, MALDER and SOURCEFIND results for the 18 super-groups.

Data S7. (separate file)
fastGLOBETROTTER, MALDER and MOSAIC results for extra analyses of Sudanese clustering near Fulani to test specific hypothesis of admixture between Cameroonian populations.

Data S8. (separate file)
fastGLOBETROTTER, MOSAIC, MALDER and SOURCEFIND results for the 13 groups with Bantu-speaking related ancestry and 4 ancient individuals.

REFERENCES AND NOTES

1. S. A. Tishkoff, F. A. Reed, F. R. Friedlaender, C. Ehret, A. Ranciaro, A. Froment, J. B. Hirbo, A. A. Awomoyi, J. M. Bodo, O. Doumbo, M. Ibrahim, A. T. Juma, M. J. Kotze, G. Lema, J. H. Moore, H. Mortensen, T. B. Nyambo, S. A. Omar, K. Powell, G. S. Pretorius, M. W. Smith, M. A. Thera, C. Wambebe, J. L. Weber, S. M. Williams, The genetic structure and history of Africans and African Americans. *Science* **324**, 1035–1044 (2009).

2. M. C. Campbell, S. A. Tishkoff, African genetic diversity: Implications for human demographic history, modern human origins, and complex disease mapping. *Annu. Rev. Genomics Hum. Genet.* **9**, 403–433 (2008).

3. G. Hellenthal, N. Bird, S. Morris, Structure and ancestry patterns of Ethiopians in genome-wide autosomal DNA. *Hum. Mol. Genet.* **30**, R42–R48 (2021).

4. D. Gurdasani, T. Carstensen, F. Tekola-Ayele, L. Pagani, I. Tachmazidou, K. Hatzikotoulas, S. Karthikeyan, L. Iles, M. O. Pollard, A. Choudhury, G. R. S. Ritchie, Y. Xue, J. Asimit, R. N. Nsubuga, E. H. Young, C. Pomilla, K. Kivinen, K. Rockett, A. Kamali, A. P. Doumatey, G. Asiki, J. Seeley, F. Sisay-Joof, M. Jallow, S. Tollman, E. Mekonnen, R. Ekong, T. Oljira, N. Bradman, K. Bojang, M. Ramsay, A. Adeyemo, E. Bekele, A. Motala, S. A. Norris, F. Pirie, P. Kaleebu, D. Kwiatkowski, C. Tyler-Smith, C. Rotimi, E. Zeggini, M. S. Sandhu, The African genome variation project shapes medical genetics in Africa. *Nature* **517**, 327–332 (2015).

5. A. Choudhury, S. Aron, L. R. Botigué, D. Sengupta, G. Botha, T. Bensellak, G. Wells, J. Kumuthini, D. Shriner, Y. J. Fakim, A. W. Ghoorah, E. Dareng, T. Odia, O. Falola, E. Adebiyi, S. Hazelhurst, G. Mazandu, O. A. Nyangiri, M. Mbiyavanga, A. Benkahla, S. K. Kassim, N. Mulder, S. N. Adebamowo, E. R. Chimusa, D. Muzny, G. Metcalf, R. A. Gibbs, E. Matovu, B. Bucheton, C. Hertz-Fowler, M. Koffi, A. Macleod, D. Mumba-Ngoyi, H. Noyes, O. A. Nyangiri, G. Simo, M. Simuunza, C. Rotimi, M. Ramsay, A. Choudhury, S. Aron, L. Botigué, D. Sengupta, G. Botha, T. Bensellak, G. Wells, J. Kumuthini, D. Shriner, Y. J. Fakim, A. W. Ghoorah, E. Dareng, T. Odia, O. Falola, E. Adebiyi, S. Hazelhurst, G. Mazandu, O. A. Nyangiri, M. Mbiyavanga, A. Benkahla, S. K. Kassim, N. Mulder, S. N.

Adebamowo, E. R. Chimusa, C. Rotimi, M. Ramsay, A. A. Adeyemo, Z. Lombard, N. A. Hanchard, C. Adebamowo, G. Agongo, R. P. Boua, A. Oduro, H. Sorgho, G. Landouré, L. Cissé, S. Diarra, O. Samassékou, G. Anabwani, M. Matshaba, M. Joloba, A. Kekitiinwa, G. Mardon, S. W. Mpoloka, S. Kyobe, B. Mlotshwa, S. Mwesigwa, G. Retshabile, L. Williams, A. Wonkam, A. Moussa, D. Adu, A. Ojo, D. Burke, B. O. Salako, E. Matovu, B. Bucheton, C. Hertz-Fowler, M. Koffi, A. Macleod, D. Mumba-Ngoyi, H. Noyes, O. A. Nyangiri, G. Simo, M. Simuunza, P. Awadalla, V. Bruat, E. Gbeha, A. A. Adeyemo, Z. Lombard, N. A. Hanchard, High-depth African genomes inform human migration and health. *Nature* **586**, 741–748 (2020).

6. S. Fan, D. E. Kelly, M. H. Beltrame, M. E. B. Hansen, S. Mallick, A. Ranciaro, J. Hirbo, S. Thompson, W. Beggs, T. Nyambo, S. A. Omar, D. W. Meskel, G. Belay, A. Froment, N. Patterson, D. Reich, S. A. Tishkoff, African evolutionary history inferred from whole genome sequence data of 44 indigenous African populations. *Genome Biol.* **20**, 82 (2019).

7. D. Sengupta, A. Choudhury, C. Fortes-Lima, S. Aron, G. Whitelaw, K. Bostoen, H. Gunnink, N. Chousou-Polydouri, P. Delius, S. Tollman, F. X. Gómez-Olivé, S. Norris, F. Mashinya, M. Alberts, S. Hazelhurst, C. M. Schlebusch, M. Ramsay, Genetic substructure and complex demographic history of south African bantu speakers. *Nat. Commun.* **12**, 2080 (2021).

8. S. López, A. Tarekegn, G. Band, L. van Dorp, N. Bird, S. Morris, T. Oljira, E. Mekonnen, E. Bekele, R. Blench, M. G. Thomas, N. Bradman, G. Hellenthal, Evidence of the interplay of genetics and culture in Ethiopia. *Nat. Commun.* **12**, 3581 (2021).

9. L. van Dorp, D. Balding, S. Myers, L. Pagani, C. Tyler-Smith, E. Bekele, A. Tarekegn, M. G. Thomas, N. Bradman, G. Hellenthal, Evidence for a common origin of blacksmiths and cultivators in the Ethiopian Ari within the last 4500 years: Lessons for clustering-based inference. *PLOS Genet.* **11**, e1005397 (2015).

10. C. M. Schlebusch, H. Malmström, T. Günther, P. Sjödin, A. Coutinho, H. Edlund, A. R. Munters, M. Steyn, H. Soodyall, M. Lombard, M. Jakobsson, Ancient genomes from southern Africa pushes modern human divergence beyond 260,000 years ago. *Science* **358**, 652–655 (2017).

11. P. Skoglund, J. C. Thompson, M. E. Prendergast, A. Mittnik, K. Sirak, M. Hajdinjak, T. Salie, N. Rohland, S. Mallick, A. Peltzer, A. Heinze, I. Olalde, M. Ferry, E. Harney, M. Michel, K. Stewardson, J. I. Cerezo-Román, C. Chiumia, A. Crowther, E. Gomani-Chindebvu, A. O. Gidna, K. M. Grillo, I. T. Helenius, G. Hellenthal, R. Helm, M. Horton, S. López, A. Z. P. Mabulla, J. Parkington, C. Shipton, M. G. Thomas, R. Tibesasa, M. Welling, V. M. Hayes, D. J. Kennett, R. Ramesar, M. Meyer, S. Pääbo, N. Patterson, A. G. Morris, N. Boivin, R. Pinhasi, J. Krause, D. Reich, Reconstructing prehistoric African population structure. *Cell* **171**, 59–71.e21 (2017).

12. M. Lipson, I. Ribot, S. Mallick, N. Rohland, I. Olalde, N. Adamski, N. Broomandkhoshbacht, A. M. Lawson, S. López, J. Oppenheimer, K. Stewardson, R. N. Asombang, H. Bocherens, N. Bradman, B. J. Culleton, E. Cornelissen, I. Crevecoeur, P. de Maret, F. L. M. Fomine, P. Lavachery, C. M. Mindzie, R. Orban, E. Sawchuk, P. Semal, M. G. Thomas, W. Van Neer, K. R. Veeramah, D. J. Kennett, N. Patterson, G. Hellenthal, C. Lalueza-Fox, S. MacEachern, M. E. Prendergast, D. Reich, Ancient west African foragers in the context of African population history. *Nature* **577**, 665–670 (2020).

13. M. E. Prendergast, M. Lipson, E. A. Sawchuk, I. Olalde, C. A. Ogola, N. Rohland, K. A. Sirak, N. Adamski, R. Bernardos, N. Broomandkhoshbacht, K. Callan, B. J. Culleton, L. Eccles, T. K. Harper, A. M. Lawson, M. Mah, J. Oppenheimer, K. Stewardson, F. Zalzala, S. H. Ambrose, G. Ayodo, H. L. Gates, A. O. Gidna, M. Katongo, A. Kwekason, A. Z. P. Mabulla, G. S. Mudenda, E. K. Ndiema, C. Nelson, P. Robertshaw, D. J. Kennett, F. K. Manthi, D. Reich, Ancient DNA reveals a multistep spread of the first herders into sub-Saharan Africa. *Science* **364**, eaaw6275 (2019).

14. M. Lipson, E. A. Sawchuk, J. C. Thompson, J. Oppenheimer, C. A. Tryon, K. L. Ranhorn, K. M. de Luna, K. A. Sirak, I. Olalde, S. H. Ambrose, J. W. Arthur, K. J. W Arthur, G. Ayodo, A. Bertacchi, J. I. Cerezo-Román, B. J. Culleton, M. C. Curtis, J. Davis, A. O. Gidna, A. Hanson, P. Kaliba, M. Katongo, A. Kwekason, M. F. Laird, J. Lewis, A. Z. P Mabulla, F. Mapemba, A. Morris, G. Mudenda, R. Mwafulirwa, D. Mwangomba, E. Ndiema, C. Ogola, F. Schilt, P. R. Willoughby, D. K. Wright, A. Zipkin, R. Pinhasi, D. J. Kennett, F. Kyalo Manthi,

N. Rohland, N. Patterson, D. Reich, M. E. Prendergast, Ancient DNA and deep population structure in sub-Saharan African foragers. *Nature* **603**, 290–296 (2022).

15. G. B. Busby, G. Band, Q. Si Le, M. Jallow, E. Bougama, V. D. Mangano, L. N. Amenga-Etego, A. Enimil, T. Apinjoh, C. M. Ndila, A. Manjurano, V. Nyirongo, O. Doumba, K. A. Rockett, D. P. Kwiatkowski, C. C. Spencer; Malaria Genomic Epidemiology Network, Admixture into and within sub-Saharan Africa. *eLife* **5**, e15266 (2016).

16. N. Hollfelder, C. M. Schlebusch, T. Günther, H. Babiker, H. Y. Hassan, M. Jakobsson, Northeast African genomic variation shaped by the continuity of indigenous groups and Eurasian migrations. *PLOS Genet.* **13**, e1006976 (2017).

17. C. Fortes-Lima, P. Tříska, M. Čížková, E. Podgorná, M. Y. Diallo, C. M. Schlebusch, V. Černý, Demographic and selection histories of populations across the Sahel/Savannah belt. *Mol. Biol. Evol.* **39**, msac209 (2022).

18. L. Pagani, T. Kivisild, A. Tarekegn, R. Ekong, C. Plaster, I. Gallego Romero, Q. Ayub, S. Q. Mehdi, M. G. Thomas, D. Luiselli, E. Bekele, N. Bradman, D. J. Balding, C. Tyler-Smith, Ethiopian genetic diversity reveals linguistic stratification and complex influences on the Ethiopian gene pool. *Am. J. Hum. Genet.* **91**, 83–96 (2012).

19. J. K. Pickrell, N. Patterson, P. R. Loh, M. Lipson, B. Berger, M. Stoneking, B. Pakendorf, D. Reich, Ancient west Eurasian ancestry in southern and eastern Africa. *Proc. Natl. Acad. Sci. U.S.A.* **111**, 2632–2637 (2014).

20. M. Vicente, E. Priehodová, I. Diallo, E. Podgorná, E. S. Poloni, V. Černý, C. M. Schlebusch, Population history and genetic adaptation of the Fulani nomads: Inferences from genome-wide data and the lactase persistence trait. *BMC Genomics* **20**, 915 (2019).

21. D. Shriner, C. N. Rotimi, Genetic history of Chad. *Am. J. Phys. Anthropol.* **167**, 804–812 (2018).

22. P. Triska, P. Soares, E. Patin, V. Fernandes, V. Cerny, L. Pereira, Extensive admixture and selective pressure across the Sahel Belt. *Genome Biol. Evol.* **7**, 3484–3495 (2015).

23. E. Patin, M. Lopez, R. Grollemund, P. Verdu, C. Harmant, H. Quach, G. Laval, G. H. Perry, L. B. Barreiro, A. Froment, E. Heyer, A. Massougbodji, C. Fortes-Lima, F. Migot-Nabias, G. Bellis, J. M. Dugoujon, J. B. Pereira, V. Fernandes, L. Pereira, L. Van Der Veen, P. Mouguiama-Daouda, C. D. Bustamante, J. M. Hombert, L. Quintana-Murci, Dispersals and genetic adaptation of Bantu-speaking populations in Africa and North America. *Science* **356**, 543–546 (2017).

24. G. Hellenthal, G. B. J. J. Busby, G. Band, J. F. Wilson, C. Capelli, D. Falush, S. Myers, A genetic atlas of human admixture history. *Science* **343**, 747–751 (2014).

25. M. Salter-Townshend, S. Myers, Fine-scale inference of ancestry segments without prior knowledge of admixing groups. *Genetics* **212**, 869–889 (2019).

26. P. R. Loh, M. Lipson, N. Patterson, P. Moorjani, J. K. Pickrell, D. Reich, B. Berger, Inferring admixture histories of human populations using linkage disequilibrium. *Genetics* **193**, 1233–1254 (2013).

27. P. Wangkumhang, M. Greenfield, G. Hellenthal, An efficient method to identify, date, and describe admixture events using haplotype information. *Genome Res.* **32**, 1553–1564 (2022).

28. L. van Dorp, S. Lowes, J. L. Weigel, N. Ansari-Pour, S. López, J. Mendoza-Revilla, J. A. Robinson, J. Henrich, M. G. Thomas, N. Nunn, G. Hellenthal, Genetic legacy of state centralization in the Kuba kingdom of the Democratic Republic of the Congo. *Proc. Natl. Acad. Sci. U.S.A.* **116**, 593–598 (2018).

29. S. Leslie, B. Winney, G. Hellenthal, D. Davison, A. Boumertit, T. Day, K. Hutnik, E. C. Royrvik, B. Cunliffe, D. J. Lawson, D. Falush, C. Freeman, M. Pirinen, S. Myers, M. Robinson, P. Donnelly, W. Bodmer, The fine-scale genetic structure of the British population. *Nature* **519**, 309–314 (2015).

30. A. Semo, M. Gayà-Vidal, C. Fortes-Lima, B. Alard, S. Oliveira, J. Almeida, A. Prista, A. Damasceno, A. M. Fehn, C. Schlebusch, J. Rocha, Along the Indian Ocean coast: Genomic

variation in Mozambique provides new insights into the bantu expansion. *Mol. Biol. Evol.* **37**, 406–416 (2020).

31. A. A. Zaidi, I. Mathieson, Demographic history mediates the effect of stratification on polygenic scores. *eLife* **9**, e61548 (2020).

32. B. J. Vilhjálmsson, J. Yang, H. K. Finucane, A. Gusev, S. Lindström, S. Ripke, G. Genovese, P. R. Loh, G. Bhatia, R. Do, T. Hayeck, H. H. Won; Schizophrenia Working Group of the Psychiatric Genomics Consortium, Discovery, Biology, and Risk of Inherited Variants in Breast Cancer (DRIVE) study, S. Kathiresan, M. Pato, C. Pato, E. Stahl, N. Zaitlen, B. Pasaniuc, E. E. Kenny, M. H. Schierup, P. De Jager, N. A. Patsopoulos, S. McCarroll, M. Daly, S. Purcell, D. Chasman, B. Neale, M. Goddard, N. Patterson, A. L. Price, Modeling linkage disequilibrium increases accuracy of polygenic risk scores. *Am. J. Hum. Genet.* **97**, 576–592 (2015).

33. H. Hammarström, R. Forkel, M. Haspelmath, S. Bank, "Glottolog 4.5" (Max Planck Institute for Evolutionary Anthropology, 2021); https://doi.org/10.5281/zenodo.5772642.

34. R. G. Gordon, B. F. Grimes, *Ethnologue: Languages of the World* (SIL International, 2005).

35. I. Fowler, D. Zeitlyn, *African Crossroads: Intersections Between History and Anthropology in Cameroon* (Bergahn Books, 1996).

36. R. Blench, in *Nuba Mountain Language Studies* (Rudiger Koppe, 2013), pp. 571–586.

37. R. Oliver, J. D. Fage, *The Cambridge History of Africa* (Cambridge Univ. Press, 1977).

38. V. Hiribarren, in *The Encyclopedia of Empire* (John Wiley & Sons Ltd., 2016), pp. 1–6.

39. C. Ehret, The Nilo-Saharan background of Chadic. *Stud. African Linguist.* **35**, 56–66 (2006).

40. J. Vansina, New linguistic evidence and 'the bantu expansion'. *J. Afr. Hist.* **36**, 173–195 (1995).

41. I. Lazaridis, N. Patterson, A. Mittnik, G. Renaud, S. Mallick, K. Kirsanow, P. H. Sudmant, J. G. Schraiber, S. Castellano, M. Lipson, B. Berger, C. Economou, R. Bollongino, Q. Fu, K. I. Bos, S. Nordenfelt, H. Li, C. De Filippo, K. Prüfer, S. Sawyer, C. Posth, W. Haak, F. Hallgren, E. Fornander, N. Rohland, D. Delsate, M. Francken, J. M. Guinet, J. Wahl, G. Ayodo, H. A. Babiker, G. Bailliet, E. Balanovska, O. Balanovsky, R. Barrantes, G. Bedoya, H. Ben-Ami, J. Bene, F. Berrada, C. M. Bravi, F. Brisighelli, G. B. J. Busby, F. Cali, M. Churnosov, D. E. C. Cole, D. Corach, L. Damba, G. Van Driem, S. Dryomov, J. M. Dugoujon, S. A. Fedorova, I. Gallego Romero, M. Gubina, M. Hammer, B. M. Henn, T. Hervig, U. Hodoglugil, A. R. Jha, S. Karachanak-Yankova, R. Khusainova, E. Khusnutdinova, R. Kittles, T. Kivisild, W. Klitz, V. Kučinskas, A. Kushniarevich, L. Laredj, S. Litvinov, T. Loukidis, R. W. Mahley, B. Melegh, E. Metspalu, J. Molina, J. Mountain, K. Näkkäläjärvi, D. Nesheva, T. Nyambo, L. Osipova, J. Parik, F. Platonov, O. Posukh, V. Romano, F. Rothhammer, I. Rudan, R. Ruizbakiev, H. Sahakyan, A. Sajantila, A. Salas, E. B. Starikovskaya, A. Tarekegn, D. Toncheva, S. Turdikulova, I. Uktveryte, O. Utevska, R. Vasquez, M. Villena, M. Voevoda, C. A. Winkler, L. Yepiskoposyan, P. Zalloua, T. Zemunik, A. Cooper, C. Capelli, M. G. Thomas, A. Ruiz-Linares, S. A. Tishkoff, L. Singh, K. Thangaraj, R. Villems, D. Comas, R. Sukernik, M. Metspalu, M. Meyer, E. E. Eichler, J. Burger, M. Slatkin, S. Pääbo, J. Kelso, D. Reich, J. Krause, Ancient human genomes suggest three ancestral populations for present-day Europeans. *Nature* **513**, 409–413 (2014).

42. X. Zheng-Bradley, I. Streeter, S. Fairley, D. Richardson, L. Clarke, P. Flicek, Alignment of 1000 genomes project reads to reference assembly GRCh38. *Gigascience* **6**, 1–8 (2017).

43. M. Byrska-Bishop, U. S. Evani, X. Zhao, A. O. Basile, H. J. Abel, High coverage whole genome sequencing of the expanded 1000 genomes project cohort including 602 trios. *SSRN Electron. J.* 10.2139/ssrn.3967671 (2021).

44. Malaria Genomic Epidemiology Network, Insights into malaria susceptibility using genome-wide data on 17,000 individuals from Africa, Asia and Oceania. *Nat. Commun.* **10**, 5732 (2019).

45. S. López, M. G. Thomas, L. van Dorp, N. Ansari-Pour, S. Stewart, A. L. Jones, E. Jelinek, L. Chikhi, T. Parfitt, N. Bradman, M. E. Weale, G. Hellenthal, The genetic legacy of Zoroastrianism in Iran and India: Insights into population structure, gene flow, and selection. *Am. J. Hum. Genet.* **101**, 353–368 (2017).

46. N. Patterson, A. L. Price, D. Reich, Population structure and eigenanalysis. *PLOS Genet.* **2**, 2074–2093 (2006).

47. D. J. Lawson, G. Hellenthal, S. Myers, D. Falush, Inference of population structure using dense haplotype data. *PLOS Genet.* **8**, 11–17 (2012).

48. D. H. Alexander, K. Lange, Enhancements to the ADMIXTURE algorithm for individual ancestry estimation. *BMC Bioinformatics* **12**, 246 (2011).

49. Y. Zhou, S. R. Browning, B. L. Browning, A fast and simple method for detecting identity-by-descent segments in large-scale data. *Am. J. Hum. Genet.* **106**, 426–437 (2020).

50. J. C. Chacón-Duque, K. Adhikari, M. Fuentes-Guajardo, J. Mendoza-Revilla, V. Acuña-Alonzo, R. B. Lozano, M. Quinto-Sánchez, J. Gómez-Valdés, P. E. Martínez, H. Villamil-Ramírez, T. Hünemeier, V. Ramallo, C. C. S. de Cerqueira, M. Hurtado, V. Villegas, V. Granja, M. Villena, R. Vásquez, E. Llop, J. R. Sandoval, A. A. Salazar-Granara, M.-L. Parolin, K. Sandoval, R. Peñaloza-Espinosa, H. Rangel-Villalobos, C. Winkler, W. Klitz, C. Bravi, J. Molina, D. Corach, R. Barrantes, V. Gomes, C. Resende, L. Gusmão, A. Amorim, Y. Xue, J.-M. Dugoujon, P. Moral, R. Gonzalez-José, L. Schuler-Faccini, F. M. Salzano, M.-C. Bortolini, S. Canizales-Quinteros, G. Poletti, C. Gallo, G. Bedoya, F. Rothhammer, D. Balding, G. Hellenthal, A. Ruiz-Linares, Latin Americans show wide-spread Converso ancestry and the imprint of local native ancestry on physical appearance. *Nat. Commun.* **9**, 1–22 (2018).

51. M. K. H. Eggert, The Bantu problem and African archaeology, in *African Archaeology* (2005), pp. 301–326.

52. R. Blench, *Archaeology, Language, and the African Past* (Alta Mira Press, 2006).

53. D. Seidensticker, W. Hubau, D. Verschuren, C. Fortes-Lima, P. de Maret, C. M. Schlebusch, K. Bostoen, Population collapse in Congo rainforest from 400 CE urges reassessment of the bantu expansion. *Sci. Adv.* **7**, eabd8352 (2021).

54. G. de Saulieu, Y. Garcin, D. Sebag, P. R. N. Nlend, D. Zeitlyn, P. Deschamps, G. Ménot, P. Di Carlo, R. Oslisly, Archaeological evidence for population rise and collapse between ~2500 and ~500 cal. yr BP in Western Central Africa. *Afrique Archeol. Arts* 11–32 (2021).

55. E. Santiago, I. Novo, A. F. Pardiñas, M. Saura, J. Wang, A. Caballero, Recent demographic history inferred by high-resolution analysis of linkage disequilibrium. *Mol. Biol. Evol.* **37**, 3642–3653 (2020).

56. S. R. Browning, B. L. Browning, Accurate non-parametric estimation of recent effective population size from segments of identity by descent. *Am. J. Hum. Genet.* **97**, 404–418 (2015).

57. J. C. Anene, The Nigeria—Southern Cameroons boundary (an ethno-political analysis). *J. Hist. Soc. Niger.* **2**, 186–195 (1961).

58. F. L. C. Jackson, So many Nigerians: Why is Nigeria overrepresented as the ancestral genetic homeland of legacy African north Americans? *Am. J. Hum. Genet.* **108**, 202–208 (2021).

59. M. Krings, A. E. H. Salem, K. Bauer, H. Geisert, A. K. Malek, L. Chaix, C. Simon, D. Welsby, A. Di Rienzo, G. Utermann, A. Sajantila, S. Pääbo, M. Stoneking, mtDNA analysis of Nile River valley populations: A genetic corridor or a barrier to migration? *Am. J. Hum. Genet.* **64**, 1166–1176 (1999).

60. I. Fowler, Kingdoms of the Cameroon Grassfields. *Rev. Anthropol.* **40**, 292–311 (2011).

61. I. Kopytoff, in *Beyond Chiefdoms: Pathways to Complexity in Africa*, S. Keech McIntosh, Ed. (Cambridge Univ. Press, 1999), pp. 88–96.

62. J.-P. Warnier, thesis (1975).

63. J. Nfi, Intercommunity relations and the politics of identity in the Nso chiefdom, Bamenda Grassfields, Cameroon. *J. Res. Peace Gend. Dev.* **4**, 63–69 (2014).

64. K. R. Veeramah, D. Zeitlyn, V. G. Fanso, N. R. Mendell, B. A. Connell, M. E. Weale, N. Bradman, M. G. Thomas, Sex-specific genetic data support one of two alternative versions of the foundation of the ruling dynasty of the Nso' in Cameroon. *Curr. Anthropol.* **49**, 707–714 (2008).

65. R. K. Engard, Myth and political economy in Bafut (Cameroon): The structural history of an African kingdom. *Paid. Mitteilungen zur Kult.* **34**, 49–89 (1988).

66. R. Thelwall, T. C. Schadeberg, The linguistic settlement of the Nuba Mountains. *SUGIA* **5**, 219–231 (1983).

67. R. Hoyland, *In God's Path: The Arab Conquests and the Creation of an Islamic Empire* (Oxford Univ. Press, 2014).

68. S. Munro-Hay, The foreign trade of the Aksumite port of Adulis. *Azania Archaeol. Res. Africa.* **17**, 107–125 (1982).

69. P. Wangkumhang, G. Hellenthal, Statistical methods for detecting admixture. *Curr. Opin. Genet. Dev.* **53**, 121–127 (2018).

70. U. Braukamper, *Notes on the Origin of Baggara Arab Culture with Special Reference to the Shuwa* (Köppe, 1993).

71. B. Barkindo, D. Lange, in *Unesco General History of Africa, Volume III. Africa from the Seventh to the Eleventh Century*, M. Elfasi, I. Hrbek, Eds. (Heinemann, 1988), pp. 436–460.

72. R. Oliver, B. M. Fagan, *Africa in the Iron Age: C.500BC-1400AD* (Cambridge Univ. Press, 1975).

73. M. Hiskett, *The Sword of Truth: The Life and Times of the Shehu Usuman dan Fodio* (Northwestern Univ. Press, 1994).

74. S. MacEachern, in *Burials, Migration and Identity in the Ancient Sahara and Beyond,* M. Carmela Gatto, D. Mattingly, N. Ray, M. Sterry, Eds. (Cambridge Univ. Press, 2019), pp. 399–428.

75. G. Philippson, R. Grollemund, in *The Bantu Languages* (Routledge, 2003).

76. K. Bostoen, in *Oxford Research Encyclopedia of African History* (Oxford Univ. Press, 2018), pp. 1–26.

77. E. Patin, K. J. Siddle, G. Laval, H. Quach, C. Harmant, N. Becker, A. Froment, B. Régnault, L. Lemée, S. Gravel, J. M. Hombert, L. Van Der Veen, N. J. Dominy, G. H. Perry, L. B. Barreiro, P. Verdu, E. Heyer, L. Quintana-Murci, The impact of agricultural emergence on the genetic history of African rainforest hunter-gatherers and agriculturalists. *Nat. Commun.* **5**, 3163 (2014).

78. K. Bostoen, B. Clist, C. Doumenge, R. Grollemund, J. M. Hombert, J. K. Muluwa, J. Maley, Middle to late holocene paleoclimatic change and the early bantu expansion in the rain forests of Western Central Africa. *Curr. Anthropol.* **56**, 354–384 (2015).

79. M. H. Gouveia, A. W. Bergen, V. Borda, K. Nunes, T. P. Leal, M. D. Ogwang, E. D. Yeboah, J. E. Mensah, T. Kinyera, I. Otim, H. Nabalende, I. D. Legason, S. W. Mpoloka, G. G. Mokone, P. Kerchan, K. Bhatia, S. J. Reynolds, R. B. Birtwum, A. A. Adjei, Y. Tettey, E. Tay, R. Hoover, R. M. Pfeiffer, R. J. Biggar, J. J. Goedert, L. Prokunina-Olsson, M. Dean, M. Yeager, M. F. Lima-Costa, A. W. Hsing, S. Tishkoff, S. J. Chanock, E. Tarazona-Santos, S. M. Mbulaiteye, Genetic signatures of gene flow and malaria-driven natural selection in sub-Saharan populations of the “endemic Burkitt lymphoma belt” *PLOS Genet.* **15**, e1008027 (2019).

80. T. M. Shanahan, J. T. Overpeck, C. W. Wheeler, J. W. Beck, J. S. Pigati, M. R. Talbot, C. A. Scholz, J. Peck, J. W. King, Paleoclimatic variations in West Africa from a record of late Pleistocene and Holocene Lake level stands of Lake Bosumtwi, Ghana. *Palaeogeogr. Palaeoclimatol. Palaeoecol.* **242**, 287–302 (2006).

81. A. Vincens, D. Schwartz, H. Elenga, I. Reynaud-Farrera, A. Alexandre, J. Bertaux, A. Mariotti, L. Martin, J. D. Meunier, F. Nguetsop, M. Servant, S. Servant-Vildary, D. Wirrmann, Forest response to climate changes in Atlantic equatorial Africa during the last 4000 years BP and inheritance on the modern landscapes. *J. Biogeogr.* **26**, 879–885 (1999).

82. C. J. E. Ingram, R. Ekong, N. Ansari-Pour, N. Bradman, D. M. Swallow, Group-based pharmacogenetic prediction: Is it feasible and do current NHS England ethnic classifications provide appropriate data? *Pharmacogenomics J.* **21**, 47–59 (2020).

83. V. Link, A. Kousathanas, K. Veeramah, C. Sell, A. Scheu, D. Wegmann, ATLAS: Analysis tools for low-depth and ancient samples. bioRxiv 105346 [**Preprint**]. 2 February 2017. https://doi.org/10.1101/105346.

84. B. L. Browning, S. R. Browning, Genotype imputation with millions of reference samples. *Am. J. Hum. Genet.* **98**, 116–126 (2016).

85. O. Delaneau, J. F. Zagury, M. R. Robinson, J. L. Marchini, E. T. Dermitzakis, Accurate, scalable and integrative haplotype estimation. *Nat. Commun.* **10**, 5436 (2019).

86. C. C. Chang, C. C. Chow, L. C. Tellier, S. Vattikuti, S. M. Purcell, J. J. Lee, Second-generation PLINK: Rising to the challenge of larger and richer datasets. *Gigascience* **4**, 7 (2015).

87. J. Oksanen, F. G. Blanchet, M. Friendly, R. Kindt, P. Legendre, D. Mcglinn, P. R. Minchin, R. B. O'hara, G. L. Simpson, P. Solymos, M. Henry, H. Stevens, E. Szoecs, H. W. Maintainer, "Package "vegan" title community ecology package version 2.5-7" (2020).

88. J. N. Fenner, Cross-cultural estimation of the human generation interval for use in genetics-based population divergence studies. *Am. J. Phys. Anthropol.* **128**, 415–423 (2005).

89. J. K. Pickrell, N. Patterson, C. Barbieri, F. Berthold, L. Gerlach, T. Güldemann, B. Kure, S. W. Mpoloka, H. Nakagawa, C. Naumann, M. Lipson, P. R. Loh, J. Lachance, J. Mountain, C. D. Bustamante, B. Berger, S. A. Tishkoff, B. M. Henn, M. Stoneking, D. Reich, B. Pakendorf, The genetic prehistory of southern Africa. *Nat. Commun.* **3**, 1143 (2012).

90. F. Baumdicker, G. Bisschop, D. Goldstein, G. Gower, A. P. Ragsdale, G. Tsambos, S. Zhu, B. Eldon, E. C. Ellerman, J. G. Galloway, A. L. Gladstein, G. Gorjanc, B. Guo, B. Jeffery, W. W. Kretzschumar, K. Lohse, M. Matschiner, D. Nelson, N. S. Pope, C. D. Quinto-Cortes, M. F. Rodrigues, K. Saunack, T. Sellinger, K. Thornton, H. Van Kemenade, A. W. Wohns, Y. Wong, S. Gravel, A. D. Kern, J. Koskela, P. L. Ralph, J. Kelleher, Efficient ancestry and mutation simulation with msprime 1.0. *Genetics* **220**, iyab229 (2022).

91. J. Spaulding, Medieval christian Nubia and the islamic world: A reconsideration of the *baqt* treaty. *Int. J. Afr. Hist. Stud.* **28**, 577–594 (1995).

92. M. J. Harrower, J. C. Mazzariello, A. C. D'Andrea, S. Nathan, H. M. Taddesse, I. A. Dumitru, C. E. Priebe, K. Zerue, Y. Park, G. Gebreegziabher, Aksumite settlement patterns: Site size hierarchies and spatial clustering. *J. Archaeol. Res.* **31**, 103–146 (2023).

93. G. Hellenthal, A. Auton, D. Falush, Inferring human colonization history using a copying model. *PLOS Genet.* **4**, e1000078 (2008).
