# Supplementary material for: Dense sampling of ethnic groups within African countries reveals fine-scale genetic structure and extensive historical admixture
Source: Sci Adv. 2023 Mar 29;9(13):eabq2616. doi: 10.1126/sciadv.abq2616 (PMC10058250; doi:10.1126/sciadv.abq2616)
Supplement: Supplementary file 2 — Fig. S6 [file sciadv.abq2616_figure_s6.zip › abq2616_Figure_S6.pdf]

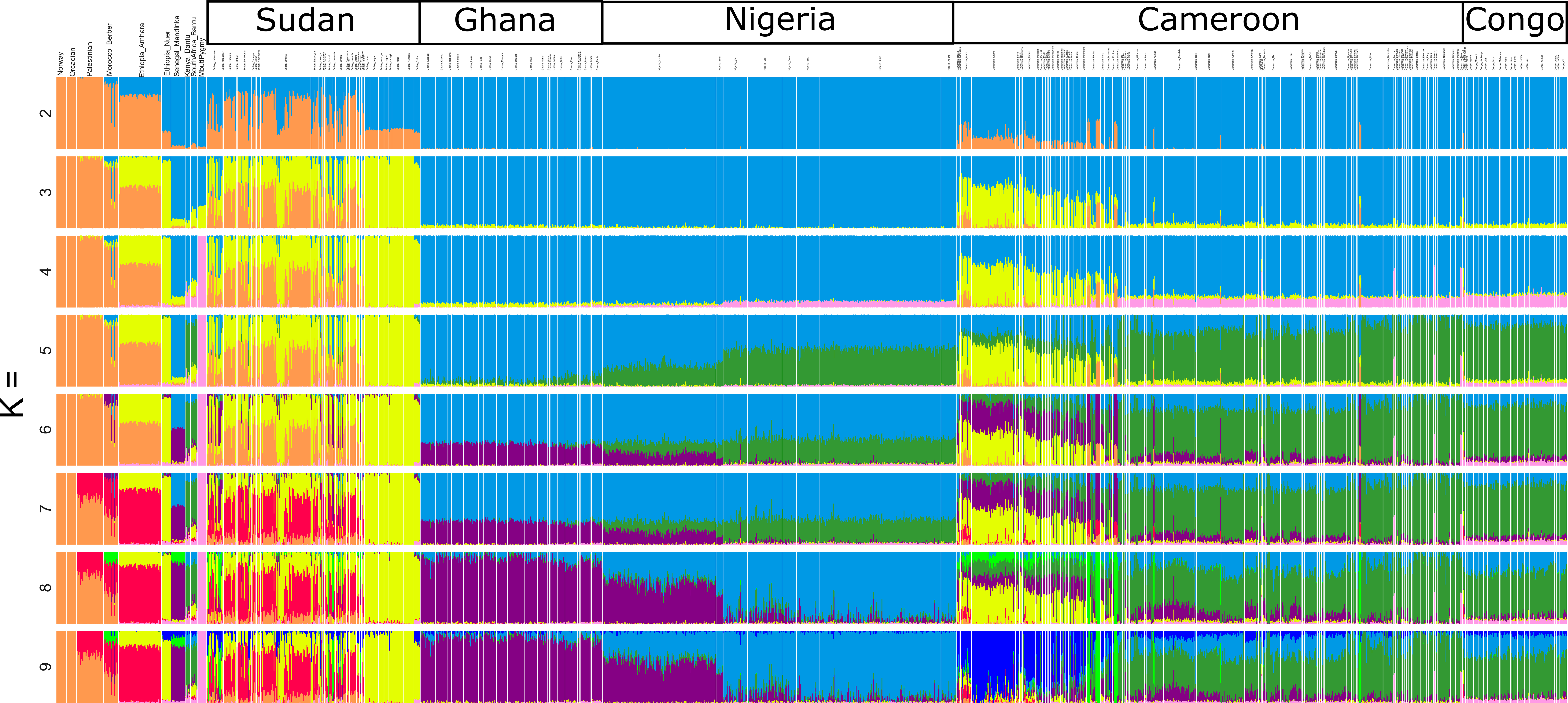

Sudan
Ghana
Nigeria
Cameroon
Congo
Norway
Orcadian
Palestinian
Morocco_Berber
Ethiopia_Amhara
Ethiopia_Nuer
Senegal_Mandinka
Kenya_Bantu
SouthAfrica_Bantu
MbutiPygmy
K =
2
3
4
5
6
7
8
9
